# Cluster-Based Thermodynamics of Interacting Dice in a Lattice

**DOI:** 10.3390/e22101111

**Published:** 2020-10-01

**Authors:** Christoph Mayer, Thomas Wallek

**Affiliations:** Institute of Chemical Engineering and Environmental Technology, Graz University of Technology, 8010 Graz, Austria; cmayer@tugraz.at

**Keywords:** Shannon entropy, discrete modeling, lattice model, dice model, cluster, cooperative effects, activity coefficients

## Abstract

In this paper, a model for two-component systems of six-sided dice in a simple cubic lattice is developed, based on a basic cluster approach previously proposed. The model represents a simplified picture of liquid mixtures of molecules with different interaction sites on their surfaces, where each interaction site can be assigned an individual energetic property to account for cooperative effects. Based on probabilities that characterize the sequential construction of the lattice using clusters, explicit expressions for the Shannon entropy, synonymously used as thermodynamic entropy, and the internal energy of the system are derived. The latter are used to formulate the Helmholtz free energy that is minimized to determine thermodynamic bulk properties of the system in equilibrium. The model is exemplarily applied to mixtures that contain distinct isomeric configurations of molecules, and the results are compared with the Monte-Carlo simulation results as a benchmark. The comparison shows that the model can be applied to distinguish between isomeric configurations, which suggests that it can be further developed towards an excess Gibbs-energy, respectively, activity coefficient model for chemical engineering applications.

## 1. Introduction

The fluid-phase properties of mixtures need to be accurately estimated to, e.g., perform precise phase equilibria and process engineering calculations, design pharmaceutical ingredients, and tailor chemicals in order to obtain the desired physical and chemical properties, to name but a few purposes. In this context, and notably against the background of the upcoming change in the raw materials base to establish bio-based value chains, complex molecules are particularly challenging. Their distinctly non-spherical shapes and diverse functional groups in the molecular structure prevent the use of established methods and models in order to reliably estimate the macroscopic thermodynamic properties of such mixtures of molecules.

To make such estimations, basically two approaches can be chosen: on one hand, many physico-chemical and transport properties can directly be determined by running molecular simulations; however, such simulations require considerable amounts of simulation time as well as expertise in order to determine the required force field parameters, which are the basis of successful simulations. On the other hand, a simplified picture of real molecules can be used to develop thermodynamic models to describe the bulk properties of mixtures. These include in particular excess Gibbs-energy ($g^{E}$) models or, synonymously, activity coefficients that are broadly implemented in commercial process simulation tools.

Such thermodynamic models mostly consider non-spherical molecular shapes and/or intermolecular interaction forces as the causes of deviations from random mixing behavior. Due to the latter, the molecules are not statistically dependent and, consequently, it is not possible to treat single molecules as statistically independent subsystems in the sense of statistical mechanics. Various approximations at different levels of complexity have been proposed in previous model development in order to take this into account.

A frequently used approach are pair approximations, which assume the statistical independence of contact pairs. These form the basis of a number of so-called quasichemical models, starting with Guggenheim [1,2,3] and continuing with more recent models that are frequently used in chemical engineering applications, like UNIFAC [4,5], GEQUAC [6], MOQUAC [7], COSMO-RS resp. COSMOTHERM [8,9,10,11,12], and PAC-MAC [13,14,15,16]. One common simplifying assumption used in quasichemical models is that surface segments are decoupled, and only the number densities of surface fragments are used. Consequently, these models can also be applied to consider arrangements of molecules that are geometrically not possible, which limits or even inhibits the distinction between isomeric configurations. One example for an approach that introduces geometric information a posteriori is the COSMO-RSC model, accounting for the second-order effects that recover local surface correlation effects [17].

Various approaches are taken that use clusters of molecules as a basis for modeling in order to improve the accuracy provided by models using statistically independent contact pairs.

The binary quantum cluster equilibrium (bQCE) theory [18,19,20,21,22,23] uses clusters as a basis for estimating the activity coefficients of liquid mixtures, whereby the liquid phase is described by a so-called cluster gas, and the formation of the clusters from monomers is modeled like a chemical reaction.

Akkermans [24] combined a Flory–Huggins approach with a force field-based molecular sampling algorithm to determine interaction energies for multiple cluster configurations as the input for a thermodynamic model that was then used to calculate the cluster free energy based on statistical thermodynamics using integration.

More sophisticated theories, such as the reference interaction site model (RISM) [25,26] and lattice cluster theory (LCT) approaches [27,28], were developed to treat problems that exceed Flory–Huggins’s original approximations. The latter is based on a lattice model in which united atom groups, rather than entire monomers, occupy a lattice site.

The so-called cluster variation methods (CVM) [29,30,31] were significant improvements over pair approximations and they have been well-engineered for the modeling of moderate size clusters [32]. They are commonly applied to two-dimensional and three-dimensional, face-centered, cubic systems to predict phase diagrams for alloys [33]. However, CVM models have limitations due to their complexity and immense number of variables with increasing cluster size, which require the use of algorithms that have been specially tailored to the structure of CVM equations and that must be solved in the course of the equilibration [34,35]. Probably for these reasons, CVM approaches have not been adapted for activity coefficent models in chemical engineering applications.

A substantially different approach was taken by Vinograd et al., who used the Markov-chain theory for the hypothetical, sequential construction of a lattice [36,37], focusing on the prediction of two-dimensional crystal structures. In this approach, which can be understood as an approximation of CVM methods, the system’s entropy is modeled as the Shannon information of clusters that are formed in the course of the sequential lattice construction. By using clusters of molecules, this approach intrinsically accounts for the geometric constraints that affect the intermolecular interactions between surface segments and, thus, considers solely geometrically realistic cluster states. When compared to most quasichemical approaches, which are based on the simplified assumption that surface segments are decoupled, this a priori consideration of geometric information is seen as a major advantage of the cluster approach.

As a continuation of our previous work [38,39,40,41], in this paper, the basic cluster approach proposed by Vinograd [36,37] is extended to two-component systems of six-sided dice in a simple cubic lattice.

Dice models have been widely used in the context of kinetic theory and statistical thermodynamics in order to illustrate basic terms like realisation possibilities, probabilities, microstates and macrostates, and so forth. In particular, Boltzmann used analogies from dice games in various contexts, such as the mean free path of molecules within the theory of gases [42,43,44], the *H*-theorem [44], time irreversibility and the second law [44], and the theory of heat [45], to name but a few examples. Furthermore, dice games have always been used in connection with the interpretation of entropy [46,47]. In the context of this paper, the dice represent a simplified model of liquid mixtures of molecules with different interaction sites on their surfaces, for which the Shannon entropy can be approximated by combinatorial and probabilistic considerations as the basis of a thermodynamic model.

The paper is structured, as follows. First, based on probabilities that characterize the sequential construction of a lattice with clusters, explicit expressions for the Shannon entropy, synonymously used as thermodynamic entropy, and the internal energy of the system are derived. The latter two are used to formulate the Helmholtz free energy, which should be minimized to determine the thermodynamic bulk properties of the system in equilibrium. Second, the constraints for this minimization are developed. Subsequently, the model is exemplarily applied to mixtures containing isomeric configurations of molecules, and the results are compared with those from the Monte-Carlo simulations as a benchmark. Finally, the comparison shows that the model can be applied to distinguish between isomeric configurations, which suggests that it can be further developed towards an excess Gibbs-energy, respectively, activity coefficient model for chemical engineering applications.

## 2. Model

In the following, a model for two-component systems of six-sided dice in a simple cubic lattice is developed in the variable space of entropy, internal energy, and molecule numbers, $\left\{S,U,N_{i}\right\}$. The volume is not taken into account, because one die occupies each site of the lattice, without vacancies. In this way, the model represents a simplified picture of liquid mixtures of molecules with different interaction sites on their surfaces in order to account for cooperative effects.

### 2.1. Modeling Concept

The key concept behind the approach presented in this work is a discrete modeling concept that is based on clusters. The term ’discrete modeling’ encompasses discrete states of molecules within their neighborhood of interaction, where a state is defined by the type and orientation of a molecule, as in our previous work. In this paper, a cluster of molecules is chosen as a representative part of the system. For this cluster, a finite number of possible constructions based on the component selection, orientation, and location is chosen. From then on, it is assumed that a cluster can only exist in one of these states. The entropy and internal energy of the system are then formulated using the probabilities of occurrence for each cluster in the system. Both of the functions are combined into the Helmholtz free energy, which is subject to constrained minimization, yielding the equilibrium distribution of the cluster probabilities. Bulk properties of the system can be determined once the latter has been obtained.

#### 2.1.1. Sequential Lattice Construction as a Discrete Markov Chain

**Figure 1:**
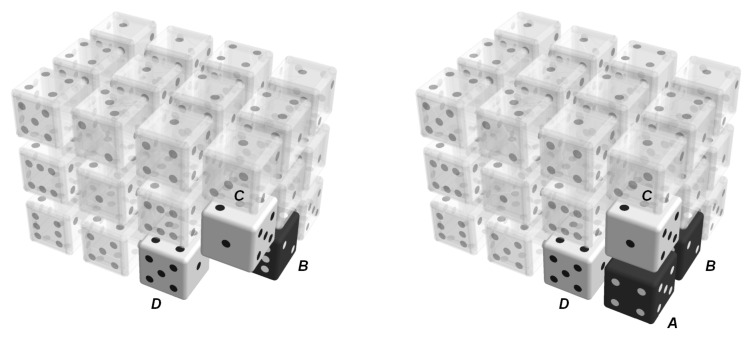
Sequential construction of the lattice as a discrete Markov chain by inserting a molecule at position *A* where the neighborhood at positions *B*, *C*, and *D* is already present as a result of previous insertion steps.

A core idea of the model is the sequential construction of the lattice as a discrete Markov chain, as introduced by Vinograd et al. [36,37]. One-by-one, the molecules are inserted into a partially existing neighborhood that has already been created by previous insertion steps, as illustrated in Figure 1.

One can see that such an insertion step involves four molecules that are arranged in such a way that three of the molecules are neighbors of the fourth ‘central’ one; each of the molecules is located along one of the three axes of the simple cubic lattice, respectively. Such a cluster of four molecules provides three nearest neighbors for the central molecule; these are relevant for cooperative effects due to energetic interactions, as will be shown later. The cluster shape is in accordance with the sequential construction, where the three neighboring molecules *B*, *C*, and *D* represent the state of the lattice before an insertion step, and the fourth molecule, *A*, represents the new molecule that has been inserted within this lattice.

#### 2.1.2. Set of Variables

The main variables used for modeling are the average probabilities of finding a cluster in a specific state within the equilibrium lattice. To stick with two-component systems that are illustrated by black and white dice, in this paper a specific state refers to each molecule being (i) either of component type 1 or 2 and (ii) in a certain orientation. For die-like molecules, where each of the six dice surfaces can be turned four times around its own axis, a maximum of 6×4=24 different orientations can occur. The probability of an entire cluster to be in a specific state is denoted as $p_{a\cdot b\cdot c\cdot d}$. Let $a^{*}$ be the set of all possible states that the molecule at position *A* in the cluster can be in. Then *a* is an element of the set $a^{*}$. Accordingly, *b*, *c*, and *d* are the states of the molecules that are located at positions *B*, *C*, and *D*. The insertion process itself is described by the conditional probability $p_{a|b\cdot c\cdot d}$. This describes the probability of inserting a molecule of state *a* into an existing neighborhood given by the states *b*, *c*, and *d*. The correlation between the cluster probabilities, $p_{a\cdot b\cdot c\cdot d}$, and the conditional probabilities, $p_{a|b\cdot c\cdot d}$, is given by the definition of conditional probabilities:

(1) $$p_{a|b\cdot c\cdot d}=\frac{p_{a\cdot b\cdot c\cdot d}}{p_{b\cdot c\cdot d}}\;.$$

In Equation (1), the denominator is the neighborhood probability, $p_{b\cdot c\cdot d}$, which is the probability of finding the three nearest neighbors of the central molecule in a particular state. The latter can be expressed as a so-called marginal probability of the entire cluster probability, while using the law of total probability:

(2) $$p_{b\cdot c\cdot d}=\sum_{a}p_{a\cdot b\cdot c\cdot d}\;.$$

In Equation (2), the sum over *a* is a shorthand notation for the sum over all possible states at position *A*, where $a\in a^{*}$.

### 2.2. Entropy of the System

Generally, in this paper, the Shannon information of the system [48], $H_{\mathrm{system}}$, is used as a synonym for the thermodynamic entropy of the system, $S_{\mathrm{system}}$, by multiplying the former by Boltzmann’s constant, $k_{B}$, and the number of molecules, *N*, in addition to choosing Euler’s constant as the base of the logarithm:

(3) $$H_{\mathrm{system}}=-\sum_{i}p_{i}^{\mathrm{system}}\log\left(p_{i}^{\mathrm{system}}\right)\;S_{\mathrm{system}}=-k_{B}N\;\sum_{i}p_{i}^{\mathrm{system}}\ln\left(p_{i}^{\mathrm{system}}\right)$$

In Equation (3), the variables $p_{i}^{\mathrm{system}}$ denote the probabilities that the system exists in a discrete state *i*. However, the modeling concept that is presented in Section 2.1 is not based on the probabilities of states of the system as a whole, but on probabilities of clusters as representative parts of the system. Consequently, Equation (3) cannot be directly used to formulate of the system’s entropy, but must first be adapted so that it can be formulated with cluster probabilities and conditional probabilities that reflect the sequential construction of the lattice, as described before. Starting from the relevant general properties of the entropy function [46,47,49], this adaption is outlined in the following section.

Let *p* and *q* be two probability distributions of two random variables, *Y* and *Z*, where these distributions are not necessarily independent and for which

(4) $$P\left(Y=y_{i}\right)=p_{i}\;,\;\mathrm{and}\;P\left(Z=z_{j}|Y=y_{i}\right)=q_{ij}$$

is true, where $y_{i}$ and $z_{i}$ represent particular values for the random variables *Y* and *Z*, respectively. For the joint distribution, p·q, the entropy is given by

(5) $$S\left(p\cdot q\right)=-\sum_{j=1}^{m}\sum_{i=1}^{n}p_{i}q_{ij}\ln\left(p_{i}q_{ij}\right)\;,$$

where m,n are the numbers of possible states for each of the two probability distributions. Equation (5) can be split into

(6) $$S\left(p\cdot q\right)=-\sum_{i=1}^{n}\left(p_{i}\ln\left(p_{i}\right)\;\sum_{j=1}^{m}q_{ij}\right)-\sum_{i=1}^{n}\left(p_{i}\sum_{j=1}^{m}q_{ij}\ln\left(q_{ij}\right)\right).$$

The sum over all possibilities for *Z* at a fixed state $y_{i}$ of *Y* must equal one, which can be expressed as

(7) $$\sum_{j=1}^{m}q_{ij}=\sum_{j=1}^{m}P\left(Z=z_{j}|Y=y_{i}\right)=1\;.$$

Thus, Equation (7) can be used to simplify Equation (6) to

(8) $$\begin{array}{lll} S(p\cdot q) & = & S\left(p\right)+\sum_{i=1}^{n}p_{i}S\left(q_{i}\right),\mathrm{or} \end{array}$$

(9) $$\begin{array}{lll} S(p\cdot q) & = & S(p)+S(q|p)\;. \end{array}$$

Now, applied to the system of die-like molecules, $p_{i}$ should be replaced by the neighborhood probability, $p_{b\cdot c\cdot d}$, and $q_{ij}$, by the conditional insertion probability, $p_{a|b\cdot c\cdot d}$. Consequently, S(p) can be interpreted as the neighborhood entropy and S(q|p) as the entropy of the insertion process. As the latter describes the sequential construction of the lattice, S(q|p) represents the entropy of the system as well. Finally, the entropy of the joint distribution, S(p·q), represents the cluster entropy. With that in mind, Equation (9) can be interpreted and rewritten as

(10) $$S_{\mathrm{cluster}}=S_{\mathrm{neighborhood}}+S_{\mathrm{system}}\;.$$

Applying Equation (10), the part of Equation (6) that calculates the system entropy can be extracted as

(11) $$S\left(q|p\right)=-\sum_{i=1}^{n}\left(p_{i}\sum_{j=1}^{m}q_{ij}\ln\left(q_{ij}\right)\right)\;.$$

Finally, by replacing $p_{i}$ by the neighborhood probability, $p_{b\cdot c\cdot d}$, and $q_{ij}$ by the conditional insertion probability, $p_{a|b\cdot c\cdot d}$, the entropy of the system can be written as

(12) $$S_{\mathrm{system}}=-k_{B}N\sum_{b}\sum_{c}\sum_{d}p_{b\cdot c\cdot d}\sum_{a}p_{a|b\cdot c\cdot d}\ln\left(p_{a|b\cdot c\cdot d}\right)\;,$$

where the sum of the insertion probabilities multiplied by their logarithmic functions is further summed up for all possible neighborhood states weighted by their respective neighborhood probabilities.

In combination with Equations (1) and (2), Equation (12) can only be expressed by cluster probabilities:

(13) $$S_{\mathrm{system}}=-k_{B}N\sum_{a}\sum_{b}\sum_{c}\sum_{d}p_{a\cdot b\cdot c\cdot d}\ln\left(\frac{p_{a\cdot b\cdot c\cdot d}}{\sum_{\tilde{a}\in a^{*}}p_{\tilde{a}\cdot b\cdot c\cdot d}}\right)\;.$$

### 2.3. Internal Energy of the System

To account for cooperative effects, the internal energy of the system is calculated as the average of the cluster energies, $e_{a\cdot b\cdot c\cdot d}$, weighted by the respective cluster probabilities. This energy represents both the internal energy of an ensemble of clusters and internal energy of the sequentially constructed lattice system:

(14) $$U_{\mathrm{cluster}}=U_{\mathrm{system}}=N\sum_{a}\sum_{b}\sum_{c}\sum_{d}p_{a\cdot b\cdot c\cdot d}\cdot e_{a\cdot b\cdot c\cdot d}\;.$$

The cluster energies, $e_{a\cdot b\cdot c\cdot d}$, are additively composed of the nearest-neighbor interactions *A–B*, *A–C*, and *A–D*, as can be seen in the right-hand part of Figure 1. Each of the three bonds contributes an energy according to the respective opposite surface segments. However, Equation (14) does not consider long-range interactions, such as those with the next-nearest neighbors. While in this paper more or less artificial interaction energies were used to perform Monte-Carlo simulations, for real molecules, force fields or quantum-mechanical calculations could be used to determine the cluster internal energies, for example.

**Figure 2:**
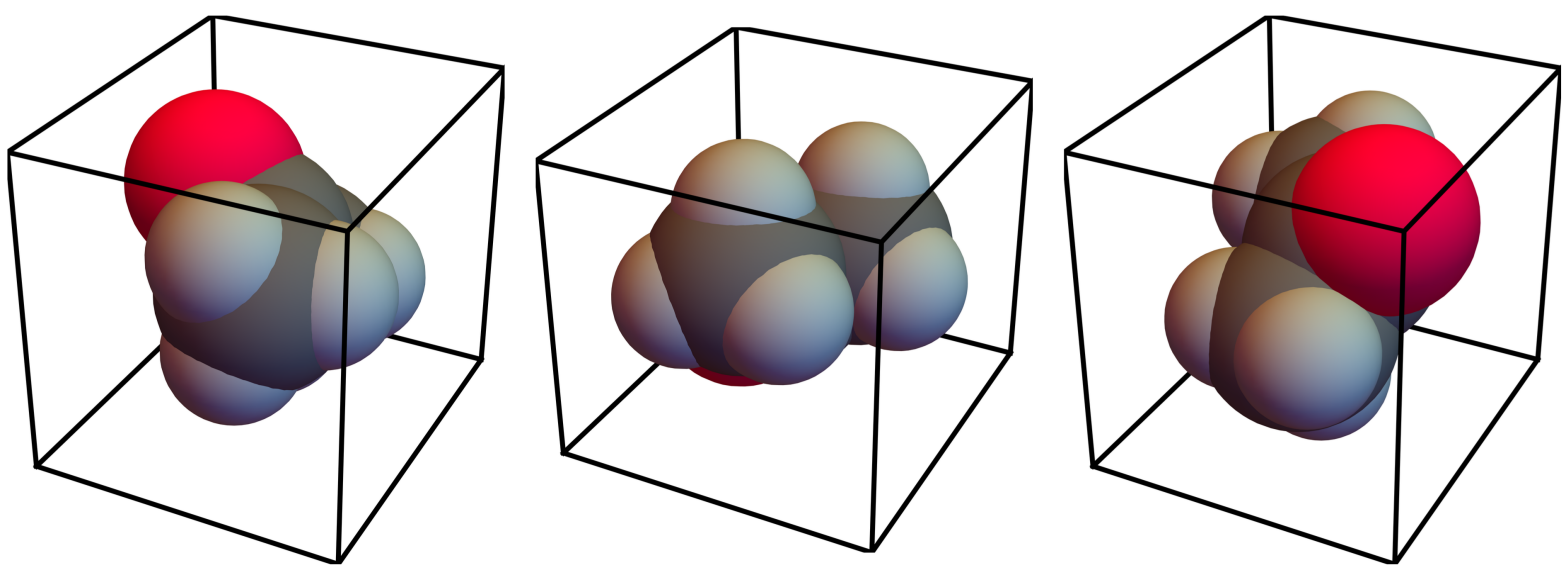
Three of the 24 possible orientations of an acetone molecule when linked to a die-like representation. The orientations are generated by rotating the die in 90 degree steps.

Figure 2 illustrates a possibility how real molecules could be linked to die-like representations, using an acetone molecule as an example. The link can be created by fixing the molecule’s orientation within a die. Consequently, the molecule can then only be in one of the 24 possible orientations, three of which are depicted in the figure. Clusters are then constructed by combining multiple molecules, which are limited to these 24 orientations, on a grid.

### 2.4. Helmholtz Free Energy of the System

A straightforward way to determine the cluster probabilities, $p_{a\cdot b\cdot c\cdot d}$, of the system in thermodynamic equilibrium would be to maximize the system’s entropy, as in Equation (13), considering the internal energy, Equation (14), as a constraint together with further constraints that are explained later in Section 2.5. However, in thermodynamic modeling, it is more common to minimize the Helmholtz free energy instead. This function, defined as A≡U−TS, results from a so-called Legendre transformation of the internal energy, where the entropy, *S*, is replaced by the temperature, *T*, while the extremal characteristics of the original function, *U*, are transferred to the variable space of the transformed function, *A* [50]. This results in the following equivalent options for extremalization:

(15) $$\begin{array}{ll} S=S(U,V,N_{i}) & S=max\;(U,V,N_{i})\\ & ⇕\\ U=U(S,V,N_{i}) & U=min\;(S,V,N_{i})\\ & ⇕\\ A=A(T,V,N_{i}) & A=min\;(T,V,N_{i}) \end{array}$$

Consequently, according to Equation (15), the equilibrium distribution of the cluster probabilities can be determined by minimizing the Helmholtz free energy, when considering the constraints to be explained in the following section.

### 2.5. Constraints Applied to Minimize the Helmholtz Free Energy

First, three merely mathematical conditions can be derived from the law of total probability. Second, the requirement for systemic isotropy, which ensures the uniformity of properties in all orientations, allows for clusters to be grouped into classes, whereby each member of a class can be assigned one and the same probability. Third, approximating the cluster probabilities by using probabilities of pairs of molecules further reduces the number of variables to a considerable extent. Finally, model-related symmetries provide additional constraints that contribute to the numerical solvability of the system.

#### 2.5.1. Mathematical Constraints

First, when applied over the entire cluster, the application of the law of total probability results in the condition

(16) $$\sum_{a}\sum_{b}\sum_{c}\sum_{d}p_{a\cdot b\cdot c\cdot d}=1\;.$$

Second, when applied over molecules of one type only, this law establishes a connection to molecular fractions, which are defined as

(17) $$x_{i}\equiv \frac{N_{i}}{N}$$

and can be interpreted as the global compositions of the system, which represent the known input variables for the model. When a single molecule is picked at random out of the lattice system, the probability that it is a component of type *i* must equal $x_{i}$. If we apply this logic to a cluster, this means that the marginal probability of the cluster is being equal to the global composition, yielding the two constraints

(18) $$\begin{array}{lll} \sum_{a\in a_{1}^{*}}\sum_{b}\sum_{c}\sum_{d}p_{a\cdot b\cdot c\cdot d}=p_{1} & = & x_{1}\;\mathrm{and} \end{array}$$

(19) $$\begin{array}{lll} \sum_{a\in a_{2}^{*}}\sum_{b}\sum_{c}\sum_{d}p_{a\cdot b\cdot c\cdot d}=p_{2} & = & x_{2}, \end{array}$$

where $a_{i}^{*}$ are subsets of *a* for the component of type *i*.

At this point, the three constraints Equation (16), Equation (18), and Equation (19), together with an objective function for extremalization, Equation (15), provide four equations. This is contrasted with the number of ${\left(2\times 24\right)}^{4}+1=$ 5,308,417 variables, accounting for two components (i.e., black or white dice), 24 possible rotations of a die, four possible positions of a die within the cluster, and one target property (i.e., the Helmholtz free energy). Obviously, further properties of the system must be formulated as constraints in order to decrease the gap between these two numbers.

#### 2.5.2. Constraints Reflecting Lattice Isotropy

**Figure 3:**
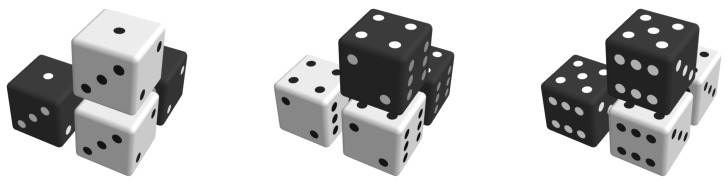
An illustration of the isotropic property of clusters: three clusters that can be considered as one cluster viewed from different angles; therefore, the three clusters can be assigned one and the same probability of occurrence.

To obey a principle of probability theory that has been given many different names, such as the ‘principle of insufficient reason’ by Bernoulli or ‘desideratum of consistency’ by Jaynes [51], clusters for which there is no reason that either should be more probable than the other should be assigned equal probabilities. One justification for applying this principle is the absence of any directional effects in the lattice that would allow one to distinguish between different observation directions of the desired bulk properties. While the lattice may have some local directional ordering, these orderings have no preferred direction in an ensemble of lattices and, therefore, the ensemble mean of the individual probabilities shows no directional preference. This means that the lattice is considered to be isotropic. Isotropy must also be required for clusters, in such a way that a cluster that is selected from the system is selected, regardless of the viewing perspective. For the four-molecule clusters considered in this work, there are three directions from which they can be viewed, which results in one and the same cluster shape. Figure 3 depicts an example of this. To conclude, in terms of the resulting probability distribution, the clusters also have to have an isotropic property.

Based on these considerations, several classes of clusters, which have the same probability of occurrence in the system, can be formed. It can be shown that this step results in $\frac{2}{3}\times {\left(2\times 24\right)}^{4}$ = 3,538,944 new conditions. The system of equations then comprises 3,538,948 conditions in total, while maintaining 5,308,417 variables, as explained before.

The constraints that have been discussed so far represent linear functions of the cluster probabilities, $p_{a\cdot b\cdot c\cdot d}$. However, preliminary tests conducted to minimize the Helmholtz free energy with respect to only the constraints discussed hitherto revealed that additional conditions of a different, essentially nonlinear nature were required, as explained in the following section.

#### 2.5.3. Constraints Derived from Cluster Construction

The number of independent variables can be further substantially reduced by approximating the cluster probabilities with a functional relationship for probabilities of pairs of molecules. This is achieved by constructing the clusters in a sequential manner, similar to the construction of the entire lattice.

For the lattice construction, a new molecule is inserted into an already present neighborhood of three other molecules. Thus, the construction of a cluster can be described by rearranging Equation (1) to

(20) $$p_{a\cdot b\cdot c\cdot d}=p_{b\cdot c\cdot d}\cdot p_{a|b\cdot c\cdot d}\;.$$

In this context, a useful mathematical consideration when dealing with conditional probabilities is the fact that a combined probability does not depend on the construction direction. Consequently, instead of inserting a molecule into position *A*, where the neighborhood at positions *B*, *C*, and *D* is already present, it is possible to start the construction of the cluster from the reverse viewpoint at position *A* and then fill the positions *B*, *C*, and *D*, depending on the molecule at position *A*:

(21) $$p_{a\cdot b\cdot c\cdot d}=p_{a}\cdot p_{b\cdot c\cdot d|a}\;.$$

**Figure 4:**
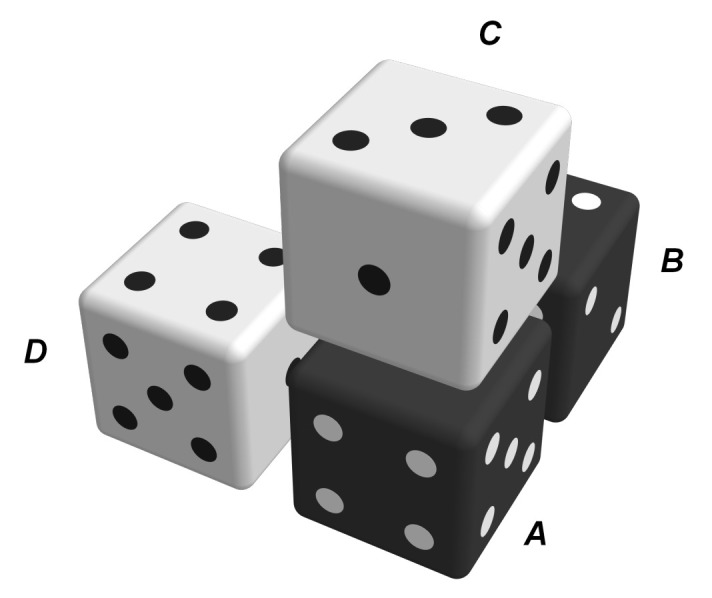
Example of a four-molecule cluster with labeled molecule positions.

Figure 4 illustrates the locations of positions *A*, *B*, *C*, and *D* within the cluster.

Now, if the insertions at *B*, *C*, and *D* are assumed to be only dependent upon *A* and not upon one another, Equation (21) can be split up into

(22) $$p_{a\cdot b\cdot c\cdot d}=p_{a}\cdot p_{b|a}\cdot p_{c|a}\cdot p_{d|a}\;,$$

which is analogous to a constraint originally that was proposed by Vinograd et al. [36] and that was extended in a previous work to three-dimensional lattices for pure components [41]. The interpretation of Equation (22) is that, first, the central molecule at position *A* is selected based on the global composition of the lattice; then, each of the neighbors is added to the cluster, with a probability of its respective component that is dependent on *a*. These probabilities are referred to later on as conditional pair probabilities.

The properties of this type of cluster construction and of the system in general simplify the calculations and that further reduce the number of variables that need to be determined by optimization. The isometric property of the system also manifests itself on the level of single molecules. Here, all the orientations of one component type are assigned one and the same probability, which can be related to the global composition, $x_{i}$, via

(23) $$\begin{array}{lll} p_{a} & = & x_{1}/24\;\forall a\in a_{1}^{*}\;,\;\mathrm{and} \end{array}$$

(24) $$\begin{array}{ccc} p_{a} & = & x_{2}/24\;\forall a\in a_{2}^{*}. \end{array}$$

#### 2.5.4. Model-Related Constraints

**Figure 5:**
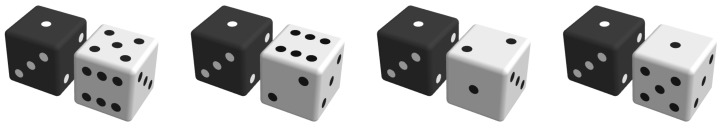
One of the approximations of the model is that, in line with Equation (22), molecule pairs (illustrated as dice) with the same contact sites, here 
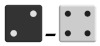
, are assumed to have the same probability of occurrence; the model does not distinguish between these configurations.

One of the approximations of the model is that, for four-molecule clusters where the same component types are located at identical positions, the only relevant distinction between them are the three pairs of contacting sites, cf. Figure 4. These determine, for instance, the internal energy of a specific cluster, cf. Equation (14). This property originates from the approximation given in Equation (22). Clusters that only differ in the location of sites that are not involved in the contacting pairs are assumed to have equal probabilities of occurring within the system. This is illustrated in Figure 5 on the basis of a pair of molecules.

### 2.6. Resulting System of Equations

The resulting system of equations, (25a–25g), comprises the target function (i.e., the Helmholtz free energy) to be minimized, Equation (25a), and the constraints, Equations (25b) through (25g). The temperature *T*, compositions $x_{i}$, and cluster internal energies $e_{a\cdot b\cdot c\cdot d}$ are input parameters of the model. The latter are additively composed of the nearest-neighbor interactions *A–B*, *A–C*, and *A–D* and they can be determined in practice from intermolecular force fields or quantum-mechanical calculations, for example.

(25a) $$\begin{array}{ll} A & =N\sum_{a}\sum_{b}\sum_{c}\sum_{d}p_{a\cdot b\cdot c\cdot d}\left(e_{a\cdot b\cdot c\cdot d}+k_{B}T\ln\frac{p_{a\cdot b\cdot c\cdot d}}{\sum_{\tilde{a}\in a^{*}}p_{\tilde{a}\cdot b\cdot c\cdot d}}\right)\overset{!}{=}\min \end{array}$$

(25b) $$\begin{array}{ll} 1 & =\sum_{a}\sum_{b}\sum_{c}\sum_{d}p_{a\cdot b\cdot c\cdot d} \end{array}$$

(25c) $$\begin{array}{ll} x_{1} & =\sum_{a\in a_{1}^{*}}\sum_{b}\sum_{c}\sum_{d}p_{a\cdot b\cdot c\cdot d} \end{array}$$

(25d) $$\begin{array}{ll} x_{2} & =\sum_{a\in a_{2}^{*}}\sum_{b}\sum_{c}\sum_{d}p_{a\cdot b\cdot c\cdot d} \end{array}$$

(25e) $$\begin{array}{ll} p_{a} & =x_{1}/24\;\forall a\in a_{1}^{*} \end{array}$$

(25f) $$\begin{array}{ll} p_{a} & =x_{2}/24\;\forall a\in a_{2}^{*} \end{array}$$

(25g) $$\begin{array}{ll} p_{a\cdot b\cdot c\cdot d} & =p_{a}\;p_{b|a}\;p_{c|a}\;p_{d|a} \end{array}$$

The probabilities both of the clusters, $p_{a\cdot b\cdot c\cdot d}$, and the pairs, $p_{b|a}$, $p_{c|a}$, and $p_{d|a}$, are the variables that need to be determined. In the most general case, where each site of both types of molecules has a different energetic interaction property, there is a total number of 5,310,703 equations and a total number of 5,310,769 variables, leaving 66 conditional pair probabilities as unknown variables to be determined over the course of the minimization.

The proof of the thermodynamic consistency of the model while using a Gibbs–Helmholtz equation is numerically outlined in the Supplementary Material.

## 3. Results

### 3.1. Random Mixing

For systems without cooperative effects, it is not possible to differentiate between the orientations of the components. Therefore, only the component type at each cluster position influences the equilibrium distribution of the probabilities. This case, where the molecules are modeled like uniform spheres rather than six-sided dice, connects this work to previous work [41]. The effects of such a gross simplification on the model are discussed for both pure components and two-component mixtures in the following section.

#### 3.1.1. Pure-Component Systems

A pure-component system without cooperative effects drastically reduces the complexity of the model’s system of equations, as only one component type and only one molecule orientation must be considered. As a result, each set of states for every cluster position, $a^{*}$, $b^{*}$, $c^{*}$, and $d^{*}$, only has one member. Recalling Equation (16), the sum over all cluster probabilities in this case reduces to the one possible probability, which then has to be unity. Consequently, the entropy, Equation (13), is zero, which is the expected value for this limiting case.

#### 3.1.2. Two-Component Systems

In two-component mixtures, it is necessary to differentiate between two component types per cluster position. Again, only one orientation per molecule can be modeled. Consequently, the sets $a^{*}$, $b^{*}$, $c^{*}$, and $d^{*}$ contain two states each. With no cooperative effects, the molecules are placed into the lattice at random. The insertion steps are independent of their respective neighborhood and, therefore, the conditional probabilities that are used to describe such insertion steps also lose their dependency upon their neighborhoods,

(26) $$\begin{array}{lll} p_{b|a} & = & p_{b},\\ p_{c|a} & = & p_{c},\mathrm{and}\\ p_{d|a} & = & p_{d}. \end{array}$$

Once these relations are inserted into Equation (22), the cluster probabilities can be directly calculated from the global composition by applying

(27) $$p_{a\cdot b\cdot c\cdot d}=p_{a}\;p_{b}\;p_{c}\;p_{d}.$$

Consequently, with these considerations, the entropy, Equation (13), is reduced to

(28) $$S_{\mathrm{system}}=-k_{B}N\left(p_{1}\ln\left(p_{1}\right)+p_{2}\ln\left(p_{2}\right)\right)\;,$$

where the two possible states for each molecule are indexed by 1 and 2. Thus, it is shown that the system of equations yields the expected result for the special case of random mixing [50].

### 3.2. Non-Random Mixing Considering Cooperative Effects

**Figure 6:**
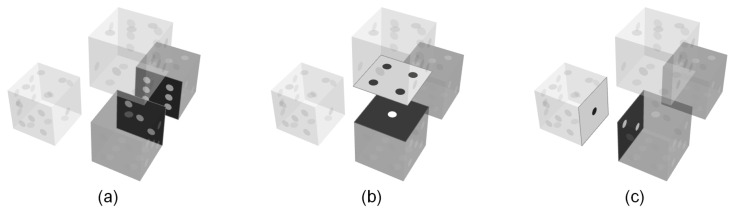
Highlighted contacting site pairs from the exemplary cluster given in Figure 4: (**a**) molecules *A–B*, (**b**) molecules *A–C*, and (**c**) molecules *A–D*.

Cooperative effects are considered in the model in the form of the internal energy of a cluster, $e_{a\cdot b\cdot c\cdot d}$, which is defined as the sum of the three pairwise interaction energies that occur within a four-molecule cluster. These pairwise interaction energies are defined by the touching faces inside the cluster. To use Figure 4 as an example, $e_{a\cdot b\cdot c\cdot d}$ would be additively composed of the nearest-neighbor interactions *A–B*, *A–C*, and *A–D*. Figure 6 highlights these three interaction pairs.

The particular pairwise interaction energies according to the touching faces of this example are: (a) ε_
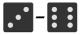
_, (b) ε_
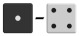
_, and (c) ε_
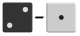
_. The energy of the whole system is then calculated by Equation (14).

In the most general case, each of the six faces of a component can be assigned an individual energetic property. In a two-component system, this results in a total of 12 different sites, which can form 78 distinct pairwise interaction energies. The number 78 results from the selection of two sites from the set of 12 without considering the order; to put it in terms of combinatorics, 78 is the number of subpopulations of size 2 without ordering.

However, in the following sections, the modeling approach is applied to two mixtures of simple pseudo-molecules with a reduced number of interaction properties. Both for ease of understanding and to highlight the geometric capabilities of the approach, i.e., the distinction between isomers, multiple faces of a die are assigned the same properties.

In the course of that, three interaction classes are introduced for the energetic interactions between each pair of faces of the contacting dice. These interaction classes are indicated with the numbers 0, 1, and 2, and they characterize pairwise interaction energies, $\varepsilon_{ij}$. Here, the class of the first site involved in the contact is indicated by *i*, and the class of the second one, by *j*. Whenever either *i* or *j* is of class 0, no interaction occurs, resulting in an interaction energy of zero. Otherwise, when interactions *i* and *j* are different (1–2 or 2–1), an attraction results, and interactions where *i* and *j* are the same (1–1 or 2–2) result in repulsion. The specific values for the interaction energies used in this work are given in Table 1 in a dimensionless form, $\frac{\varepsilon_{ij}}{Nk_{B}T}$.

**Figure 7:**
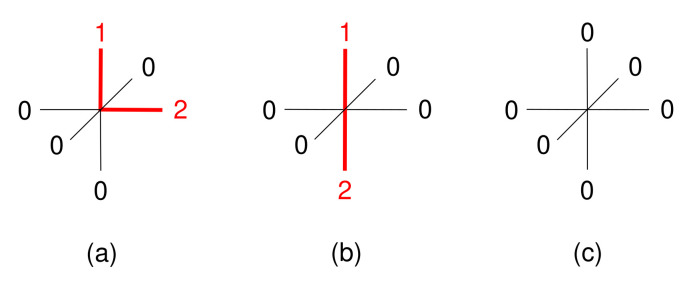
Site occupancies of the three molecule types used in this paper: (**a**) Angled, (**b**) Stretched, and (**c**) Inert.

Based on the three interaction classes introduced above, three different molecule types are considered in the following, i.e., ‘Angled’, ‘Stretched’, and ‘Inert’ molecules. Figure 7 illustrates these in the form of their site occupancies.

Both Angled and Stretched molecules are made up of one die face being of class 1, one die face being of class 2, and the remaining faces being of class 0. For the Angled molecules, classes 1 and 2 are located next to each other, so that the faces share a common edge. Stretched molecules, on the other hand, have classes 1 and 2 located on opposite sides of the dice. Because Angled and Stretched molecules only differ in terms of their relative charge positions, they represent isomers. The names Angled and Stretched are chosen to indicate these relative positions of the non-zero die faces and they do not infer any further properties of the molecules. Finally, Inert molecules have a uniform surface that consists only of class 0.

Overall, with these three molecule types, it is possible to reproduce mixtures of isomeric molecules. As the distinction between isomers still represents a challenge to well-established quasichemical approaches, it is obvious that the extent to which the model proposed in this paper can be used to distinguish between isomeric configurations must be explored, in order to assess its future development potential.

The system of Equations (25a–25g) was solved numerically by means of an interior-point method [52], which proved to be more suitable than the NSGA-II genetic algorithm [53] in most cases and the Simplex direct search method [54], which was also used for comparison. The model results were compared to those from Monte-Carlo simulations, as shown in the following section.

#### 3.2.1. Comparison with Monte-Carlo Simulations

The Monte-Carlo data were obtained from simulations of six-sided dice in a simple cubic lattice with an edge length of 30 sites, using the classical Metropolis algorithm as a basis [55,56,57,58]. For given temperatures, compositions and interchange energies, the simulations yielded the internal energy of the lattice system in equilibrium. From the latter, the Helmholtz free energy was numerically derived with a Gibbs–Helmholtz integration [57]. Supplementary Material summarizes details of the simulations along with their results.

One advantage of the model over Monte-Carlo simulations is that the entropy and Helmholtz free energy can be directly determined for a given temperature and composition. To achieve the same with Monte-Carlo simulations, the determination of a temperature series around the desired point would be required as a prerequisite for a Gibbs–Helmholtz integration. In terms of computation time for the specific example cases presented in this work, a single Monte-Carlo point required 34 min. CPU time on average, while the model evaluation required an average of 29 s CPU time.

For the sake of clarity in the plots below, the so-called interchange energy [3], $\omega_{ij}$, was introduced, which is defined as

(29) $$\omega_{ij}\equiv \varepsilon_{ij}+\varepsilon_{ji}-\varepsilon_{ii}-\varepsilon_{jj}\;,$$

combining the interaction energies into one quantity. As in the case of interaction energies, a negative value of $\omega_{ij}$ characterizes the overall attraction, and a positive value of $\omega_{ij}$ characterizes the overall repulsion between the molecules.

#### 3.2.2. Mixtures of Angled + Inert Molecules

**Figure 8:**
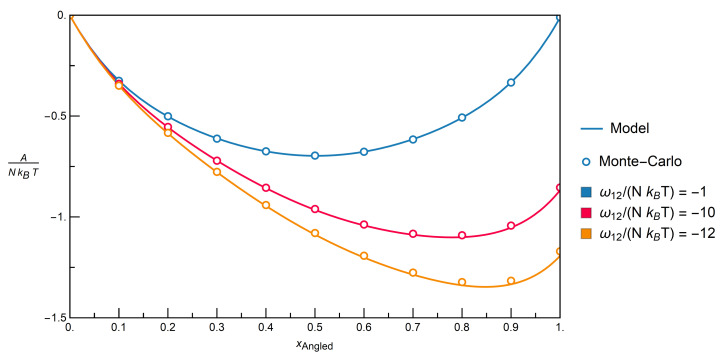
Comparison of the model with Monte-Carlo simulation data via the Helmholtz free energy over the molar fraction for the mixture Angled + Inert for constant dimensionless interchange energies resp. constant temperatures; −1 corresponds to a slight attraction resp. high temperature, and −12, to a strong attraction resp. low temperature.

The first system that is examined contains a mixture of Angled and Inert molecules. Figure 8 gives the results for the system in terms of the dimensionless Helmholtz free energy, $A/(Nk_{B}T)$, as a function of the molar fraction of Angled molecules for constant dimensionless interchange energies, $\omega_{ij}/\left(Nk_{B}T\right)$.

Because the interchange energy itself is constant in a specific mixture, the different dimensionless interchange energies can be interpreted as different temperatures. The topmost curve is for a dimensionless interchange energy of −1, which is close to random mixing, that would have a value of 0. This corresponds to either weakly attractive interactions or a system at high temperatures. The other curves feature decreasing temperatures and/or a stronger overall attraction between the molecules. Although deviations between the results obtained while using the model and Monte-Carlo data tend to increase as the negative dimensionless interchange energies increase, it is evident that the model can be used to accurately describe the Monte Carlo data.

#### 3.2.3. Mixtures of Stretched + Inert Molecules

**Figure 9:**
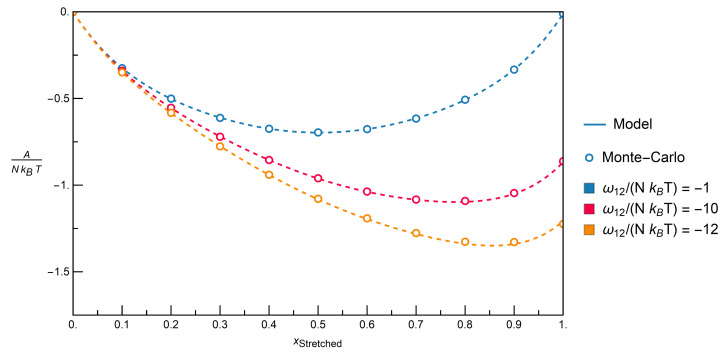
Comparison of the model with Monte-Carlo simulation data via the Helmholtz free energy over the molar fraction for the mixture Stretched + Inert for constant dimensionless interchange energies resp. constant temperatures; −1 corresponds to a slight attraction resp. high temperature, and −12, to a strong attraction resp. low temperature.

Figure 9 provides the results for mixtures of Stretched and Inert molecules, as in the previous system. Again, an excellent agreement between the results from the model and Monte-Carlo data is seen.

#### 3.2.4. Distinction between Isomers

**Figure 10:**
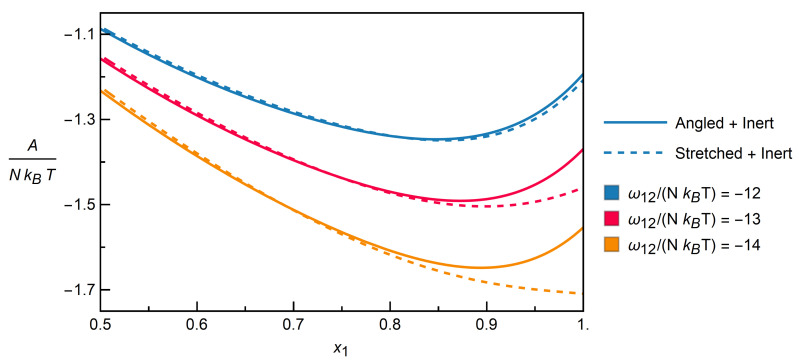
Dimensionless Helmholtz free energy as calculated by the model over the molar fraction of the respective non-inert component, for the mixtures Angled + Inert and Stretched + Inert, at strongly negative dimensionless interchange energies resp. low temperatures.

Model results for both aforementioned mixtures are plotted together in Figure 10 to explore the extent to which the model can be applied to distinguish between isomers.

In order to highlight the differences, the plot zooms in on the parts that show both high molar fractions of the respective interacting component (Angled or Stretched) and stronger attraction, i.e., more negative dimensionless interchange energies.

The rapidly growing differences between the mixtures start to be pronounced at values for $\omega_{12}/\left(Nk_{B}T\right)$ of around −12. These can be explained by the fact that Stretched molecules build up a local ordering of molecules in the form of chains, whereby interactions of class 1 prefer contacts of class 2 due to attraction forces, cf. Table 1. In contrast, Angled molecules show no sign of a similar local ordering, at least at the investigated interchange energies.

To conclude, because the model can be used to accurately describe the individual mixtures of Angled + Inert and Stretched + Inert components for extremely strong overall attractions between the molecules, resp. down to low temperatures, it can be consequently used to distinguish between isomeric configurations.

## 4. Conclusions and Outlook

The discrete modeling approach that is described in this paper, encompassing discrete states of molecules within their neighborhoods of interaction, shows its strenghts when combined with clusters. Even the modestly sized four-molecule cluster, where only three bonds effectively influence its internal energy, yields a model with the intrinsic ability to distinguish between isomers. This represents a considerable improvement over the broadly used quasichemical approximation. This charming feature, which results from a sequential construction of both lattices and clusters with conditional probabilities, endows the model with geometric information that many other approaches do not include. In order to further explore the potential of the approach, it should be applied to real molecules and, for example, a force field or quantum-mechanical calculations should be used in order to determine the cluster internal energies. Although the dice model is inherently confined to mixtures of equally-sized molecules, and long-chained molecules are avoided which would result in size-asymmetric mixtures, any such further development could help to further improve the approach and develop an excess Gibbs energy model for use with condensed phase mixtures. 

## Figures and Tables

**Table 1:** Overview of interaction energies in a dimensionless form for pairs of contacting dice faces.

Contact Pairs (*i-j*)	$\frac{\varepsilon_{\mathrm{ij}}}{Nk_{B}T}$	Interaction
1-1, 2-2	1200	repulsion
1-2, 2-1	−1200	attraction
0-any, any-0	0	inert

## Supplementary Materials

The following are available online at https://www.mdpi.com/1099-4300/22/10/1111/s1, proof of the thermodynamic consistency of the model using a Gibbs-Helmholtz equation on a numerical basis, Table S1: Results from Monte-Carlo simulations of the two-component system {Stretched+Inert} at different temperatures and molar fractions of the Stretched component, Table S2: Results from Monte-Carlo simulations of the two-component system {Angled+Inert} at different temperatures and molar fractions of the Angled component.

## Abbreviations

Abbreviations in the text:

bQCE	binary quantum cluster equilibrium theory
COSMO	conductor-like screening model
COSMO-RS	conductor-like screening model for real solvents
COSMOSPACE	COSMO surface-pair activity coefficient equation
CVM	cluster variation method
MCC	multiscale cell correlation method
MD	molecular dynamics
UNIFAC	universal quasichemical functional group activity coefficients
VSC	Vienna Scientific Cluster
Nomenclature in formulae:	
$a^{*}$, $b^{*}$, $c^{*}$, $d^{*}$	set of all possible states that the molecules at positions *A*, *B*, *C*,
	and *D* in the cluster can reside in
a,b,c,d	states of the molecules located at positions *A*, *B*, *C*, and *D*
*A*	Helmholtz free energy
$e_{a\cdot b\cdot c\cdot d}$	internal energy of a cluster
$g^{E}$	excess Gibbs-energy
$H_{\mathrm{system}}$	Shannon information of the system
$k_{B}$	Boltzmann’s constant
m,n	numbers of possible state for each of the two probability distributions p,q
*N*	number of molecules
$p_{b\cdot c\cdot d}$	probability to find the three nearest neighbors for the central
	molecule in a particular state
$p_{a\cdot b\cdot c\cdot d}$	probability of an entire cluster to be in a particular state
$p_{b|a},p_{c|a},p_{d|a}$	probabilities of inserting molecules of states b,c, resp.
	*d* into an existing neighborhood of state *a*
$p_{a|b\cdot c\cdot d}$	probability of inserting a molecule of state *a* into an existing neighborhood
	of states *b*, *c*, and *d*
*P*	probability function
p,q	probability distributions
$p_{i}^{\mathrm{system}}$	probability of finding a system in state *i*
$S_{\mathrm{cluster}}$	thermodynamic entropy of an ensemble of clusters
$S_{\mathrm{system}}$	thermodynamic entropy of the system
*T*	temperature
$U_{\mathrm{cluster}}$	internal energy of an ensemble of clusters
$U_{\mathrm{system}}$	internal energy of the system
*V*	volume
$x_{i}$	molecular fraction or molar fraction of component *i*
Y,Z	random variables
$y_{i},z_{i}$	state *i* of the random variables *Y*, and *Z*
$\varepsilon_{ij}$	interaction energy for the contact pair *i-j*
$\omega_{ij}$	interchange energy for the contact pair *i-j*
