# Supplementary material for: Cluster-Based Thermodynamics of Interacting Dice in a Lattice
Source: Entropy (Basel). 2020 Oct 1;22(10):1111. doi: 10.3390/e22101111 (PMC7597232; doi:10.3390/e22101111)
Supplement: Supplementary file 1 [file entropy-22-01111-s001.zip › Supplementary Files/DicePaperSupplementary.pdf]

Supplementary Materials: Cluster-based Entropy of Interacting Dice in a Lattice

Christoph Mayer , and Thomas Wallek

1. Thermodynamic Consistency Check

The thermodynamic consistency of the model developed in this work was proven using the Gibbs-Helmholtz equation

$$\left(\frac{\partial \frac{A}{T}}{\partial T}\right)_{V,x_i} = -\frac{U}{T^2}, \tag{1}$$

where A denotes the Helmholtz free energy, T, the temperature, U, the internal energy, V, the volume, and x_i, the global molar fractions.

Because the model is not formulated in terms of an explicit solution, the consistency check was performed numerically for constant values of the global composition and interaction energies. Because of the shape of A/T over $1/T$, a 9th degree polynomial was fitted to it and used to determine the derivative at the investigated temperatures. For the model developed, Equation 1 is fulfilled to a degree that approaches computational accuracy.

2. Monte-Carlo Simulations

The Monte Carlo data for the internal energy were directly obtained from simulations of six-sided dice in a simple cubic lattice with an edge length of 30 sites, using the classical Metropolis algorithm as a basis.

The calculations were started from a random configuration of the dice in the lattice and were conducted with 100 initial loops for equilibration of the system, followed by 100 loops for evaluation in terms of averaging of the internal energy. Each of these equilibration and evaluation loops involved 10^7 Monte-Carlo steps with one dice exchange and one dice rotation in each case.

Results of the simulations at various temperatures and compositions are summarized in the following Tables S1 and S2.

Table S1. Results from Monte-Carlo simulations: $U/(N\,R)$ [K] of the two-component system {Stretched + Inert} at different temperatures, T [K], and molar fractions of the Stretched component, x_s [mol/mol]. U . . . internal energy [J]; N . . . mole number [mol]; R . . . universal gas constant [J $K^{-1}mol^{-1}$].

T [K]	$x_s = 0.0$	$x_s = 0.1$	$x_s = 0.2$	$x_s = 0.3$	$x_s = 0.4$	$x_s = 0.5$
10000.00	0.0E+0	-4.8124553E-1	-1.9229941E+0	-4.3224371E+0	-7.6894740E+0	-1.1988729E+1
4800.00	0.0E+0	-1.0091537E+0	-4.0278115E+0	-9.0419678E+0	-1.6042502E+1	-2.5023262E+1
2400.00	0.0E+0	-2.0671673E+0	-8.1978515E+0	-1.8315509E+1	-3.2324864E+1	-5.0157261E+1
1600.00	0.0E+0	-3.2234842E+0	-1.2676659E+1	-2.8047006E+1	-4.9074349E+1	-7.5540962E+1
1200.00	0.0E+0	-4.5405850E+0	-1.7585691E+1	-3.8414929E+1	-6.6450481E+1	-1.0123443E+2
960.00	0.0E+0	-6.0586812E+0	-2.3056500E+1	-4.9553642E+1	-8.4550414E+1	-1.2721320E+2
800.00	0.0E+0	-7.8361053E+0	-2.9158569E+1	-6.1589506E+1	-1.0340705E+2	-1.5350974E+2
685.71	0.0E+0	-9.9150044E+0	-3.5946818E+1	-7.4400482E+1	-1.2295184E+2	-1.8001234E+2
600.00	0.0E+0	-1.2349214E+1	-4.3508900E+1	-8.8065321E+1	-1.4306719E+2	-2.0656985E+2
533.33	0.0E+0	-1.5163170E+1	-5.1746141E+1	-1.0241005E+2	-1.6353775E+2	-2.3297351E+2
480.00	0.0E+0	-1.8400066E+1	-6.0645767E+1	-1.1727954E+2	-1.8421446E+2	-2.5919829E+2
436.36	0.0E+0	-2.2032006E+1	-7.0037215E+1	-1.3251438E+2	-2.0485431E+2	-2.8482044E+2
400.00	0.0E+0	-2.6030320E+1	-7.9857571E+1	-1.4789968E+2	-2.2524349E+2	-3.0991634E+2
369.23	0.0E+0	-3.0419539E+1	-8.9959923E+1	-1.6318474E+2	-2.4517325E+2	-3.3406317E+2
342.86	0.0E+0	-3.5060675E+1	-1.0016553E+2	-1.7823194E+2	-2.6451058E+2	-3.5741546E+2
320.00	0.0E+0	-3.9985683E+1	-1.1035420E+2	-1.9290988E+2	-2.8314567E+2	-3.7982601E+2
300.00	0.0E+0	-4.4956457E+1	-1.2042472E+2	-2.0705038E+2	-3.0092952E+2	-4.0123558E+2
282.35	0.0E+0	-5.0109084E+1	-1.3008382E+2	-2.2045482E+2	-3.1778115E+2	-4.2213797E+2
266.67	0.0E+0	-5.5246146E+1	-1.3949416E+2	-2.3330524E+2	-3.3365339E+2	-4.4240255E+2
252.63	0.0E+0	-6.0334554E+1	-1.4844488E+2	-2.4545580E+2	-3.4892076E+2	-4.6829318E+2
240.00	0.0E+0	-6.5137133E+1	-1.5681335E+2	-2.5666473E+2	-3.6353921E+2	-4.9542307E+2
228.57	0.0E+0	-6.9835367E+1	-1.6472105E+2	-2.6735092E+2	-3.7906991E+2	-5.1483955E+2
218.18	0.0E+0	-7.4350388E+1	-1.7212174E+2	-2.7746092E+2	-3.9810106E+2	-5.3000002E+2
208.70	0.0E+0	-7.8510926E+1	-1.7895136E+2	-2.8710098E+2	-4.1328375E+2	-5.4218318E+2
200.00	0.0E+0	-8.2514436E+1	-1.8581839E+2	-2.9787387E+2	-4.2558403E+2	-5.5232766E+2
192.00	0.0E+0	-8.6217744E+1	-1.9156959E+2	-3.0957971E+2	-4.3552023E+2	-5.6057851E+2

T [K]	$x_s = 0.6$	$x_s = 0.7$	$x_s = 0.8$	$x_s = 0.9$	$x_s = 1.0$
10000.00	-1.7279782E+1	-2.3496580E+1	-3.0708036E+1	-3.8834096E+1	-4.7930488E+1
4800.00	-3.5975482E+1	-4.8881773E+1	-6.3730828E+1	-8.0573837E+1	-9.9311888E+1
2400.00	-7.1755073E+1	-9.7085508E+1	-1.2603897E+2	-1.5863045E+2	-1.9475273E+2
1600.00	-1.0726090E+2	-1.4401770E+2	-1.8577353E+2	-2.3230998E+2	-2.8360025E+2
1200.00	-1.4233334E+2	-1.8941891E+2	-2.4235102E+2	-3.0074368E+2	-3.6458235E+2
960.00	-1.7690781E+2	-2.3316146E+2	-2.9558558E+2	-3.6389528E+2	-4.3799909E+2
800.00	-2.1102648E+2	-2.7521164E+2	-3.4572731E+2	-4.2227410E+2	-5.0485852E+2
685.71	-2.4446868E+2	-3.1572406E+2	-3.9324678E+2	-4.7687826E+2	-5.6679844E+2
600.00	-2.7738137E+2	-3.5478442E+2	-4.3845933E+2	-5.2850113E+2	-6.2567445E+2
533.33	-3.0955667E+2	-3.9247095E+2	-4.8182803E+2	-5.7811994E+2	-6.8393255E+2
480.00	-3.4086386E+2	-4.2890903E+2	-5.2377124E+2	-6.2724818E+2	-7.4505051E+2
436.36	-3.7135394E+2	-4.6428875E+2	-5.6495876E+2	-6.7761268E+2	-8.3678487E+2
400.00	-4.0083222E+2	-4.9869383E+2	-6.0628082E+2	-7.3743531E+2	-1.1370793E+3
369.23	-4.2941656E+2	-5.3257016E+2	-6.5061948E+2	-9.0345172E+2	-1.1729153E+3
342.86	-4.5702211E+2	-5.6680604E+2	-7.4362866E+2	-9.5999287E+2	-1.1866332E+3
320.00	-4.8406170E+2	-6.1027625E+2	-8.0653671E+2	-9.8928472E+2	-1.1927078E+3
300.00	-5.1120455E+2	-6.7270301E+2	-8.4136037E+2	-1.0075162E+3	-1.1960493E+3
282.35	-5.4990040E+2	-7.1044899E+2	-8.6441014E+2	-1.0205373E+3	-1.1977469E+3
266.67	-5.8739255E+2	-7.3504171E+2	-8.8113792E+2	-1.0300989E+3	-1.1986829E+3
252.63	-6.1275707E+2	-7.5350543E+2	-8.9390670E+2	-1.0378018E+3	-1.1992158E+3
240.00	-6.3231645E+2	-7.6813161E+2	-9.0421254E+2	-1.0439630E+3	-1.1995411E+3
228.57	-6.4752989E+2	-7.7958800E+2	-9.1290141E+2	-1.0493076E+3	-1.1997218E+3
218.18	-6.5963972E+2	-7.8927841E+2	-9.2006624E+2	-1.0535846E+3	-1.1998321E+3
208.70	-6.6973737E+2	-7.9741725E+2	-9.2650692E+2	-1.0574239E+3	-1.1999021E+3
200.00	-6.7781258E+2	-8.0459155E+2	-9.3143262E+2	-1.0608103E+3	-1.1999388E+3
192.00	-6.8535716E+2	-8.1068448E+2	-9.3635134E+2	-1.0638476E+3	-1.1999637E+3

Table S2. Results from Monte-Carlo simulations: $U/(N\,R)$ [K] of the two-component system $\{$Angled + Inert$\}$ at different temperatures, T [K], and molar fractions of the Angled component, x_a [mol/mol]. U ... internal energy [J]; N ... mole number [mol]; R ... universal gas constant [J $K^{-1}mol^{-1}$].

T [K]	$x_a = 0.0$	$x_a = 0.1$	$x_a = 0.2$	$x_a = 0.3$	$x_a = 0.4$	$x_a = 0.5$
10000.00	0.0E+0	-4.8096584E-1	-1.9245412E+0	-4.3230716E+0	-7.6798153E+0	-1.2003834E+1
4800.00	0.0E+0	-1.0073350E+0	-4.0271040E+0	-9.0446464E+0	-1.6031579E+1	-2.5026460E+1
2400.00	0.0E+0	-2.0664264E+0	-8.2003864E+0	-1.8325987E+1	-3.2328267E+1	-5.0160007E+1
1600.00	0.0E+0	-3.2308206E+0	-1.2675476E+1	-2.8049164E+1	-4.9074446E+1	-7.5586775E+1
1200.00	0.0E+0	-4.5360463E+0	-1.7591021E+1	-3.8432636E+1	-6.6488341E+1	-1.0126284E+2
960.00	0.0E+0	-6.0572919E+0	-2.3054726E+1	-4.9588445E+1	-8.4623583E+1	-1.2731131E+2
800.00	0.0E+0	-7.8280291E+0	-2.9174906E+1	-6.1604548E+1	-1.0350135E+2	-1.5368644E+2
685.71	0.0E+0	-9.9223942E+0	-3.5998359E+1	-7.4509822E+1	-1.2311520E+2	-1.8025607E+2
600.00	0.0E+0	-1.2356795E+1	-4.3562016E+1	-8.8231690E+1	-1.4331622E+2	-2.0694464E+2
533.33	0.0E+0	-1.5187660E+1	-5.1848379E+1	-1.0266768E+2	-1.6393245E+2	-2.3351190E+2
480.00	0.0E+0	-1.8417241E+1	-6.0768126E+1	-1.1767434E+2	-1.8473514E+2	-2.5981073E+2
436.36	0.0E+0	-2.2073274E+1	-7.0356586E+1	-1.3305894E+2	-2.0562161E+2	-2.8556499E+2
400.00	0.0E+0	-2.6152193E+1	-8.0314274E+1	-1.4864216E+2	-2.2623073E+2	-3.1064852E+2
369.23	0.0E+0	-3.0604153E+1	-9.0638590E+1	-1.6424767E+2	-2.4640755E+2	-3.3479819E+2
342.86	0.0E+0	-3.5454657E+1	-1.0113747E+2	-1.7964108E+2	-2.6599402E+2	-3.5801459E+2
320.00	0.0E+0	-4.0523154E+1	-1.1167208E+2	-1.9465448E+2	-2.8474663E+2	-3.7985472E+2
300.00	0.0E+0	-4.5822850E+1	-1.2208521E+2	-2.0908681E+2	-3.0255917E+2	-4.0057695E+2
282.35	0.0E+0	-5.1315212E+1	-1.3225006E+2	-2.2292510E+2	-3.1941831E+2	-4.1995320E+2
266.67	0.0E+0	-5.6798561E+1	-1.4207948E+2	-2.3613539E+2	-3.3511374E+2	-4.3786310E+2
252.63	0.0E+0	-6.2326912E+1	-1.5153404E+2	-2.4836999E+2	-3.4967837E+2	-4.5432809E+2
240.00	0.0E+0	-6.7652469E+1	-1.6029215E+2	-2.5976409E+2	-3.6328087E+2	-4.6962397E+2
228.57	0.0E+0	-7.2882743E+1	-1.6877290E+2	-2.7036429E+2	-3.7562148E+2	-4.8353367E+2
218.18	0.0E+0	-7.7888127E+1	-1.7642858E+2	-2.8002154E+2	-3.8688751E+2	-4.9606668E+2
208.70	0.0E+0	-8.2579714E+1	-1.8348493E+2	-2.8881444E+2	-3.9711914E+2	-5.0745679E+2
200.00	0.0E+0	-8.7009598E+1	-1.9003671E+2	-2.9688014E+2	-4.0640282E+2	-5.1781551E+2
192.00	0.0E+0	-9.1060559E+1	-1.9585411E+2	-3.0412506E+2	-4.1478722E+2	-5.2694283E+2

T [K]	$x_a = 0.6$	$x_a = 0.7$	$x_a = 0.8$	$x_a = 0.9$	$x_a = 1.0$
10000.00	-1.7283063E+1	-2.3511228E+1	-3.0681396E+1	-3.8832744E+1	-4.7929638E+1
4800.00	-3.5962233E+1	-4.8888730E+1	-6.3773645E+1	-8.0541468E+1	-9.9321828E+1
2400.00	-7.1772349E+1	-9.7122197E+1	-1.2606269E+2	-1.5867412E+2	-1.9482657E+2
1600.00	-1.0730735E+2	-1.4411506E+2	-1.8585431E+2	-2.3245866E+2	-2.8377507E+2
1200.00	-1.4240525E+2	-1.8955286E+2	-2.4249686E+2	-3.0099906E+2	-3.6482775E+2
960.00	-1.7706271E+2	-2.3339421E+2	-2.9584065E+2	-3.6415826E+2	-4.3804226E+2
800.00	-2.1126935E+2	-2.7550287E+2	-3.4600653E+2	-4.2228179E+2	-5.0410516E+2
685.71	-2.4482868E+2	-3.1605266E+2	-3.9326728E+2	-4.7608498E+2	-5.6403561E+2
600.00	-2.7777823E+2	-3.5499704E+2	-4.3790963E+2	-5.2601910E+2	-6.1882046E+2
533.33	-3.0997410E+2	-3.9239288E+2	-4.8006156E+2	-5.7252243E+2	-6.6936523E+2
480.00	-3.4127785E+2	-4.2819657E+2	-5.1994434E+2	-6.1606051E+2	-7.1623898E+2
436.36	-3.7149611E+2	-4.6239661E+2	-5.5766216E+2	-6.5679721E+2	-7.5952712E+2
400.00	-4.0054632E+2	-4.9490029E+2	-5.9308402E+2	-6.9490371E+2	-7.9994272E+2
369.23	-4.2822248E+2	-5.2555077E+2	-6.2653343E+2	-7.3051219E+2	-8.3743453E+2
342.86	-4.5438271E+2	-5.5446212E+2	-6.5762123E+2	-7.6360316E+2	-8.7224855E+2
320.00	-4.7910454E+2	-5.8141693E+2	-6.8662301E+2	-7.9424539E+2	-9.0429523E+2
300.00	-5.0206739E+2	-6.0660310E+2	-7.1341246E+2	-8.2255567E+2	-9.3389175E+2
282.35	-5.2357598E+2	-6.2979666E+2	-7.3807449E+2	-8.4859437E+2	-9.6114700E+2
266.67	-5.4331637E+2	-6.5110370E+2	-7.6090550E+2	-8.7249004E+2	-9.8584475E+2
252.63	-5.6155171E+2	-6.7078952E+2	-7.8169265E+2	-8.9424508E+2	-1.0085343E+3
240.00	-5.7817739E+2	-6.8863197E+2	-8.0064763E+2	-9.1401345E+2	-1.0291283E+3
228.57	-5.9330274E+2	-7.0481140E+2	-8.1773758E+2	-9.3181009E+2	-1.0475568E+3
218.18	-6.0699167E+2	-7.1954260E+2	-8.3320242E+2	-9.4799363E+2	-1.0644155E+3
208.70	-6.1930831E+2	-7.3282061E+2	-8.4720934E+2	-9.6257124E+2	-1.0794245E+3
200.00	-6.3070123E+2	-7.4458040E+2	-8.5958446E+2	-9.7554191E+2	-1.0930353E+3
192.00	-6.4070710E+2	-7.5543351E+2	-8.7086758E+2	-9.8737301E+2	-1.1051913E+3
