# Supplementary material for: Cluster-Based Thermodynamics of Interacting Dice in a Lattice
Source: Entropy (Basel). 2020 Oct 1;22(10):1111. doi: 10.3390/e22101111 (PMC7597232; doi:10.3390/e22101111)
Supplement: Supplementary file 1 [file entropy-22-01111-s001.zip › Supplementary Files/Literature/Akkermans 2008 Mesoscale Model Parameters from Molecular Cluster Calculations.pdf]

AIP | The Journal of Chemical Physics

Mesoscale model parameters from molecular cluster calculations

Reinier L. C. Akkermans

Citation: The Journal of Chemical Physics **128**, 244904 (2008); doi: 10.1063/1.2943211
View online: http://dx.doi.org/10.1063/1.2943211
View Table of Contents: http://scitation.aip.org/content/aip/journal/jcp/128/24?ver=pdfcov
Published by the AIP Publishing

Articles you may be interested in
Efficient yet accurate approximations for ab initio calculations of alcohol cluster thermochemistry
J. Chem. Phys. **143**, 214306 (2015); 10.1063/1.4936406

Combining ab initio quantum mechanics with a dipole-field model to describe acid dissociation reactions in water: First-principles free energy and entropy calculations
J. Chem. Phys. **132**, 074112 (2010); 10.1063/1.3317398

Insights into photodissociation dynamics of acetaldehyde from ab initio calculations and molecular dynamics simulations
J. Chem. Phys. **131**, 054306 (2009); 10.1063/1.3196176

Polarizability of molecular clusters as calculated by a dipole interaction model
J. Chem. Phys. **116**, 4001 (2002); 10.1063/1.1433747

Thermochemical and kinetic parameters for hydrogen bonded clusters, derived from avalanche condensation flux measurements
J. Chem. Phys. **112**, 10192 (2000); 10.1063/1.481660

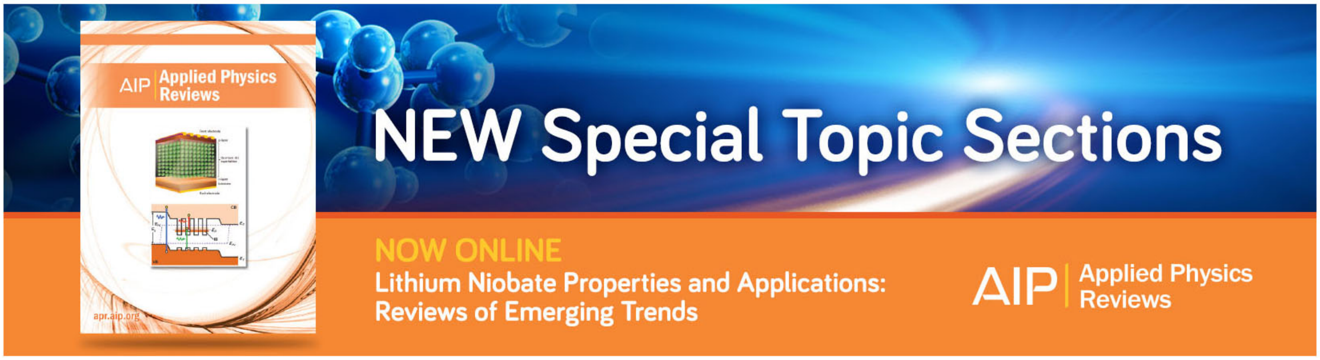

Reuse of AIP Publishing content is subject to the terms: https://publishing.aip.org/authors/rights-and-permissions. Downloaded to IP: 129.27.168.41 On: Sun, 04 Sep 2016 07:33:05

THE JOURNAL OF CHEMICAL PHYSICS **128**, 244904 (2008)

Mesoscale model parameters from molecular cluster calculations

Reinier L. C. Akkermans[a)]
Accelrys Ltd., 334 Cambridge Science Park, Cambridge CB4 0WN, United Kingdom

(Received 11 April 2008; accepted 19 May 2008; published online 25 June 2008)

We present an efficient, systematic, and universal method to estimate the interaction parameters used in mesoscale simulation methods such as dissipative particle dynamics and self-consistent field methods from molecular cluster calculations. The method is based on a generalized Flory–Huggins model in which molecules, or fragments thereof, are in contact with their van der Waals surface. We sample the density of states of molecular clusters in the space spanned by the coarse-grained degrees of freedom. From here, we calculate the sum over states and free energy of the cluster at a temperature of interest by histogram reweighting. The method allows to calculate the energy and entropy contributions to the cluster free energy explicitly. For two components, we then obtain the excess free energy of mixing and the Flory–Huggins χ-parameter, and their energetic and entropic contributions. We present two applications of the method: a simple liquid mixture of hexane and nitrobenzene, and a series of polymer blends. In the case of hexane/nitrobenzene, we compare to alternative simulation methods; here we find that the energy of mixing alone is too high to explain the critical point. By including the excess entropy of mixing, however, the predicted phase behavior is in reasonable agreement with experiment. The tendency of calculations based on average energy alone to overestimate the χ-parameter is also apparent in the polymer blend calculations. © *2008 American Institute of Physics*. [DOI: 10.1063/1.2943211]

I. INTRODUCTION

The advent of nanotechnology has induced an advance in simulation science and computational modeling, traditionally bound to the electronic, atomistic, or continuum scale. In particular, the need to adjust the computer model and simulation method to the phenomenological nanoscale has led to the development of coarse- and fine-graining strategies, enabling seamless *in silico* multiscale modeling and simulation workflows.[1,2]

A sketch of a multiscale modeling workflow starts with electronic structure calculations to study quantum phenomena and derive a classical force field. Molecular simulation methods use the force field to study molecular phenomena and to determine coarse-grained descriptors. These descriptors in turn feed into mesoscale simulation methods to study mesoscale phenomena and predict a morphology phase diagram. Finally, macroscopic methods, such as finite-element methods or computational fluid dynamics, can probe transport and mechanical properties of the composite morphology by solving continuum equations. Such workflows are extremely versatile and have been used in a wide variety of cases, such as conductivity of nanotube dispersions,[3] precipitation membranes,[4] protein fracture,[5] hair mechanics,[6] etc.

In multiscale workflows, mean-field models often play a crucial role of levering coarse-grained descriptors to mesoscale morphology. An archetypal mean-field model is the Flory–Huggins (FH) lattice model for binary mixtures, in which all interactions are reduced to a single adjustable parameter χ, specifying the relative affinity between two components.[7] More modern simulation approaches, such as self-consistent field theory (SCF)[8,9] and dynamic density-functional theory,[10] can be interpreted as generalizations of FH. Also dissipative particle dynamics (DPD), originally developed for simulating hydrodynamic flow,[11] can be cast into the FH structure,[12] where particles experience nearest-neighbor interactions, scaled by a single parameter χ.

Despite its obvious importance in modeling, the calculation of χ from first principles has remained a challenge. Part of the problem is the ambiguity in defining χ. Strictly speaking, mean-field theory defines χ as the parameter minimizing the variational free energy, but such minimization is generally not feasible. Instead, χ typically is left as a fitting parameter, used to make the model effective toward predicting certain (experimental) properties. Since χ measures the affinity between two components, it is naturally derived from properties of a system in which both components are present. In the phase-separated state, properties of the interface[13] (e.g., tension) or binodal concentrations[12] can be used to fit χ. In the mixed state, the enthalpy of mixing is a candidate to fit χ.[14] Alternatively, it is possible to consider the pure components only and describe the interaction using an empirical mixing rule (e.g., Berthelot).[15] Such mixing rules generally lack a physical basis, however, which makes the prediction of phase behavior of liquid mixtures cumbersome.[16]

The aim of this work is to investigate and assess ways to calculate χ-parameters from first principles. We take the point of view that in mean-field models χ is a measure for the relative stability of a coordination shell. We thus focus on ensembles of coordination shell realizations, or molecular clusters, and consider the energy and entropy associated with such ensembles. By treating different cluster compositions in succession, we are able to obtain χ-parameters with explicit

[a)]Electronic mail: reiniera@accelrys.com.

 © 2008 American Institute of Physics

Reuse of AIP Publishing content is subject to the terms: https://publishing.aip.org/authors/rights-and-permissions. Downloaded to IP: 129.27.168.41 On: Sun, 04 Sep 2016 07:33:05

account for excess entropy. We validate the method with application to a liquid mixture and various polymer blends. Calculation of other mesoscale parameters, such as bond and angle descriptors, and simulation of the resulting mesoscale model is beyond the scope of this work.

The paper is organized as follows. In Sec. II, and partly in the Appendix, we derive the free energy as a function of coarse-grained coordinates (Secs. II A and II B). We then formulate this free energy in terms of a cluster model, where each bead contributes a cluster free energy (Sec. II C). To determine the cluster free energies, we consider an ensemble of clusters, and calculate the energy and entropy (Sec. II D) using the ensembles density of states (DOS) (Sec. II E). After generalization to mixtures, we arrive at an expression for the χ-parameter, which includes energy as well as excess entropy terms (Sec. II F). In Sec. III we apply the method to a molecular mixture and to polymer blends, the results of which are discussed in Sec. IV. Comparison with molecular dynamics (MD) and quantitative structure-active relationship (QSAR) calculations is made in selected cases (Sec. II G). The paper is concluded in Sec. V.

II. THEORY

A. Coarse graining

There are essentially two ways to arrive at a mesoscale model, which may be termed "bottom up" and "top down." The top-down approach starts from a set of observables and asks for a fine-grained representation of these data. For example, one is given a diffraction pattern and asks to reconstruct three-dimensional configurations of particles reproducing this pattern.[17] Or one wishes to reverse engineer a system that yields a desired phase diagram or rheology. These problems are reminiscent of so-called inverse problems.[18]

Alternatively, one may already have a fine-grained model, but wishes to filter for data relevant to the properties of interest, known as bottom up, *ab initio*, or coarse graining. Of course, in practice, information may flow from either side. In the following, we will focus on coarse graining. Two stages can be identified in the process of coarse graining: first, the selection of the degrees of freedom in the fine-grained model relevant to the phenomena one wishes to study, followed by the construction of the mesoscale model in terms of the selected coordinates.

Let the system to be coarse grained be described by a potential v. Let us denote the selected N coordinates by R. The remaining n coordinates, rendered irrelevant, will be called "bath," denoted by r. With this division in place, it is straightforward to show (see Appendix) that the function $U(R)$ given by

$$e^{-\beta U} = \int d\tilde{r} e^{-\beta v} \tag{1}$$

provides the best description of the coarse-grained system in variational sense, where $\beta = 1/(k_B T)$ with k_B the Boltzmann constant and T the absolute temperature. We use a tilde to express an infinitesimal in reduced units, i.e., $d\tilde{r} = dr/\Lambda^n$, where Λ is the resolution of r-space.

By "best description," we mean that the difference between the free energy of the coarse-grained model (described by U) and that of the microscopic model (described by v) is minimal.[19] In fact, for U defined in Eq. (1), this difference is a constant, and consequently all properties that depend only on R in the microscopic system are recovered. Thus, Eq. (1) provides a way to adjust the sampling space to the properties of interest without losing predictability. The price paid is that U depends not only on R but, unlike v, also on the thermodynamic variables β, n, and V specifying the bath. The function U must thus be interpreted as a free energy and can formally be separated in an energy function $E(R)$ and an entropy function $-TS(R)$.

Once U is known for a reference state R_0, its value at state R can, at least in principle, be constructed using any of the free energy methods.[20] A direct method to evaluate $U(R)$ is to measure the distribution Ψ of R in the microscopic system,[21] from which $U(R) = U(R_0) - (1/\beta)\ln[\Psi(R)/\Psi(R_0)]$. Another approach to obtain $U(R)$ is by integration[22] of $\partial_R U$; as follows directly from Eq. (1) (see Appendix), $\partial_R U$ at R equals the average of the force $\partial_R v$ evaluated at R. In other words, U is the potential of the mean force $\langle \partial_R v \rangle_R$, such that $U(R) = U(R_0) + \int_{R_0}^{R} dR' \langle \partial_R v \rangle_{R'}$.

It is clear, however, that as the dimensionality of R increases, the aforementioned free energy methods are not feasible if the coordinates R are strongly correlated. Moreover, the outcome of such an undertaking will generally be a complicated function of all R, and hence impractical for subsequent mesoscale simulations, for such simulations are only efficient with pairwise additive potentials.

For this reason, $U(R)$, or at least the part responsible for nonbonded interaction, is typically approximated by a pairwise summation of central potentials u_2,

$$U(R) \approx \sum_{i=1}^{N-1} \sum_{j=i+1}^{N} u_2(R_{ij}), \tag{2}$$

where the sum runs over all pairs of interaction sites (referred to as "beads"), and R_{ij} is the distance between beads i and j. Using the same argument underlying Eq. (1), one may ask for the mean-field solution of Eq. (2), by minimizing the variational free energy. Such "mean-field pair potentials," however, are generally hard to obtain.[19] Alternatively, "effective" pair potentials u_2 can be constructed by matching certain properties of the exact potential of mean force U. For example, by matching the force,[23] structure,[24] or heat of vaporization[25] of the microscopic system.

At this point, we should mention that although Eq. (1) provides the exact solution to recover the thermodynamics on the mesoscale, it is not sufficient to recover the exact dynamics of the mesoscale system.[22] The bath not only introduces entropy into the conservative part of the equation of motion but also acts as a thermostat to the remaining coordinates. That is, the coordinates R will also experience friction and fluctuating forces, to account for the absence of the bath coordinates. A method to calculate those contributions was introduced earlier.[22] In this study, however, we will focus on the thermodynamic part.

Reuse of AIP Publishing content is subject to the terms: https://publishing.aip.org/authors/rights-and-permissions. Downloaded to IP: 129.27.168.41 On: Sun, 04 Sep 2016 07:33:05

B. Mean-field models

The aim of the current work is not to construct an effective pair potential function u_2. Rather, we aim to link the potential of mean force U to the mean-field models underlying mesoscale simulations methods, such as DPD and SCF methods.[10,33] In the following, we shall use the DPD-formulation of Warren.[26]

We assume that U can be written as a sum over all beads,

$$U(R) \approx \sum_{i=1}^{N} a(z_i). \tag{3}$$

The free energy associated with a bead, a, is a function of its coordination, or "local density," $z(R)$. To first order in z, we have

$$a(z) = a^{(0)} + a^{(1)} z. \tag{4}$$

We can read this expansion in terms of a cluster model: Each bead interacts with a mean field of total energy $Na^{(0)}$, plus its coordination shell of z neighbors. When a bead enters the coordination shell of the bead, it adds an additional $a^{(1)}$ to the energy of this bead [in this convention, the total energy increases by $2a^{(1)}$]. The zeroth-order term $a^{(0)}$ sets the reference state and can be conveniently set to zero.

On a lattice, such as FH, z counts the number of neighboring sites that are occupied. In a continuum model, such as DPD, the coordination is defined using a weight function,

$$z_i(R) = \sum_{j=1|j\neq i}^{N} \omega(R_{ij}). \tag{5}$$

The weight function $\omega(s)$ decreases with s to zero, and sets the correlation length of the model. Several forms are in use depending on the mean-field model, such as Gaussians $\omega(s) \propto \exp(-\alpha s^2)$ (in certain density functional theories) and truncated harmonic functions $\omega(s|0<s<R) \propto (1-s/R)^2$ (in DPD).

In a mean-field model, the coordination z and the mean-field neighbor interaction $a^{(1)}$ are independent; an additional bead simply makes a mean-field contribution to the energy. In a many-body system, such as DPD, this is generally not the case. However, using truncated harmonic weight functions, Groot and Warren found[12] that the average coordination of a bead, $\langle z_i \rangle$, is to good approximation independent of the energy scaling $a^{(1)}$. Moreover, they found[12] that the coordination becomes constant as the density N/V increases. Thus the DPD-model is expected to behave like a mean-field model under those conditions. This is in contrast to hard-core (e.g., Lennard–Jones) weight functions, which typically introduce strong correlation, breaking down the mean-field approximation.

For completeness, we notice that the pair structure of the weight function, Eq. (5), guarantees a pairwise model of central forces: The force derived from Eq. (3), under the condition, Eqs. (4) and (5), is equal to the force derived from Eq. (2), when $u_2(s) = a^{(1)}\omega(s)$. This makes Warren's one-body formulation[26] equivalent to the original formulation of DPD in terms of pair forces.[11] The advantage of a one-body formulation lies in the potential of extending the expansion, Eq. (3), to terms of higher order in z. This provides a way to include many-body correlations and still use (now density-dependent) pairwise forces,[27] somewhat analogous to the use of a "glue potential" in electron structure calculations.[28]

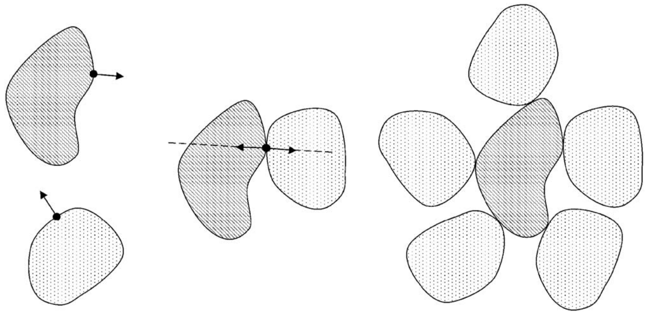

FIG. 1. Illustration of the cluster sampling algorithm in two dimensions; a reference molecule is coordinated with molecules by drawing a random binding site on the respective surfaces (left) and aligning the surface normals (middle). In three dimensions an additional rotation about the normal is carried out. Repeating the algorithm for multiple neighbors generates a molecular cluster configuration (right).

C. Cluster model

Our aim at this point is to provide an expression for the "cluster free energy" $a(z)$ in Eq. (4), where a cluster corresponds to a coordination shell of molecules (or fragments thereof). For this, we first need to define a cluster, analogous to Eq. (5). In line with the FH-model, we make the nearest-neighbor approximation and define the coordination z of a reference molecule as the number of molecules in direct contact. Two molecules are said to be in contact if their van der Waals surfaces "touch" (are tangent), without overlapping with any of the other molecules, as illustrated in Fig. 1.

A coordination pair can thus be constructed by drawing a point on each molecular surface, aligning the associated normals, and translating along the normal direction until the surfaces are tangent (plus an arbitrary rotation about the normal axis). An n-body cluster is constructed by adding a molecule to a $(n-1)$-body cluster, using the condition that if selected normals and rotation result in overlap with any of the $n-1$ molecules present, the configuration is discarded.

Clearly, a cluster can be realized in a variety of ways. The degrees of freedom (or bath coordinates) underlying this degeneracy are the binding sites on the molecular surface and the normal axis rotation. In addition, each molecule may possess multiple conformations, with associated surfaces. Since $\exp[-\beta a(z)]$ is the probability density for a cluster of z neighbors in the ensemble of realizations, we associate $a(z)$ with the free energy of such an ensemble of clusters. The calculation of $a(z)$ thus requires enumeration of a representative part of clusters, from which the partition function for this ensemble can be calculated.

To formalize the above definition of the cluster free energy a, let q_i be the conformation of molecule i, where $i=0$ labels the reference molecule and $i=1,\ldots,z$ the coordinating molecules. Let $S_i(q_i)$ be the surface area of molecule i in conformation q_i, and let $\mathbf{S}_i$ denote a normal on this surface. Since the contact point of molecule i follows from $\mathbf{S}_0$ and $\mathbf{S}_i$,

Reuse of AIP Publishing content is subject to the terms: https://publishing.aip.org/authors/rights-and-permissions. Downloaded to IP: 129.27.168.41 On: Sun, 04 Sep 2016 07:33:05

we write (half) the energy of contact molecule i as $v_i(\mathbf{S}_0, \mathbf{S}_i, \psi_i)$, where ψ_i is the rotation about the normal. The cluster energy then follows as $v = \Sigma_{i=1}^{z} v_i$.

In analogy with Eq. (1), the free energy associated with a cluster $a(z)$ can now be written down as a sum over states,

$$e^{-\beta a} = \int d\tilde{q}_0 \oint d\tilde{\mathbf{S}}_0 \prod_{i=1}^{z} \int d\tilde{q}_i \oint d\tilde{\mathbf{S}}_i \int d\tilde{\psi}_i e^{-\beta v} \Theta, \tag{6}$$

where Θ selects the allowed states, i.e., $\Theta(\mathbf{S}_0, \{\mathbf{S}_i, \psi_i\}) = 1$ for configurations without overlap, and 0 otherwise. As before, a tilde denotes the infinitesimals in reduced units, such that Eq. (6) equates two dimensionless quantities. The surface integrals run over each point on the respective surfaces with associated normal $\mathbf{S}$.

For spherical surfaces, the free energy for a cluster of two molecules, $a(z=1)$, according to Eq. (6), is simply the van der Waals energy at contact, v. In general, however, the molecular surface is a conjunction of spherical surfaces, complicating the evaluation of the surface integrals in Eq. (6). At the same time, the theory is not meant to model strongly aspherical molecules, since the orientational degrees of freedom are to be integrated out. This then motivates to replace the exact surface integral in Eq. (6) by an integration over a unit sphere around the center of mass of the molecule (surface area $S_i = 4\pi$). This has the advantage that the normals can be drawn independent of the conformation. The exact point of contact is then determined by translation about the axis joining the centers of mass. Consequently, the distribution of binding sites over the surface will be nonuniform, but the bias on the resulting interaction parameters should be small for typical subjects to coarse graining.

A bead may also correspond to part of a molecule, for example, one or more repeat units in a polymer. This means that docking near the connection points is restricted. In the FH-model, this is resolved by reducing the lattice coordination z by 2. As a continuum generalization, we will use Θ to exclude configurations in which molecules are in contact via atoms near the connection point. This is accomplished by first saturating the fragments with small end groups, and subsequently excluding the atoms in those end groups from contact. To obtain the interaction with a repeat unit in a (infinitely long, linear) polymer, for example, exclusion groups are added on both side of the fragment. For the same repeat unit at the end, only one side would be restricted.

Finally, in some coarse-grained models, a bead corresponds to more than one molecule. This case can be incorporated by letting q_i represent the conformational degrees of freedom of such small cluster of molecules. Alternatively one may use a single molecule and apply a scaling relation in the number of molecules, provided such scaling relation can be found. This scenario, however, will not be part of this study.

D. Free energy components

It is clear from Eq. (6) that a is a free energy, as it incorporates all states of internal (bath) coordinates for which the coarse-grained model no longer accounts explicitly. The free energy contains the average energy of contact over all internal coordinates, as well as an entropy associated with the distribution of energy over all internal coordinates.

To make the separation of a into energy and entropy terms explicit, let us abbreviate all degrees of freedom, and associated normalization, by the dimensionless variable r, such that Eq. (6) reads

$$\begin{aligned} e^{-\beta a} &= \int dr e^{-\beta v} \Theta \\ &= \int d\epsilon e^{-\beta\epsilon} \left[\int dr \delta(\epsilon - v(r)) \Theta \right] \\ &\equiv \int d\epsilon e^{-\beta\epsilon} g \equiv Z. \end{aligned} \tag{7}$$

The second line separates out the DOS $g(\epsilon)$, by adding an integration over the accessible energy space. The DOS simply measures the number of allowed configurations corresponding to a particular energy value ϵ. The DOS is normalized such that $\int d\epsilon g$ is the fraction of phase space corresponding to allowed states. The partition function Z is seen to approach this value as temperature increases ($\beta \to 0$).

Let $P(\epsilon;\beta)$ be the probability density for a cluster to have an energy ϵ at temperature β,

$$P = \frac{e^{-\beta\epsilon} g}{Z}, \tag{8}$$

which is normalized to unity at any β according to Eq. (7). It is now straightforward to write down the energy and entropy contributions to the free energy,

$$a = -\frac{1}{\beta} \ln Z = \int d\epsilon P \epsilon + \frac{1}{\beta} \int d\epsilon P \ln(P/g) \equiv e - Ts, \tag{9}$$

where $s = -k_B \int d\epsilon P \ln(P/g)$ defines the entropy associated with the bath space. We can distinguish two entropy contributions: a contribution related to Θ selecting the allowed states, and a temperature-dependent contribution related to the distribution of energy over the allowed states.

Further insight into Eq. (9) can be obtained by considering the special case of a Gaussian DOS with average μ and deviation σ, i.e., $g(\varepsilon) \propto \exp\left(-\frac{1}{2}(\varepsilon - \mu)^2/\sigma^2\right)$. From Eq. (9), it then follows that $e = \mu - \beta\sigma^2$ and $Ts = -\frac{1}{2}\beta\sigma^2$ (hence $a = \mu - \frac{1}{2}\beta\sigma^2$). The "degeneracy" of the states σ is thus directly proportional to the entropy. For a nondegenerated ground state $\sigma \to 0$ and $g(\varepsilon) \to \delta(\varepsilon - \mu)$, the entropy is seen to vanish, along with the heat capacity $C_V = \partial_T e = k_B(\beta\sigma)^2$, as expected from thermodynamics.

E. Cluster sampling method

To sample the DOS $g(\epsilon)$ in Eq. (7), a large number of random states, or cluster configurations, r are generated. The clusters are built by packing molecules onto a base molecule, as described in Sec. II C and Fig. 1. To each state the contact condition is applied; if the condition is satisfied, the cluster binding energy ϵ is evaluated, defined as the energy of interaction of the reference molecule with all of its coordinating neighbors (excluding the interaction energy between the

Reuse of AIP Publishing content is subject to the terms: https://publishing.aip.org/authors/rights-and-permissions. Downloaded to IP: 129.27.168.41 On: Sun, 04 Sep 2016 07:33:05

neighbors). The ensemble distribution of this binding energy is the DOS, normalized by the fraction of allowed configurations.

The energy distribution at a particular temperature, $P(\epsilon,\beta)$, is calculated from the DOS by reweighting, using Eq. (8). From the energy distribution P follows the average energy e using Eq. (9), and the sum over states Z as well as the free energy a using Eq. (7). The entropy then follows by subtraction, $s=(e-a)/T$.

The cluster sampling can be carried out at constant coordination number z or constant "chemical potential" μ. Strictly speaking the latter requires the introduction of a Gibbs free energy through reweighting the sum over states of all constant z ensembles with a Boltzmann factor $\exp(-\beta\mu z)$, where μ is the chemical potential of the bath surrounding the base molecule. A more pragmatic way of fixing the number of candidate coordinating molecules is chosen here. For each candidate molecule, an attempt is made to coordinate the reference molecule, under the contact condition, until the reservoir of candidates is exhausted. In practice this also leads to a distribution in the coordination number.

In terms of sampling methods, the above method of calculating the DOS g is a "simple" sampling of phase space.[20] Temperature does not play a role in the sampling, and is just an *a posteriori* rescaling. Another approach would be "importance" (e.g., Monte Carlo) sampling of P at some reference temperature, and reweighting to get g. Since the phase space is relatively small, we prefer direct sampling of g, which avoids having to choose a reference temperature. This does mean that the bin width of the DOS histogram puts numerical limits to the temperature at which the distribution can reliably be reweighted. Assuming that k_BT should be at least larger than the bin width of the DOS, we estimate for this study $T \gtrsim 10$ K.

F. Mixtures

The cluster model is readily generalized to more than one component, which provides a way to predict the free energy of mixing. This energy can be written in terms of a single parameter, χ, which then forms the bridge between the cluster calculations and mesoscale models.

For C components, a coordination shell is specified by C coordination numbers $(z_1,\ldots,z_C)$, as well as the type of the coordinated particle. The potential of mean force Eq. (3) generalizes as

$$U = \sum_{c=1}^{C} \sum_{i\in c} a_c(z_{1,i},\ldots,z_{C,i}), \tag{10}$$

where a_c is the free energy of a particle of component c. To first order in the arguments

$$a_c(z_1,\ldots,z_C) = a_c^{(0)} + \sum_{d=1}^{C} a_{cd}^{(1)} z_d. \tag{11}$$

Disregarding the reference terms $a_c^{(0)}$, we can thus write the total energy in terms of the interaction energy $U_{cd} \equiv \sum_{i\in c} a_{cd}^{(1)} z_{d,i}$,

$$\begin{aligned} U &= \sum_{c=1}^{C}\sum_{d=1}^{C} U_{cd} \\ &= \sum_{c=1}^{C-1}\sum_{d=c+1}^{C}\left[U_{cd}+U_{dc}-\frac{x_d}{x_c}U_{cc}-\frac{x_c}{x_d}U_{dd}\right]+\sum_{c=1}^{C}\frac{U_{cc}}{x_c}, \end{aligned} \tag{12}$$

where x_c is the fraction of molecules of component c and $\lim_{x_c\to 0} U_{cc}/x_c = 0$ is understood. The second line separates out a symmetric mixing term and a pure component term. To reduce notation, it is convenient to express Eq. (12) in units of k_BT and per particle. Introducing $u=\beta U/N$ and $u_{cd} = \beta U_{cd}/N$, we thus have

$$u = \sum_{c=1}^{C-1}\sum_{d=c+1}^{C}\left[u_{cd}+u_{dc}-\frac{x_d}{x_c}u_{cc}-\frac{x_c}{x_d}u_{dd}\right]+\sum_{c=1}^{C}\frac{u_{cc}}{x_c}. \tag{13}$$

If the components are ideally mixed, the coordination shell will reflect the composition in the mixture, that is, on average $a_{cd}^{(1)} z_{d,i} \approx x_d a_{cd}$, where a_{cd} the free energy associated with a particle of component c, fully coordinated by particles of type d. In this approximation, we thus have $u_{cd} \approx x_c x_d \beta a_{cd}$ and substitution in Eq. (12) yields

$$u = \sum_{c=1}^{C-1}\sum_{d=c+1}^{C} x_c x_d \chi_{cd} + \sum_{c=1}^{C} x_c u_c^{\circ}, \tag{14}$$

which introduces the (symmetric) interaction matrix with components

$$\chi_{cd} = \beta(a_{cd}+a_{dc}-a_{cc}-a_{dd}). \tag{15}$$

In Eq. (14), we have replaced βa_{cc} in the last term by u_c°, the energy per particle in the pure component c, in units of k_BT. A circle is used to denote the pure components.

Each term in Eq. (15) can be evaluated using the cluster method, Eq. (6); for example, a_{cd} is the free energy of a cluster of a molecule of component c coordinated by molecules of component d. Using the separation, Eq. (9), of a_{cd} in energy and entropy terms, the χ-parameter can be separated accordingly, into an energy term,

$$\chi_e \equiv \beta(e_{cd}+e_{dc}-e_{cc}-e_{dd}) \tag{16}$$

and an entropy term,

$$\chi_s \equiv \frac{1}{k_B}(s_{cd}+s_{dc}-s_{cc}-s_{dd}), \tag{17}$$

such that $\chi=\chi_e-\chi_s$. The sign of χ_e is determined by the relative stability of the mixture compared to the pure state. The sign of χ_s is determined by the relative disorder in the mixture compared to the pure state. Mixing two dislike species, we thus expect both χ_e and χ_s to be positive. All χ-parameters are dependent of the temperature. With the cluster sampling method described below, this relation can be evaluated explicitly. The knowledge of $\chi(T)$ provides a way to construct a phase diagram.

We note that Eq. (14) contains only the excess free energy. The total free energy is obtained by adding an ideal contribution due to the fact that particles of different components are distinguishable. This combinatorial term is calcu-

Reuse of AIP Publishing content is subject to the terms: https://publishing.aip.org/authors/rights-and-permissions. Downloaded to IP: 129.27.168.41 On: Sun, 04 Sep 2016 07:33:05

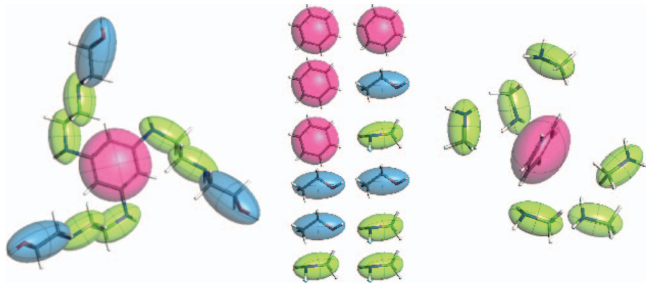

FIG. 2. (Color) Illustration of the parametrization method applied to a dendrimer: A dendrimer molecule is divided in fragments at the resolution required (left); Each pair combination of fragments is considered in turn (middle); For each pair, clusters are build for all four permutations; the free energy of binding is calculated in each cluster (right). Combining the cluster free energies leads to the χ-parameter associated with this pair of fragments.

lated from the number of permutations of components over the number of particles, discounting indistinguishable ones, i.e., $\Omega=N!/N_1!/\cdot\cdot/N_C!$. With $S_{\text{id}}=k_B \ln \Omega$ and Stirling's approximation,

$$S_{\text{id}}=-k_B N \sum_{c=1}^{C} x_c \ln x_c.$$

The total free energy then follows as $F=U-TS_{id}$.

Finally, the free energy of mixing is obtained by subtracting the contribution of the pure components. For two components A and B, the free energy of mixing (per particle in units of k_BT) is

$$\Delta f = x_A \ln x_A + x_B \ln x_B + \chi_e x_A x_B + \chi_s x_A x_B \tag{18}$$

composed of two ideal terms, the energy of mixing and the excess entropy of mixing. This is equivalent to the FH model with an explicit excess entropy term that scales in the same way as the energy for a regular mixture.

A summary of the coarse-graining workflow is sketched in Fig. 2. A molecular system is divided into fragments which are classified into various types according to their chemical identity. The fragments correspond to beads in a coarse-grained description. The mean-field model of the bead system requires a χ-parameter for each pair of bead types. This parameter is obtained by generating a large number of fragment clusters and calculating the energy of interaction of each cluster as detailed above. The distribution of this energy leads to the associated free energy. After collecting the free energies for all combinations of components, the matrix of χ-parameters is calculated from Eq. (15).

At this point, we should mention a related method reported in 1992 by Fan *et al.*[29] which applies their "Molecular Silverware" algorithm to molecular pairs with the aim of predicting phase diagrams and other thermodynamic properties of polymer blends and solutions directly from mean-field theory. Whereas our aim is to estimate mesoscale model parameters, rather than directly invoking thermodynamic theory, the approach taken has similarities and a discussion of the differences is in place.

The current method differs from theirs in two important ways: Fan *et al.* sampled the interaction energy of isolated molecular pairs, whereas the current method samples the interaction within the cluster environment. The current method can thus account for potential correlation between the cluster size and the interaction energy. For example, it is likely that the binding energy decreases with cluster size, since at finite temperature the most favorable binding sites will be saturated first. Simply multiplying this favorable pair-interaction energy with the cluster size would then overestimate the actual interaction energy in the cluster. If this correlation is not symmetric in the permutation of components, this will affect the value of χ.

The second difference pertains to the calculation of the χ-parameter. Whereas Fan *et al.* calculated the χ-parameter using the average interaction energy, the current method uses the integrated energy distribution to calculate the free energy, a. The difference is the entropic contribution to the free energy, s. Upon mixing, this leads to the entropic interaction parameter, χ_s. One of the aims of this work is to quantify the entropic contribution and see if this can be safely neglected or not.

G. Alternative methods to determine χ

In this section, we briefly mention some related methods that are used to estimate the mean-field interaction parameter χ. We will apply these methods to test and validate the proposed cluster approach detailed above. We will only consider energy-based methods and disregard theories relating χ to, for example, the interfacial tension.[30]

The first method follows from inverting Eq. (14), expressing χ_{cd} in terms of the energy of mixing. For two components, A and B, mixed in ratio $x_A:x_B$ and neglecting all excess entropy effects,

$$\chi = \frac{u - x_A u_A^{\circ} - x_B u_B^{\circ}}{x_A x_B} = \frac{\Delta u_{\text{mix}}}{x_A x_B}, \tag{19}$$

where Δu_{mix} is the energy of mixing expressed in units of k_BT and per particle.

So far the theory has not explicitly considered the volume a molecule occupies. For components that differ in size, a better approximation to $a_{cd}^{(1)} z_{d,i}$ in Eq. (11) would be $y_d a_{cd}$, where y is the volume fraction, rather than the mole fraction x. Let v be the volume per particle and v_c the volume associated with component c, per particle of component c, then $y_c=x_c v_c/v$. Introducing the energy densities $\hat{u}=u/v$, and $\hat{u}_c^{\circ}=u_c^{\circ}/v_c$, Eq. (13) gives

$$\hat{u} = \frac{1}{v} \sum_{c=1}^{C-1} \sum_{d=c+1}^{C} x_c x_d \chi'_{cd} + \sum_{c=1}^{C} x_c \hat{u}_c^{\circ}, \tag{20}$$

which introduces the interaction matrix as

$$\chi'_{cd} = \beta\left(\frac{v_d}{v} a_{cd} + \frac{v_c}{v} a_{dc} - a_{cc} - a_{dd}\right), \tag{21}$$

which differs from Eq. (15) by a volume normalization of the cross terms. Expressing χ' in terms of the (cohesive) energy densities for two components then gives

Reuse of AIP Publishing content is subject to the terms: https://publishing.aip.org/authors/rights-and-permissions. Downloaded to IP: 129.27.168.41 On: Sun, 04 Sep 2016 07:33:05

$$\chi' = v\frac{\hat{u} - x_A\hat{u}_A^\circ - x_B\hat{u}_B^\circ}{x_Ax_B}. \quad (22)$$

The volume v in Eq. (22) is the volume per particle, which may depend on the composition. The parameter χ' applies to a model in which the particle has a volume v.

A common approximation is to replace the mixture by an empirical mixing rule. In this case, only the pure components have to be simulated. A well-known mixing rule is the Berthelot rule, which approximates the interaction between two components as $a_{cd}^2 = a_{cc}a_{dd}$. Introducing the solubility parameter $\delta_c^\circ = \sqrt{-\hat{u}_c^\circ/\beta} = \sqrt{-a_{cc}/v}$, it follows from Eqs. (15) and (14)

$$\chi = \beta v(\delta_A^\circ - \delta_B^\circ)^2. \quad (23)$$

It is clear that χ in Eq. (23) is always positive; consequently, the energy of mixing, $\chi x_A x_B$, is positive, such that the Berthelot rule, for example, cannot be used to model endothermic mixing. Other empirical rules can be invented to workaround this limitation, for example, to assume $a_{cd}^2 = a_{cc}a_{dd}(1-\kappa_{cd})^2$; the physical meaning, e.g., temperature dependency, of such "similarity parameter" κ, however, remains cumbersome.

Despite the fact that all mixing behavior is captured by a simple rule, Eq. (23) often yields realistic phase behavior. Moreover, only pure component properties δ_c° have to be considered. Motivated by this success, much effort has been spent in the past to correlate the solubility parameters with functional groups within a molecule.[31] In such group-based methods, the molecule is first separated into groups for which correlations are available, and the solubility parameter for the molecule is then obtained by summing the contribution associated with each group. The interaction parameter χ is subsequently obtained from the solubilities using Eq. (23). This is the essence of QSAR-methods like those developed by van Krevelen and Fedors.[31]

To summarize this section, we have derived various expressions for the interaction parameter χ. Equation (15) estimates χ from the cluster free energies a_{ij} calculated from the DOS, Eq. (6). Equations (19) and (22) estimate χ by calculating the energy of mixing. Finally, Eq. (23) estimates χ from the solubility parameters under the Berthelot mixing rule.

III. RESULTS

In this section, we present the results of the calculation of χ for two cases. We first consider a "regular" mixture of liquids. The pure component contributions in Eq. (22) and the solubilities in Eq. (23) are calculated from MD-simulation of the pure components. The energy terms in Eqs. (19) and (22) were calculated from MD-simulations of a mixture over a range of compositions. Finally, the DOS, Eq. (6), was calculated from the cluster sampling method, from which the χ-parameters were abstracted using Eq. (15).

The second case study consist of polymer blends, the traditional subject of the mean-field theory of Flory and Huggins. We calculate the DOS using cluster sampling. We compare results with experimental data, as well as χ-parameters calculated from QSAR, using Eq. (23).

TABLE I. Molecular volume, energy, and energy density of hexane/nitrobenzene mixtures from simulations at atmospheric pressure and room temperature (x_h is the mole fraction hexane, v the volume per molecule, u the intermolecular energy per molecule, and $\hat{u}$ the energy density).

x_h	$\langle v \rangle$ (nm^3)	$\langle u \rangle$ (kJ/mol)	$\langle \hat{u} \rangle$ (MPa)
0	0.177	−46.4	−437
0.1	0.180	−44.2	−407
0.2	0.185	−41.2	−382
0.3	0.190	−38.8	−340
0.4	0.195	−36.8	−315
0.5	0.199	−34.6	−290
0.6	0.204	−32.5	−273
0.7	0.211	−30.4	−250
0.8	0.215	−28.9	−230
0.9	0.217	−28.2	−214
1	0.221	−27.4	−205

Throughout this study we use the COMPASS force field in atomistic calculations.[32,33] COMPASS is a force field based on *ab initio* calculations and optimized with empirical data of the condensed state. Electrostatics is incorporated through partial charges on the nuclei, calculated from bond increments. In the QSAR-calculations, we use both the methods of Fedors and van Krevelen.[31]

A. Liquid mixture

The mixture of nitrobenzene and hexane provides a convenient starting point to study liquid-liquid phase behavior, since the experimental phase diagram is reasonably symmetric in the composition with a single critical point close to room temperature[34] (T_c=292.27 K at atmospheric pressure at hexane fraction $x_{h,c}$=0.416).

The phase diagram according the FH-model can be obtained from Eq. (18); the binodal is symmetric in the composition, and reaches a critical point at $x_{h,c}$=0.5, at which χ=2. Hence, assuming the FH-model is a good model for the mixture of nitrobenzene and hexane, at room temperature we thus expect the calculation to result in $\chi_c \equiv \chi(T_c) \sim 2$.

We first present the result of the MD-simulations and QSAR-calculations used to calculate the input to Eqs. (19), (22), and (23). We then present the results of the cluster sampling method. Results are discussed in Sec. IV A.

1. Molecular dynamics

We simulated condensed states of nitrobenzene and hexane at various concentrations at a temperature of 298 K and a pressure of 1 atm. All configurations contain 100 molecules of which a fraction x is hexane, where x varies from 0 to 1 in ten steps.

Systems were set up using the AMORPHOUS CELL construction module,[33] by packing the required number of molecules in a cubic box at a density equal to the weighted average of the pure component densities. Energy minimization and brief equilibration at constant volume and tempera-

Reuse of AIP Publishing content is subject to the terms: https://publishing.aip.org/authors/rights-and-permissions. Downloaded to IP: 129.27.168.41 On: Sun, 04 Sep 2016 07:33:05

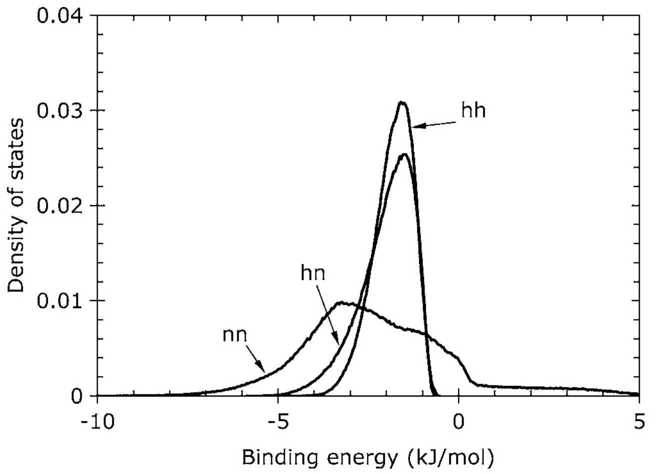

FIG. 3. DOS as a function of the binding energy, for molecular pairs nn_1, nh_1, and hh_1, where h=hexane and n=nitrobenzene. The curve for hn_1 is identical to nh_1. The abscissa shows the binding energy per molecule.

ture were followed by 150 ps dynamics run with an Andersen barostat and Nosé thermostat keeping pressure and temperature at 1 atm and 298 K, respectively. All simulations were carried out using the simulation module FORCITE in MATERIALS STUDIO.[33] Results were collected over the last 100 ps.

In the ensembles thus obtained, we measure the average volume per molecule $\langle v \rangle$, the average total intermolecular energy per particle $\langle u \rangle$, and the average energy density $\langle \hat{u} \rangle$. All results are reported in Table I and discussed in Sec. IV A.

2. Cluster sampling method

We sampled the DOS of molecular clusters following the procedure outlined in Sec. II E using Eqs. (7) and (6). We sample clusters under constant coordination number and constant number of attempts per cluster.

We first consider the DOS of clusters, where the coordination number was fixed at $z=1$, i.e., molecular pairs. A total of 10^6 pair structures were sampled. Results are shown in Fig. 3 for all combinations.

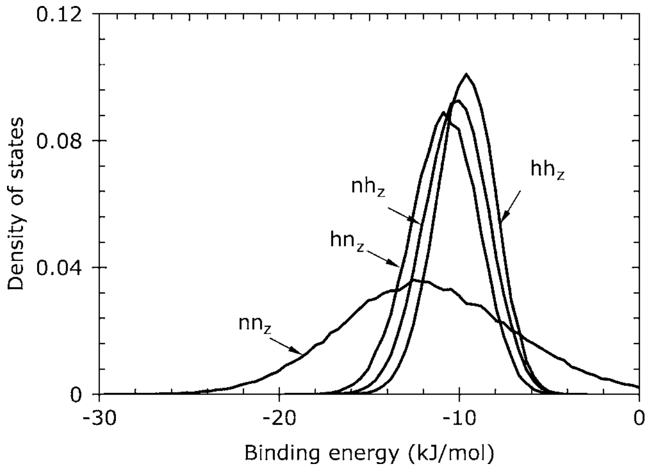

FIG. 4. DOS as a function of the binding energy, for molecular clusters nn_z, nh_z, hn_z, and hh_z, where h=hexane and n=nitrobenzene.

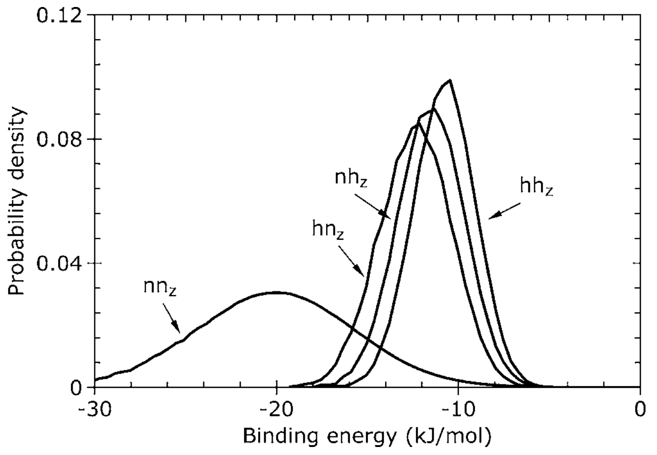

FIG. 5. Probability density at temperature 298 K as a function of the binding energy, for molecular clusters nn_z, nh_z, hn_z, and hh_z, where h=hexane and n=nitrobenzene.

The DOS was subsequently sampled "grand canonically" in which the coordination z was variable, and the number of candidate molecules for coordination was fixed at 20. A total of 10^6 clusters were sampled. The DOS are shown in Fig. 4. Figure 5 shows the probability density of the binding energy at room temperature, calculated by reweighting to room temperature according to Eq. (8). The energy parameters calculated from Fig. 5 via Eq. (6) are reported in Table III. Results are discussed in Sec. IV A.

B. Polymer blends

Six binary polymer blends where selected on the basis of industrial relevance and available experimental data, as listed in Table II. The blends together require seven different polymers components: polystyrene (PS), tetramethyl-bisphenol-A-polycarbonate (TMPC), poly(vinyl methylether) (PVME), polyisoprene (PI), poly(2,6-dimethyl-1,4-phenylene oxide) (PXE), poly(ethyleneoxide) (PEO), and poly(methylmethacrylate) (PMMA).

Small oligomers of three repeat units each were constructed for all components. The COMPASS energy of the structures was minimized, followed by a 1 ns *NVT*-simulation in vacuum, using a Berendsen thermostat at room temperature. Conformation were stored every 10 ps in a trajectory. The resulting trajectories serve as the structure input to the cluster model.

Next the DOS was sampled, by packing randomly selected conformations from the trajectories. The atoms in the terminal repeat units were marked as noncontact, indicating

TABLE II. Cluster free energy a, binding energy e, and excess entropy s at 298 K and coordination numbers z for a mixture of hexane (h) and nitrobenzene (n).

Cluster	$\langle a \rangle$ (kJ/mol)	$\langle e \rangle$ (kJ/mol)	$\langle s \rangle T$ (kJ/mol)	$\langle z \rangle$
hh_z	−11.0	−12.0	−1.0	5.5
nn_z	−19.7	−25.4	−5.7	5.6
hn_z	−12.6	−14.0	−1.3	5.8
nh_z	−11.6	−12.9	−1.3	5.3

Reuse of AIP Publishing content is subject to the terms: https://publishing.aip.org/authors/rights-and-permissions. Downloaded to IP: 129.27.168.41 On: Sun, 04 Sep 2016 07:33:05

TABLE III. Calculated interaction parameters for different polymer blends. χ is the computed value at 298 K with χ_e and χ_s the energetic and entropic contributions.

c	d	χ	χ_e	χ_s
PS	PXE	−0.03	0.04	0.07
PS	PMMA	0.23	0.45	0.22
PS	TMPC	0.83	2.06	1.23
PS	PVME	0.04	0.21	0.17
PS	PI	0.05	0.14	0.09
PEO	PMMA	0.006	−0.06	−0.066

that clusters in which the point of contact involves such atoms are rejected from the ensemble. Hence an allowed cluster does only have van der Waals contact between atoms in the central repeat unit. As before, the number of candidate coordinating molecules was set to 20. This way we generated an ensemble of 100 000 clusters.

In the ensemble of clusters, we measure the average coordination number and the free energy as well as the energy and entropy of binding (not shown). Combining the results using Eq. (15) leads to the χ-parameter, with Eqs. (16) and (17) providing a breakdown into energetic and entropic contributions, as tabulated in Table III. Results are discussed in Sec. IV B.

Single repeat units of all seven polymers were analyzed using the QSAR-module SYNTHIA.[33] The temperature was set to 298 K. The calculated molar volume and the solubility parameters using the methods of Fedors and van Krevelen are reported in Table IV. The results are discussed in terms of the χ parameter in Sec. IV B.

IV. DISCUSSION

In this section, we discuss the results of the Sec. III. Based on those results, we calculate the χ-parameters according to the various theories detailed in Sec. II. In selected cases, we also calculate the temperature dependence of the χ-parameters. Where available we compare to experimental data.

A. Liquid mixtures

We first compare the results of the MD-simulations of the pure components (first and last row of Table I) to the experimental data of hexane and nitrobenzene tabulated in Table V. Calculating the molecular volume as $v_{\mathrm{exp}}=M_i^\circ/\rho_i^\circ/N_a$ (N_a is Avogadro's number), we find 0.217 nm^3 (hexane) and 0.170 nm^3 (nitrobenzene). This is slightly less than, but within ~3% of, the volumes obtained from MD-simulation in Table I. Consequently, the mass densities in the simulation are slightly underestimated.

TABLE IV. Pure component properties of polymers at 298 K calculated using QSAR.

c	v (cm^3/mol)	δ, Fedors ($\sqrt{\mathrm{MPa}}$)	δ, Van Krevelen ($\sqrt{\mathrm{MPa}}$)
PEO	39.1	18.3	19.1
PI	76.6	16.9	17.2
PMMA	86.4	20.2	17.7
PS	97.0	20.1	19.5
PVME	55.4	17.7	18.3
PXE	110.8	19.5	20.5
TMPC	287.5	19.5	19.5

TABLE V. Experimental data (in SI units) at 298 K and 1 atm for pure components hexane (h) and nitrobenzene (n): Molar mass M°, mass density ρ°, enthalpy of evaporation (Ref. 35), ΔH° and solubility parameter (Ref. 36) δ°.

c	M° (kg/mol)	ρ° (kg/m^3)	ΔH° (kg/mol)	δ° ($\sqrt{\mathrm{MPa}}$)
h	0.086	659	366 ± 1	14.9
n	0.123	1199	456 ± 14	22.1

The average intermolecular energy per molecule $\langle u\rangle$ can be compared to the heat of vaporization, if we neglect the intermolecular energy of the vapor as well as neglect the difference in intramolecular energy between the liquid and the vapor. Using $u_{\mathrm{exp}}\approx -\Delta H_i^\circ M_i^\circ + RT$ gives −29.0 ± 0.1 kJ/mol (hexane), respectively, −53.6 ± 1.7 kJ/mol (nitrobenzene), compared to −27.4 ± 0.5 and −46.4 ± 0.9 kJ/mol in the simulations. The simulation underestimates the cohesive energy, in particular, for nitrobenzene. We speculate that the latter is mainly due to the charge distribution. It should be noted, however, that there is a substantial spread in the reported latent heat at room temperature for nitrobenzene,[35] presumably due to inaccurate heat capacities used in integration from boiling to room temperature.

Finally, the solubility parameters follow from the simulation data in Table I as $\delta=\sqrt{-\hat{u}}$. This results in 14.7 $\sqrt{\mathrm{MPa}}$ (hexane), respectively, and 20.9 $\sqrt{\mathrm{MPa}}$ (nitrobenzene), which is within 5% from experiment. Again the largest deviation from experiment is found for nitrobenzene. We notice that the correlation between energy and volume is negligible, and explicit averaging the energy density is not needed in this case.

We thus conclude that COMPASS predicts the pure component properties reasonably well. This is not so surprising given the fact that density and heat of vaporization are the typical quantities used in parametrizing the COMPASS force field. We will assume that COMPASS is also a good description of the mixtures under the same conditions. This assumption is reasonable since charge transfer and polarization are believed not to be dominant in the current systems.

$\chi x_h x_n$ is plotted in Fig. 6 according to Eqs (19) and (22), using the data of Table I. The reference volume v_{ref} in Eq. (22) is set to $\frac{1}{2}(v_h^\circ+v_n^\circ)$, as obtained from MD. To obtain the χ-parameter, we perform a least-squares fit of the data in Fig. 6 to the function $\chi x(1-x)$. The fit results in $\chi=4.0$ based on Eq. (19) and $\chi=5.5$ based on Eq. (22). The substantial scatter in the data reflects that the energy of mixing is a small number, compared to the energy of the pure components. For example, the maximum energy difference obtained at $x=\frac{1}{2}$ equates to a few kJ/mol, which is only a small fraction of the pure component energy. Hence the individual energies have to be calculated more accurate than that.

Reuse of AIP Publishing content is subject to the terms: https://publishing.aip.org/authors/rights-and-permissions. Downloaded to IP: 129.27.168.41 On: Sun, 04 Sep 2016 07:33:05

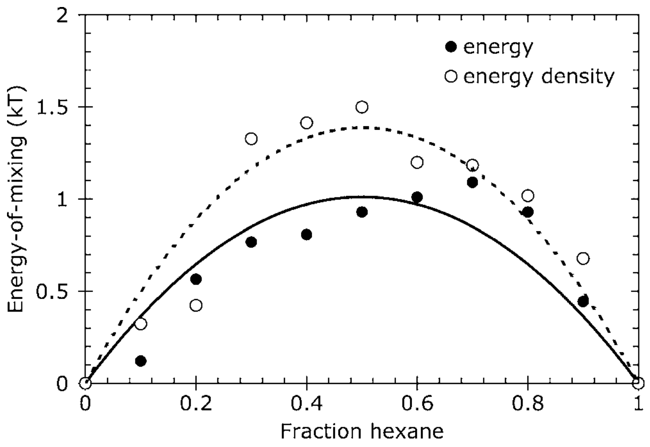

FIG. 6. Mixing energies for hexane-nitrobenzene mixtures calculated from Eqs. (19) and (22). Lines correspond to fits to the form $\chi x(1-x)$.

The explicit mixture routes are thus seen to overestimate the expected mean-field value ($\chi=2$) by at least a factor 2. Assuming COMPASS provides as good a model for the mixture as for the pure components, this suggests that the energy of mixing alone is not sufficient to describe the mixing process. In deriving Eqs. (19) and (22), the excess entropy of mixing was neglected. If we assume that this entropy term is positive, and scales in the same regular way ($\propto x_A x_B$), a smaller χ is to be expected. A positive entropy of mixing would indicate that the system looses (e.g., orientational) order on mixing. This is not unreasonable if the species are dissimilar. We will address this ansatz more quantitatively below.

Calculating χ from the simulated solubility parameters and the mixing rule Eq. (23), again using above reference volume, yields $\chi=1.8$. The implicit mixture route is thus seen to yield a much smaller value, closer to the mean-field result. This tentatively suggests that the mixing rule might be compensating for the neglect of excess entropy of mixing in the explicit mixture route.

We now turn to the molecular cluster calculations of hexane and nitrobenzene. We first consider the special case in which the coordination z is fixed to 1. As shown in Fig. 3, the DOS for hh and hn pairs is approximately Gaussian with a vanishing probability of finding configurations with positive binding energy. This is expected as charge polarization in hexane is very small (hydrogen atoms carry a charge of 0.053), and van der Waals interaction is dominant, which is attractive by construction. The DOS for nn pairs, on the other hand, is much more asymmetric, with a notable long positive tail. This tail represents the unfavorable electrostatic interaction in configurations in which the oxygen atoms (charge -0.428) are in close proximity.

From the DOS, we calculate for nitrobenzene an average energy of -2.2 kJ/mol if coordinated by another nitrobenzene, and -2.0 kJ/mol if coordinated by hexane. For hexane, the self-interaction adds up to -1.8 kJ/mol. Whereas these energies have no particular importance (they would correspond to the binding energy at infinite temperature), it already indicates that separating two n-h pairs will not involve a severe energy penalty.

Compared to the DOS for molecular pairs, the DOS for the clusters (Fig. 4) is more Gaussian, even for nitrobenzene, which in part can be explained by the central limit theorem: The unfavorable configurations that gave rise to a tail in Fig. 3 are partly compensated by more favorable binding elsewhere in the cluster. The cluster treatment also introduces a small asymmetry in the cross terms; the distribution for hexane coordinated by nitrobenzene (hn_z) has more nitrobenzene character, whereas the other combination (nh_z) has more hexane character.

The distribution of the cluster binding energies at room temperature is shown in Fig. 5 for the four permutations of components. The shape is similar to the DOS, but the scale of the x-axis is shifted to the lower energies. This is particularly noticeable for the nitrobenzene clusters, where the majority of the samples are seen to not contribute much to the ensemble at room temperature. This is a consequence of the simple sampling method as opposed to importance sampling. However, the number of samples is sufficiently large to obtain converged distributions, even in the case of nitrobenzene.

The average binding energy in the cluster environment calculated from the distributions in Fig. 5 are tabulated as $\langle e \rangle$ in Table II. This is (half) the average work required to remove the central molecule from the cluster. Substituting in Eq. (16) results in $\chi_e=4.2$. The energy-based parameter in this case is thus too high. The overestimation of energy-based parameters was also seen in the MD-simulations.

The coordination numbers $\langle z \rangle$ in Table II refer to average number of neighboring molecules in the cluster ensemble, by fixing the number of candidate molecules. Combined with the results for molecular pairs (Fig. 3), this provides a way to investigate correlation between the coordination number and the binding energy. For example, rescaling the DOS for pairs at 298 K gives a binding energy of -3.7 kJ/mol for nitrobenzene with nitrobenzene. As it is on average coordinated by 5.5 molecules of this type, the binding energy in a cluster of independently coordinating molecules would be -20.7 kJ/mol, compared to -20.1 kJ/mol in the cluster ensemble. In general, neglecting correlation overestimates the binding in the cluster environment. As correlation applies to all permutations in roughly the same amount, however, the net effect on the χ-parameter is small: $\chi_e=4.0$.

Finally, calculating the χ-parameter from the free energy of binding, tabulated as $\langle a \rangle$ in Table II, gives $\chi=2.6$. The entropy contribution to χ is thus seen to be $\chi_s=1.6$. The positive sign of χ_s indicates an increase in entropy on mixing. In other words, the order associated with the bath degrees of freedom decreases on mixing. In the current case, the bath degrees of freedom consist of the binding site on the molecular surface and the rotation about this contact. The "corrected" value of 2.6 is in reasonable agreement with the expected value of 2, based on the experimental phase diagram and FH-theory.

Whereas the above results apply to room temperature, the DOS can be reweighted at any temperature, which provides a way to obtain the temperature dependency of the χ-parameter, as shown in Fig. 7. The energy contribution is seen to decrease to zero monotonously, approximately reciprocally with temperature. An inverse temperature scaling is

Reuse of AIP Publishing content is subject to the terms: https://publishing.aip.org/authors/rights-and-permissions. Downloaded to IP: 129.27.168.41 On: Sun, 04 Sep 2016 07:33:05

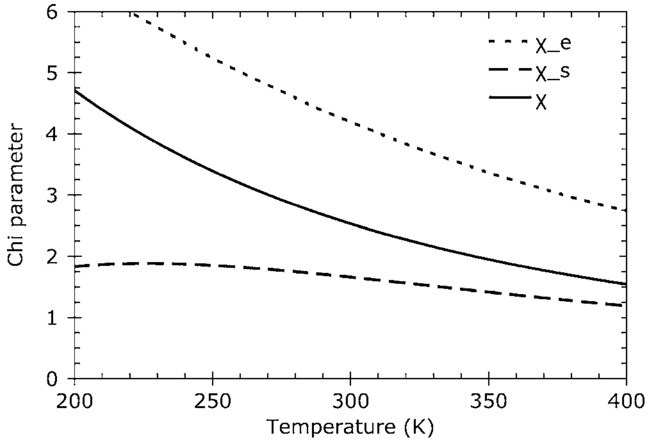

FIG. 7. Temperature dependency of the χ-parameter and its energy and entropy components for hexane and nitrobenzene.

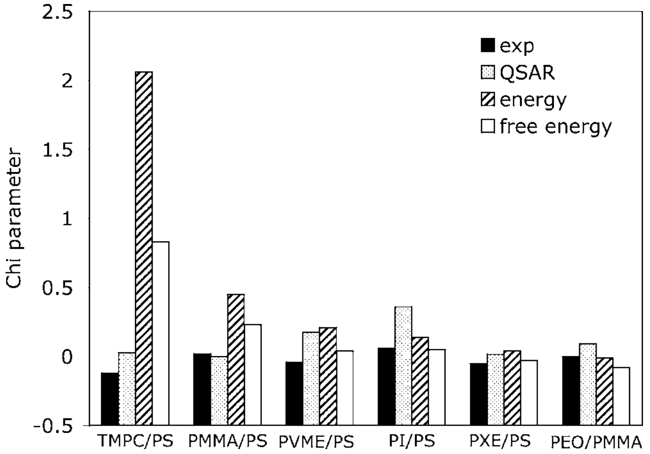

FIG. 8. Comparison of the χ-parameters: experimental, QSAR, energy, and free energy values.

the classical result of Flory. The entropic contribution, on the other hand, decreases to zero more slowly, and is fairly constant in the interval of 200–400 K. Below 200 K, the entropic contribution passes through a shallow maximum. Such behavior can lead to nonmonotonous of the total χ-parameter, which in turn can give rise to a more complex phase diagram. It is possible, for example, that the entropy leads to multiple temperatures for which χ-parameter assumes the critical value; this would correspond to a phase diagram with multiple critical points, associated with upper and lower critical temperatures. In the current application, however, the temperature at which χ assumes its critical value is unique, and the FH-theory predicts a single critical point.

B. Polymer blends

The χ-parameters for the polymer blend system calculated using the cluster method are shown in Table III. Using the separation in energetic and entropic contributions, it is clear that the entropy of mixing is positive in most cases, indicating that the mixtures are more disordered compared to the pure state. In the case of PS-PXE, this even causes a change of sign in χ, predicting that the two components are miscible in any composition at this temperature. Only for PEO/PMMA a slightly negative value for χ_s was found, which cancels the small endothermic heat found in this case.

In Fig. 8, the χ-parameters are compared to experimental and QSAR values. The experimental values are all very close to zero. Figure 8 also shows the energy contribution χ_e; in all cases the entropy correction brings the χ-parameter closer to experiment. The interaction parameters of the cluster method are of similar quality as those based on QSAR.

For the TMPC/PS blend, the current method is still far from experiment, predicting a fairly large positive interaction, as opposed to the negative value found experimentally. Also the QSAR method is unable to predict this, since it is based on the Berthelot mixing rule, which can only give positive χ values.

V. CONCLUSIONS

We have presented a simple method to estimate the interaction parameters in coarse-grained mean-field models such as the simulation method DPD and self-consistent field methods. The method is based on calculating the free energy associated with a cluster of molecules, or fragments thereof. By combining these cluster free energies for all combinations of components, a χ-parameter is obtained that includes both energy and entropy effects on mixing.

For a simple mixture of hexane and nitrobenzene around the critical temperature, the method predicts a χ-parameter reasonably close to the value a mean-field theory assumes at the critical point. A significantly higher value is obtained when excess entropy effects are not incorporated. Strikingly, a similar overestimation is also observed when calculating the χ-parameter from the energy of mixing obtained from simulating many-body mixtures explicitly using molecular dynamics. It would be interesting to establish whether this holds more generally. A potential validation route might be to evaluate free energy of mixing directly, e.g., by reversibly converting a pure state into a (dilute) mixture using thermodynamic integration. Based on the enthalpy difference in the current case, we did not pursue this, but for more dissimilar components such validation might become a feasible option.

Combining the MD-results of the pure components with an implicit mixture model in the form of Berthelot's mixing rule leads to a χ-parameter closer to the expected value. This suggests that the mixing rule effectively compensates for excess-entropy effects.

In the cases studied so far, the entropy is found to increase on mixing. This is expected as the components naturally have the best affinity in the pure state. It is conceivable, on the other hand, that the entropy may decrease on mixing if the two components have an exceptional affinity, such that the pure states are more disordered than the mixed state. When this entropy decrease is of the same order as the energy increase, this could lead to anomalous behavior, analogous to inverse freezing and melting observed experimentally.[37] This apparent anomaly in the coarse-grained space has been explained using the entropy associ-

Reuse of AIP Publishing content is subject to the terms: https://publishing.aip.org/authors/rights-and-permissions. Downloaded to IP: 129.27.168.41 On: Sun, 04 Sep 2016 07:33:05

ated with the bath degrees of freedom (or "hidden variables"), similar to the reasoning in this work.[37]

The method fits a systematic coarse-graining workflow, as sketched in Fig. 2. In such a workflow, a molecule is divided in beads of various types and the χ-parameter for each pair combination of types is calculated. We have not discussed the important question as to how this selection on coarse-graining should take place. The resolution of the model is to some extent free, albeit that the choice of bath variables puts limitations to this. For example, as the orientation is integrated out, the theory is not suitable for strong disparities in size and shape. We have also not discussed the parametrization of interaction between beads composed of more than one molecule. It is common, for example, to represent a few water molecules by a single bead. How the adsorption of a single water molecule and its environment relates to that of a water cluster, in particular if a scaling relation applies, deserves further study.

Whereas it is demonstrated that the method is efficient, systematic, and universal, it is clear that quantitative agreement with experiment cannot be universally expected. Rather we see this method as a starting point to obtain a first estimate to the interactions in coarse-grained models. Where more data are available, the resulting models can then be further fine-tuned. In particular, for strongly polarized systems, deviations are to be expected. In the current method, it is assumed that electrostatic interaction does not perturb the structure. For a system dominated by electrostatics, the interaction parameters in this situation may be better based on the van der Waals energy only, incorporating electrostatics in the mesoscale simulation using a Poisson–Boltzmann, or equivalent, model. Further study is required to validate this ansatz.

ACKNOWLEDGMENTS

I would like to thank Accelrys for funding a research sabbatical program in which part of this work was carried out. Thanks to Steffen Wilke, Struan Robertson, David Rigby, James Wescott, Stephen Todd, and Gerhard Goldbeck-Wood at Accelrys for useful discussions. I thank Joey Storer at DOW Chemicals and David England at Sanofi-Aventis for providing business cases.

APPENDIX: STATISTICAL MECHANICS OF COARSE GRAINING

In this Appendix, we will formalize the coarse graining of a microscopic system and show how, and under which conditions, this leads to a potential of mean force in Eq. (1). We only consider statistical thermodynamics; coarse-grained dynamics along similar lines was reported earlier.[38] We shall use a matrix notation, where vectors are column vectors, transposed to row vectors by T. Differential operators (or vectors thereof) are written as ∂_x.

Consider a d-dimensional system of ν/d particles with Cartesian positions ρ, conjugated momenta π, and associated masses as entries in the diagonal matrix μ, moving in a potential energy field $v(\rho)$. The Hamiltonian $\eta(\rho,\pi)$ of the system is

$$\eta = v + \tfrac{1}{2}\pi^T \mu^{-1}\pi. \tag{A1}$$

The free energy of the system, A, is defined via

$$e^{-\beta A} = \int d\tilde{\rho}\, d\tilde{\pi}\, e^{-\beta\eta} \tag{A2}$$

with $d\tilde{\rho}d\tilde{\pi}=d\rho d\pi/h^{\nu}$ a dimensionless volume element in phase space reduced by Planck's constant h. The free energy depends on the temperature T via $\beta=1/(k_BT)$ (k_B is Boltzmann's constant), the number of degrees of freedom ν, and the volume over which the positions ρ are integrated. Using Eq. (A2), the average of any property $B(\rho,\pi)$ for (β,V,ν) is calculated as

$$\langle\langle B\rangle\rangle = \int d\tilde{\rho}\, d\tilde{\pi}\, B e^{\beta(A-\eta)}. \tag{A3}$$

The double bracket notation is motivated below.

Often one is interested in properties B that are sensitive to only part $(N<\nu)$ of the degrees of freedom, (R,P), called here "mesoscale" coordinates. The integration over full phase space in Eqs. (A2) and (A3) seems therefore unnecessary, and so we ask for a reduced description of the system in terms of R and P only. We can formally derive this coarse-grained model, by introducing a transformation of phase space $(\rho,\pi)\mapsto(R,P,r,p)$ and subsequently projecting on the (R,P) subspace. The $n=\nu-N$ degrees of freedom (r,p) for which we no longer wish to account explicitly will be called bath coordinates.

In principle, R, r, P, and p can be any function of the coordinates (ρ,π). However, for (P,p) to be the canonical momenta conjugated to (R,r), the transformation must be canonical. This has the advantage that $d\rho d\pi=dRdPdrdp$ and that η is also the Hamiltonian with respect to (R,P,r,p). Consequently, the free energy, Eq. (A2), for such choice of coordinates can be written as

$$e^{-\beta A} = \int d\tilde{R}\, d\tilde{P}\left[\int d\tilde{r}\, d\tilde{p}\, e^{-\beta\eta}\right] \equiv \int d\tilde{R}\, d\tilde{P}\, e^{-\beta H}. \tag{A4}$$

As before, the tilde on the infinitesimals $dRdP$ and $drdp$ denote reduction by h raised to the number of degrees of freedom (N and n, respectively). The last line defines the energy $H(R,P;\beta,n,V)$, which is parametrized by the thermodynamic state of the bath (inverse temperature β, particle number n, and volume V). With increasing level of coarse graining, the energy H is seen to interpolate between the Hamiltonian η $(n=0)$ and the free energy A $(n=\nu)$.

We can now write the average, Eq. (A3), as

$$\langle\langle B\rangle\rangle = \int d\tilde{R}\, d\tilde{P}\, \langle B\rangle e^{\beta(A-H)}, \tag{A5}$$

where

$$\langle B\rangle = \int d\tilde{r}\, d\tilde{p}\, B e^{\beta(H-\eta)}. \tag{A6}$$

Single brackets refer to an average over the bath only; double brackets also average out the mesoscale coordinates. For properties that are not sensitive to (r,p), $\langle B\rangle\approx B$. For such properties, one expects the calculation of $\langle\langle B\rangle\rangle$ from

Reuse of AIP Publishing content is subject to the terms: https://publishing.aip.org/authors/rights-and-permissions. Downloaded to IP: 129.27.168.41 On: Sun, 04 Sep 2016 07:33:05

Eq. (A5) to be more efficient than Eq. (A3), since we have restricted the sampling to the part of phase space relevant to B.

Using the single bracket notation we have from Eqs. (A4) and (A6), $\partial_R H = \langle \partial_R \eta \rangle$ and $\partial_P H = \langle \partial_P \eta \rangle$. Since for a canonical transformation, $\partial_R \eta = -\dot{P}$ and $\partial_P \eta = \dot{R}$, we obtain the well-known result that the force/velocity derived from H corresponds to the force/velocity derived from η, on average over the bath.

We have used general canonical transformations to emphasize the general structure of coarse graining; in practice, however, one is interested in point transformations $\rho \mapsto (R, r)$ only; in other words, the coordinates (R, r) are certain functions of the microscopic positions ρ, but not of the microscopic momenta π. In this case, the microscopic momenta π are a linear combination of the mesoscale and bath momenta

$$\pi = (\partial_\rho R^T) P + (\partial_\rho r^T) p \equiv J_R P + J_r p.$$

Substituting in Eq. (A1) leads to

$$\eta = v + \tfrac{1}{2} P^T J_R^T \mu^{-1} J_R P + P^T J_R^T \mu^{-1} J_r p + \tfrac{1}{2} p^T J_r^T \mu^{-1} J_r p$$
$$\equiv v + \tfrac{1}{2} P^T X P + P^T Y p + \tfrac{1}{2} p^T Z p,$$

where the last line defines the metric matrices X, Y, and Z.

Substituting in Eq. (A4) and integrating over the bath momenta lead to

$$e^{-\beta H} = \int d\tilde{r} e^{-\beta(v + 1/2 P^T X P)} \int d\tilde{p} e^{-\beta(P^T Y p + 1/2 p^T Z p)}$$
$$= \int d\tilde{r} e^{-\beta(v + 1/2 P^T W P)}, \tag{A7}$$

where $W = X + Y Z^{-1} Y^T$. We have used the positive definity of βZ. The infinitesimal dr is reduced by the length unit $\Lambda = h |Z|^{1/n} / \sqrt{2\pi/\beta}$, which may depend on r through Z.

The energy H in Eq. (A7) has not yet the structure of the Hamiltonian η in Eq. (A1), where the kinetic and potential energy terms are clearly separated. It is clear, however, that if $Y = 0$, and X nor Z depends on the bath positions r, Eq. (A7) factorizes as

$$H = -\frac{1}{\beta} \ln \left[\int d\tilde{r} e^{-\beta v} \right] + \frac{1}{2} P^T X P \equiv U + \frac{1}{2} P^T X P, \tag{A8}$$

which does have the same structure as Eq. (A1), but differs in the meaning of the potential part: The potential v represents an energy as a function of the positions ρ only. The potential U in Eqs. (A8) and (1) represents a free energy as a function of the positions R as well as the thermodynamic state of the bath. Since the R dependence of H is contained in U, the potential U is the potential of the mean force $-\langle \partial_R v \rangle$, where the average is over the bath positions r.

The condition that X and Z, and hence J_R and J_r, are independent of r, means that the transformations are linear, which is typically the case. For example, where R stands for the center-of-mass positions of molecules, and r are the particle positions relative to the center-of-mass. Moreover, as has been shown,[39] it is possible to construct r such that $Y = 0$.

[1] G. De Fabritiis, R. Delgado-Buscalioni, and P. V. Coveney, Phys. Rev. Lett. **97**, 134501 (2006).
[2] D. N. Theodorou, Comput. Phys. Commun. **169**, 82 (2005).
[3] J. T. Wescott, P. Kung, and A. Maiti, Appl. Phys. Lett. **90**, 033116 (2007).
[4] A. Maiti, J. T. Wescott, G. Goldbeck-Wood, Int. J. Nanotechnol. **2**, 198 (2005).
[5] M. J. Buehler and T. Ackbarow, Mater. Today **10**, 46 (2007).
[6] R. L. C. Akkermans and P. B. Warren, Philos. Trans. R. Soc. London, Ser. A **362**, 1783 (2004).
[7] P. J. Flory, J. Chem. Phys. **9**, 660 (1941); M. L. Huggins, *ibid.* **9**, 440 (1941).
[8] J. M. H. M. Scheutjens and G. J. Fleer, J. Phys. Chem. **83**, 1619 (1979).
[9] H. D. Ceniceros and G. H. Fredrickson, Multiscale Model. Simul. **2**, 452 (2004).
[10] J. G. E. M. Fraaije, A. V. Zvelindovsky, and G. J. A. Sevink, Mol. Simul. **30**, 225 (2004).
[11] P. J. Hoogerbrugge and J. M. V. A. Koelman, Europhys. Lett. **19**, 155 (1992).
[12] R. D. Groot and P. B. Warren, J. Chem. Phys. **107**, 4423 (1997).
[13] R. D. Groot and K. L. Rabone, Biophys. J. **81**, 725 (2001).
[14] F. H. Case and J. D. Honeycutt, Trends Polym. Sci. **2**, 259 (1994).
[15] A. Maiti and S. McGrother, J. Chem. Phys. **120**, 1594 (2004).
[16] J. Rowlinson, *Liquids and Liquid Mixtures* (Springer, New York, 1995).
[17] R. L. McGreevy, J. Phys.: Condens. Matter **13**, R887 (2001).
[18] A. Tarantola, *Inverse Problem Theory and Methods for Model Parameter Estimation* (SIAM, Philadelphia, PA, 2004).
[19] R. L. C. Akkermans and W. J. Briels, J. Chem. Phys. **115**, 6210 (2001).
[20] D. Frenkel and B. Smit, *Understanding Molecular Simulation*, 2nd ed., (Academic, New York, 2002).
[21] F. Müller-Plathe, ChemPhysChem **3**, 754 (2002).
[22] R. L. C. Akkermans and W. J. Briels, J. Chem. Phys. **113**, 6409 (2000).
[23] J.-W. Chu, S. Izvekov, and G. A. Voth, Mol. Simul. **32**, 211 (2006).
[24] A. P. Lyubartsev, M. Karttunen, I. Vattulainen, and A. Laaksonen, Soft Mater. **1**, 121 (2002).
[25] S. L. Njo, W. F. van Gunsteren, and F. Müller-Plathe, J. Chem. Phys. **102**, 6199 (1995).
[26] P. B. Warren, J. Phys.: Condens. Matter **15**, S3467 (2003).
[27] I. Paganobarraga and D. Frenkel, Mol. Simul. **25**, 167 (2000).
[28] F. Ercolessi and J. B. Adams, Europhys. Lett. **26**, 583 (1994).
[29] C. F. Fan, B. D. Olafson, M. Blanco, and S. L. Hsu, Macromolecules **25**, 3667 (1992); M. Blanco, J. Comput. Chem. **12**, 237 (1991).
[30] E. Helfand, Macromolecules **25**, 1676 (1992).
[31] J. Bicerano, *Prediction of Polymer Properties*, (Dekker, Dordrecht, 2002).
[32] H. Sun, J. Phys. Chem. B **102**, 7338 (1998).
[33] AMORPHOUS CELL, COMPASS, DPD, FORCITE, and SYNTHIA are modules in Materials Studio®, the materials science modeling and simulation package of Accelrys Inc., San Diego. For further information, see http://accelrys.com/products/materials-studio
[34] E. Dufresne, T. Nurushev, R. Clarke, and S. Dierker, Phys. Rev. E **65**, 061507 (2002).
[35] J. S. Chikos and W. E. Acree, Jr, J. Phys. Chem. Ref. Data **32**, 519 (2003).
[36] A. F. M. Barton, *Handbook of Solubility Parameters and Other Cohesive Compounds*, 2nd ed., (CRC, Boca Raton, FL, 1991).
[37] N. Schupper and N. M. Shnerb, Phys. Rev. E **72**, 046107 (2005).
[38] R. L. C. Akkermans, Lect. Notes Comput. Sci. **49**, 155 (2006).
[39] E. Darve and A. Pohorille, J. Chem. Phys. **115**, 9169 (2001).

Reuse of AIP Publishing content is subject to the terms: https://publishing.aip.org/authors/rights-and-permissions. Downloaded to IP: 129.27.168.41 On: Sun, 04 Sep 2016 07:33:05
