# Supplementary material for: Cluster-Based Thermodynamics of Interacting Dice in a Lattice
Source: Entropy (Basel). 2020 Oct 1;22(10):1111. doi: 10.3390/e22101111 (PMC7597232; doi:10.3390/e22101111)
Supplement: Supplementary file 1 [file entropy-22-01111-s001.zip › Supplementary Files/Literature/Freed 2000 Lattice Cluster Theory for Pedestrians - Models for Random Copolymer Blends.pdf]

Macromol. Symp. ***149,*** *11–16 (2000)*

Lattice Cluster Theory for Pedestrians: Models for Random Copolymer Blends

Karl F. Freed and Jacek Dudowicz

James Franck Institute and Department of Chemistry, University of Chicago, 5640 S. Ellis, Chicago, IL 60637, USA

SUMMARY A simplifying version of the lattice cluster theory is formulated for binary random copolymer A_xB_{1-x}/A_yB_{1-y} blends. The underlying model is based on united atom structures for the individual monomers of A and B species. Applications of the theory to saturated poly(butadiene) blends display only semiquantitative agreement with experiment. We discuss possible routes to theoretical improvements and experiments that would be helpful in testing theory.

Introduction

The overwhelming majority of thermodynamic descriptions for multicomponent polymer systems are based on Flory-Huggins (FH) theory[1] and various embellishments thereof. These theories have proven to be extremely useful in correlating data from measurements of different properties, but the theories contain several deficiencies that represent serious impediments to their predictive abilities. For example, FH theory[1] for a binary polymer blend employs a single adjustable parameter, the dimensionless Flory interaction parameter χ, *which emerges from the theory* as inversely proportional to temperature, independent of composition, molecular weight, pressure, and chain architecture. The observed χ, however, frequently depends on composition, sometimes varies with molecular weights, and exhibits a dependence on pressure (as predicted by us prior to its experimental observation). Moreover, the empirical χ generally contains a temperature independent portion (often called the entropic χ, χ_S.) In addition, FH theory[1] cannot distinguish between block, random, and alternating copolymers of a given composition. These myriad strong contradictions between theory and experiment logically imply that FH theory is *wrong (i.e., grossly inadequate to explain general observations* for χ.) Thus, treating χ as an phenomenological parameter, with all the observed variations, *technically* represents pure empiricism but not use of FH theory, *per se*. While this empiricism is perfectly reasonable, its apparent successes cannot rectify the lack of theoretical underpinnings and the serious impediments posed by these strong deficiencies of FH theory.

The above mentioned limitations to FH theory are merely the tip of the iceberg when questions are posed concerning the molecular design of new materials. FH theory specifies that monomers of each species and solvent molecules (if present) all occupy single lattice sites (i.e., occupy the same volumes), but this assumption grossly ignores the significant differences

© WILEY-VCH Verlag GmbH, D-69469 Weinheim, 2000 CCC 1022-1360/00/$ 17.50+.50/0

in monomer sizes and shapes and their influence on packing and nonrandom mixing. Thus, one ingredient in a molecular based theory[2] for the statistical thermodynamics of multicomponent polymer systems must involve a more realistic description of the individual monomers and their distinct chemical structures. Ultimately, a molecular theory must devise sets of interaction parameters that are transferable between various systems.

Some empirical progress towards developing transferable interaction parameters has emerged from the use of solubility parameter theory[3] to analyze data for χ of binary polyolefin blends as obtained from small angle neutron scattering experiments. While this approach has the strong virtue of representing χ in terms of single component solubility parameters, the theory is only successful in explaining roughly 70% of the data. Most disturbingly, the failures of solubility parameter theory[3] become more prevalent for binary blends of two homopolymer polymer blends, with the theory working better for the seemingly much more complex random copolymer systems. Solubility parameter theory also cannot explain the existence of lower critical solution temperature (LCST) phase diagrams observed, for instance, for binary blends of poly(isobutylene) (PIB) with other polyolefins.

The Lattice Cluster Theory

We have developed the lattice cluster theory[2] (LCT) to provide a molecular basis for understanding and predicting diverse thermodynamic properties of polymer blends. The theory involves two fundamental advances beyond FH theory. Firstly, the LCT employs an extended lattice model where monomers are endowed with molecular structures, as illustrated in Fig. 1 which depicts united atom structures for polyolefin monomers. Individual CH_n groups, n = 0 - 3, are taken as occupying single lattice sites, and the bonds between united atom groups correspond to the CC bonds in the actual molecules.

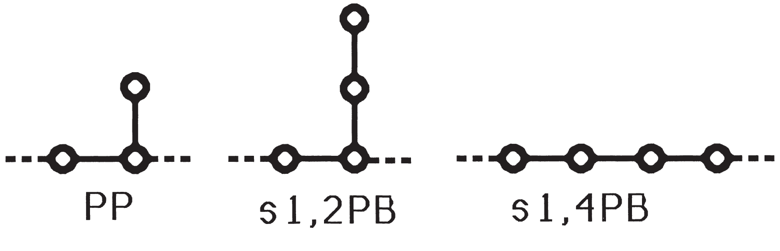

Fig. 1: United atom models for monomers of poly(propylene) (PP) and for the 1,2 and 1,4 additions in saturated poly(butadiene) (sPB). Circles denote united atom groups, and dotted lines indicate bonds between backbone units.

The second major advance in the LCT emerges from a vastly improved solution of the lattice model with structured monomer chains, a solution describing the thermodynamic consequences of differences between the monomer structures and between different chain architectures. LCT descriptions of polymer blends exhibit excellent agreement with general physical trends produced by off-lattice theories. More importantly, the LCT has contributed[2] to many advances in our understanding of polymer blend thermodynamics, including

a) an explanation[4] for the molecular origins of the previously enigmatic entropic contribution to the Flory interaction parameter χ;

b) a demonstration of a strong influence[5] of monomer molecular structures on blend miscibilities and other thermodynamic properties;

c) an explanation[5] for the observed composition (and molecular weight) dependence of χ and a prediction of its pressure dependence, which was *subsequently verified* by experiments;

d) a prediction[6] (*subsequently verified experimentally*[7]) that certain block copolymer systems order upon *heating*;

e) a molecular explanation for the origins of sequence dependent thermodynamic properties in random copolymer systems;

f) the explanation of LCST phase behavior of incompressible binary blends as emerging from the entropic χ parameter which is easily computed from the united atom monomer structures for both blend components;[8]

g) a description of the non-random mixing in polymer blends and its influence on the miscibility of polyolefin blends;[9]

h) the explanation of general trends for the pressure dependence of blend phase diagrams (UCST blends are generally destabilized by increased pressure, while LCST blends are stabilized);

i) a description of the synergistic roles[10] of interaction, stiffness, and monomer structural differences between components in controlling the phase behavior of polymer blends;

j) the prediction of a remarkable correlation[10] of the blend critical temperature with disparities in monomer structure and chain stiffness between the blend components;

k) a quantitative extension of the old (very vague) Guggenheim[11] concept of surface fractions to polymer chains with structured monomers;[8]

l) the prediction of a rich variety of phase behaviors for random copolymer/homopolymer blends; and

m) the development of a simplifying (readily usable by experimentallists) “pedestrian” limit of the LCT[8] that has successfully explained anomalous mixing in binary homopolymer blends and in binary mixture of ethylene-co-norbornene random copolymers.[12] A similar limit is applied here to blends of saturated poly(butadienes).

Simple LCT Model for Random Copolymer Blends

Among many scientifically and technologically important polymer systems, random copolymers play a special role because of their ability to promote the miscibility of otherwise immiscible components and because they often may be produced at relatively low cost. Theoretical descriptions of blends containing random copolymers are generally based on extensions[13] of FH theory to these systems and therefore suffer from the severe deficiencies outlined above. As a prelude to the very nontrivial task of extending the full LCT to binary random copolymer blends, it is essential to test simplified limits of the LCT against experiment in order to devise approximations that are readily usable by experimentallists and to provide guidelines for developing the full LCT theory of these important systems.

From the standpoint of theoretical modeling, the simplest blends of two random copolymers are mixtures of copolymers formed from the same pair of monomer species A and B but having different compositions x and y. The simplicity in modeling these systems stems from the minimal number of required interaction parameters. Perhaps, the most extensively studied binary A_xB_{1-x}/A_yB_{1-y} system is a mixture of two saturated poly(butadiene) (sPB),[14,15] where the different "monomer" species A and B emerge from 1,2 and 1,4 additions respectively. The interaction parameter χ for these systems have been determined from fits to small angle neutron scattering (SANS) intensities and to nuclear reaction analysis data for a range of different (x,y), temperatures, and compositions. Both sets of experiments[14,15] use the same samples, where one component (A_xB_{1-x}) is hydrogenated, while the other component (A_yB_{1-y}) is partially deuterated, implying a distribution of deuterium content among the monomers. While the individual χ values from both experiments agree to roughly ±25%, there are strong discrepancies when comparing the temperature dependence in the usual form of $\chi = \chi_s + \chi_h/T$. The SANS data yield very large negative χ_s, while, in contrast, the nuclear reaction analysis yields a nearly vanishing $\chi_s << \chi_h$. This discrepancy may arise because of the variations in deuterium content among the individual monomers in the partially deuterated samples, a feature that may affect the interpretation for both types of experiments. Thus, our goal here lies only in probing general trends.

We model the random copolymer blend using the following simplifying assumptions: 1) The blend is taken as incompressible. 2) The individual monomers of A and B are represented by united atom models as in Fig. 1. 3) The self-interaction energies ε_{AA} and ε_{BB} are assigned to each of the united atom groups in the 1,2 and 1,4 addition monomers, respectively. 4) The heterocontact interaction ε_{AB} is approximated as $\varepsilon_{AB} = (\varepsilon_{AA}\,\varepsilon_{BB})^{1/2}$. 5) The χ_s is computed from the random copolymer LCT for the long chain incompressible limit. 6) χ_h is evaluated from the extension of FH to united atom models of random copolymer chains. 7) Deuteration scales each interaction energy $\varepsilon_{\alpha\beta}$ by a factor of γ^2 when both interacting species are

deuterated and by γ when only one is deuterated. Assessing the validity of these assumptions serves to guide further theoretical advances.

Given the above approximations, the monomer-monomer effective interaction parameter is

$$\chi = 2z\{(1-\gamma)[y(\varepsilon_{AA})^{1/2} + (1-y)(\varepsilon_{BB})^{1/2} + (x-y)[(\varepsilon_{AA})^{1/2} - (\varepsilon_{BB})^{1/2}]\}^2 + (x-y)^2/4z^2,$$

where the lattice coordination number z is taken as z = 6. The energies ε_{AA} and ε_{BB} and the polarizability parameter γ are least-squares fit to SANS data[14] for six binary blends (24 data points) with (x,y) = (0.52,0.35), (0.35,0.52), (0.38,0.25), (0.25,0.38), (0.25,0.08), and (0.08,025). The fit yields ε_{AA} = 238 K, ε_{BB} = 241 K, and γ^2 = 0.9901, with a standard deviation of 1.6×10^{-4} which represents semiquantitative agreement. In addition, the theory describes well the deuterium swap effect, i.e., a larger χ is obtained when the more branched polyolefin component is deuterated, e.g., χ for (x,y) = (0.38,0.25) exceeds χ for (0.25,0.38), as exhibited in Fig. 2 which illustrates the comparison between theory and experiment.

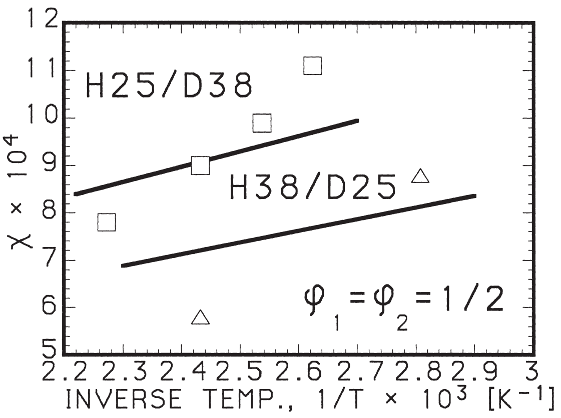

Fig. 2: Comparison of simplified LCT with experiment[14] for the two sPB blends H25/D38 ≡ (0.38,0.25) and H38/D25 ≡ (0.25,0.38).

Figure 2 evidences some deviations between theory and experiment. A large portion of this disagreement stems from the large negative χ_s in the SANS data,[14] whereas the calculated LCT χ_s is positive. Although all the χ_s from the nuclear reaction analysis data are almost zero, the theory is unable to provide a better fit. Thus, we consider possible reasons for these discrepancies. The first reason emerges from the preparation of the experimental samples because the degree of deuteration varies among the monomers, possibly accounting for excess scattering that is not considered in the experimental analysis of the SANS data. Hence, it would be desirable to perform new experiments with fully deuterated saturated polybutadiene

chains to eliminate this possible source of disagreement. On the other hand, the theory invokes a number of simplifying assumptions which also may be responsible for the lack of quantitative agreement. Some tests have been made by lifting individual approximations as follows: First of all, introducing chain semiflexibility improves agreement with experiment but does not generate χ_s as negative as in the SANS data.[14] Based on LCT computations for homopolymer blends, lifting the incompressibility assumption could also produce a small negative contribution to χ_s. Another potential source of errors may arise from using the same interaction energies for all united atom groups in the saturated 1,2 and 1,4 units. Substantial off-lattice modeling of alkane systems demonstrates the large differences, for instance, between CH_3-CH_3 and CH_2-CH_2 Lennard-Jones interaction parameters, differences that swamp any changes in these interactions upon deuteration. We surmise that a precise description of very weakly interacting systems, such as the sPB blends, may require explicitly treating these different group interactions. This type of treatment may also be necessary to explain quantitatively the composition dependence of the interaction parameter for isotopic polyolefin blends.

Acknowledgment

This research is supported, in part, by Grant DAAG55-97-1-0162 from ARO.

References

[1]. P. J. Flory, *Principles of Polymer Chemistry* (Cornell U. Press, Ithaca, NY, 1953).
[2]. K. F. Freed and J. Dudowicz, *Trends Polym. Sci.* **3**, 248 (1995) and references therein.
[3]. W. W. Graessley *et al.*, *Macromolecules* **28**, 1260 (1995).
4 . K. F. Freed and A. I. Pesci, *J. Chem. Phys.* **87**, 7342 (1987).
5 . J. Dudowicz and K. F. Freed, *Macromolecules*, **28**, 6625 (1995); **24**, 5112 (1991).
6 . J. Dudowicz and K. F. Freed, *Macromolecules*, **26**, 213 (1993).
7 . T. P. Russell, T. E. Karis, Y. Gallot, and A. M. Mayes, *Nature* **368**, 729 (1994); T. E. Karis *et al.*, *Macromolecules* **28**, 1129 (1995).
[8]. K. F. Freed and J. Dudowicz, *Macromolecules* **31**, 6681 (1998).
[9]. K. F. Freed and J. Dudowicz, *Macromolecules* **29**, 625 (1996).
[10]. J. Dudowicz and K. F. Freed, *Macromolecules* **29**, 8960 (1996); K. W. Foreman and K. F. Freed, *Macromolecules* **30**, 7295 (1997).
11. E. Guggenheim, *Mixtures* (Oxford University Press, Oxford, 1952).
[12]. C. Delfolie, L. C. Dickinson, K. F. Freed, J. Dudowicz, W. J. MacKnight, *Macromolecules* (submitted).
[13]. G. ten Brinke, F. E. Karasz, and W. J. MacKnight, *Macromolecules* **16**, 1827 (1983).
[14]. W. W. Graessley *et al.*, *Macromolecules* **27**, 2574 (1994).
[15]. F. Scheffold *et al.*, *J. Chem. Phys.* **104**, 8786 (1996).
