# Supplementary material for: Cluster-Based Thermodynamics of Interacting Dice in a Lattice
Source: Entropy (Basel). 2020 Oct 1;22(10):1111. doi: 10.3390/e22101111 (PMC7597232; doi:10.3390/e22101111)
Supplement: Supplementary file 1 [file entropy-22-01111-s001.zip › Supplementary Files/Literature/LiteratureSearchOverview.rtf]

Searches in 'Entropy' (Title / Keyword)  concerning the current state of the research field
dice
molecule
liquid
quasi-chemical
interaction
coupling
cooperative
lattice
many-body
force-field
activity coefficient
Potts
Markov
