# Supplementary figures and images for: Cluster-Based Thermodynamics of Interacting Dice in a Lattice
Source: Entropy (Basel). 2020 Oct 1;22(10):1111. doi: 10.3390/e22101111 (PMC7597232; doi:10.3390/e22101111)

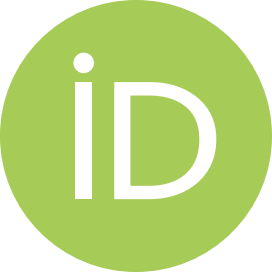

Supplement: Supplementary file 1 [file entropy-22-01111-s001.zip › Supplementary Files/Definitions/logo-orcid-eps-converted-to.pdf]

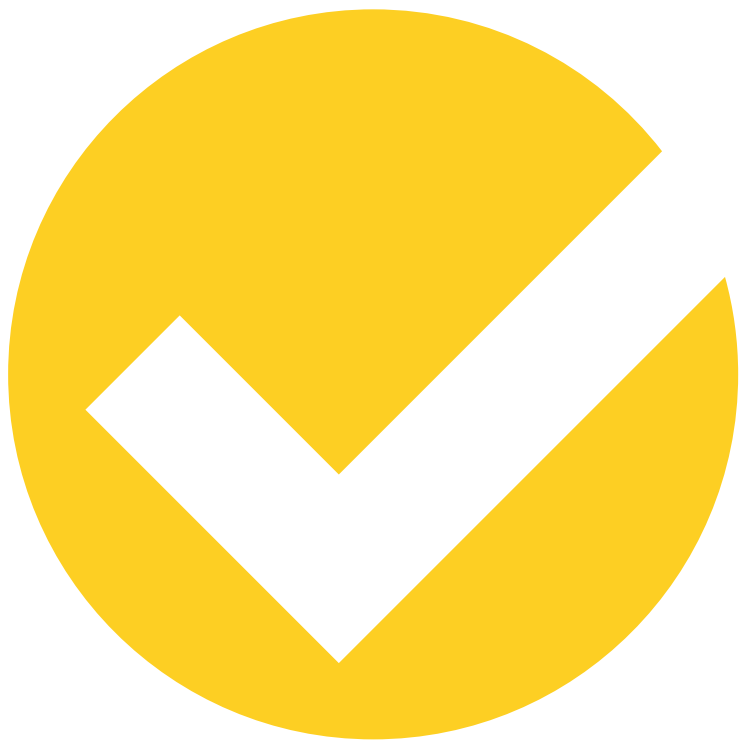

check for  
updates

Supplement: Supplementary file 1 [file entropy-22-01111-s001.zip › Supplementary Files/Definitions/logo-updates.pdf]

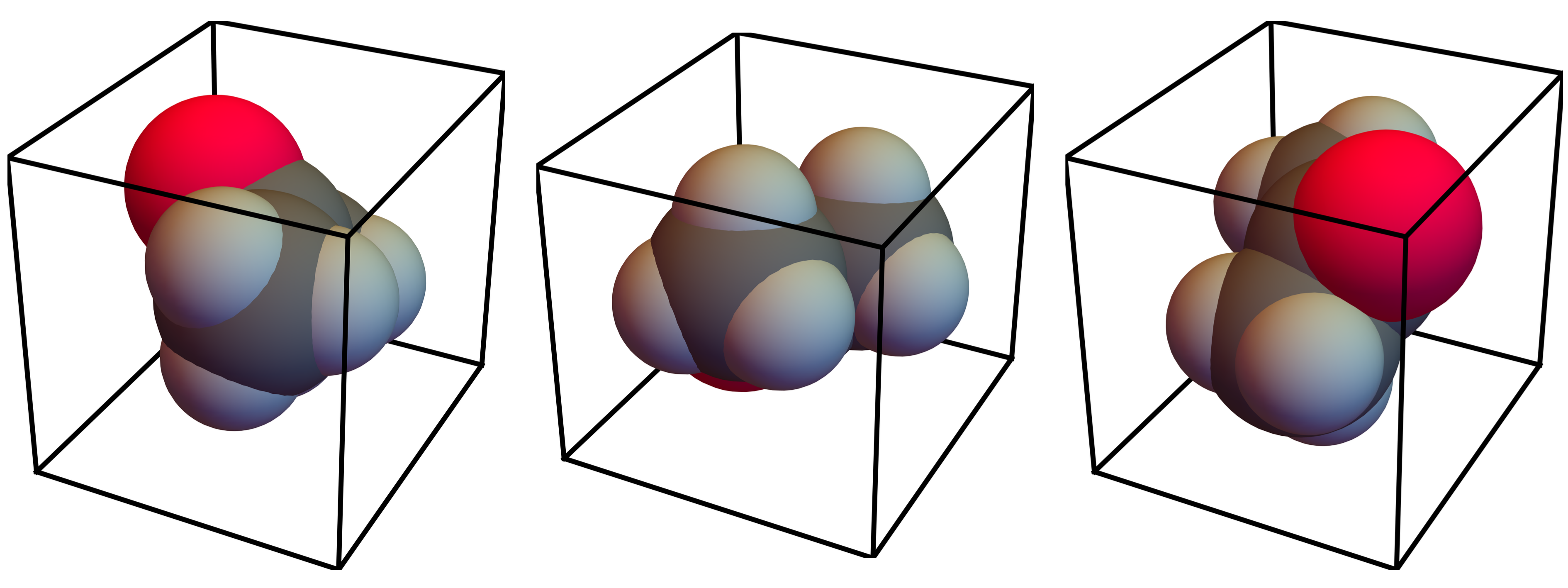

Supplement: Supplementary file 1 [file entropy-22-01111-s001.zip › Supplementary Files/Graphics/AcetoneOrientations.png]

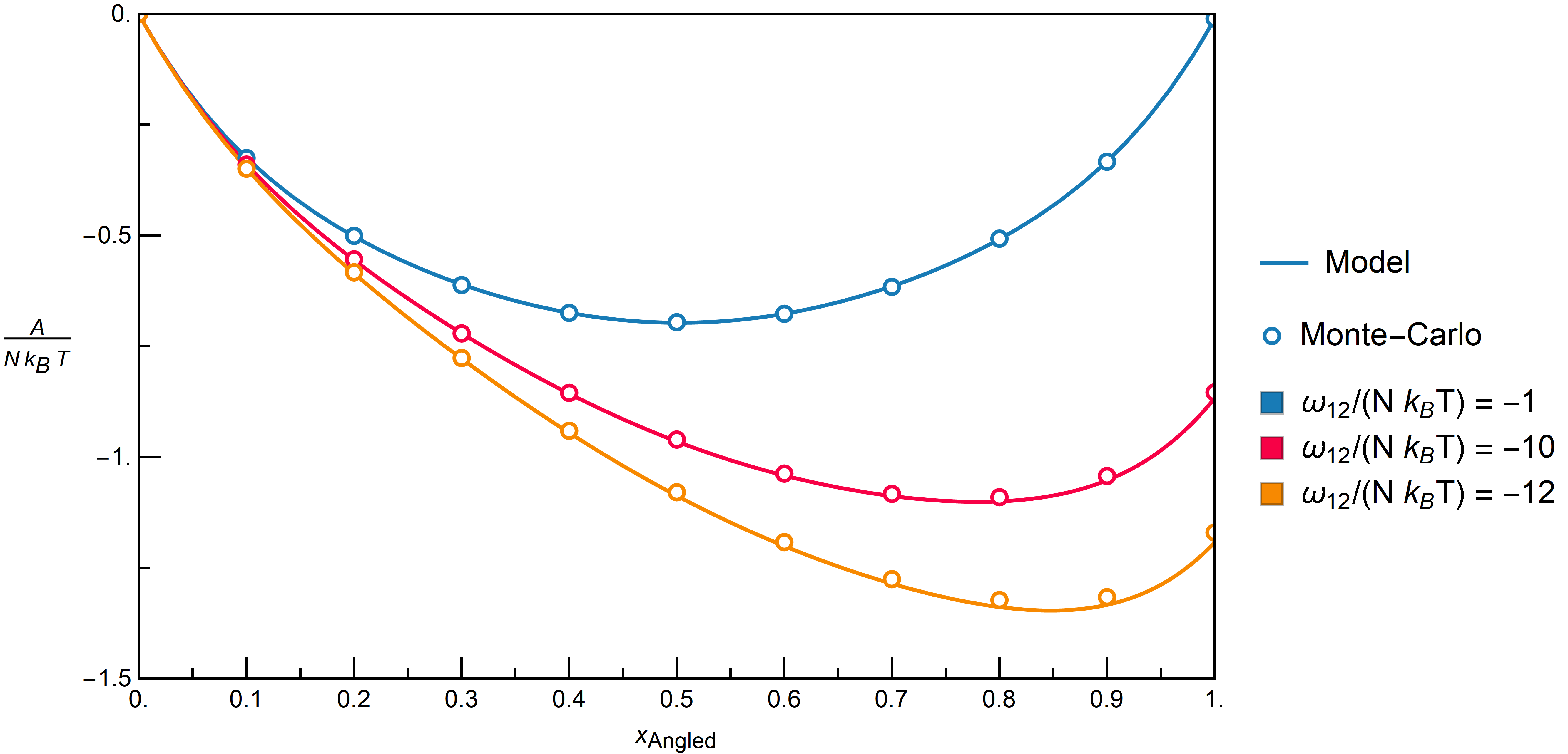

Supplement: Supplementary file 1 [file entropy-22-01111-s001.zip › Supplementary Files/Graphics/AngledInert.png]

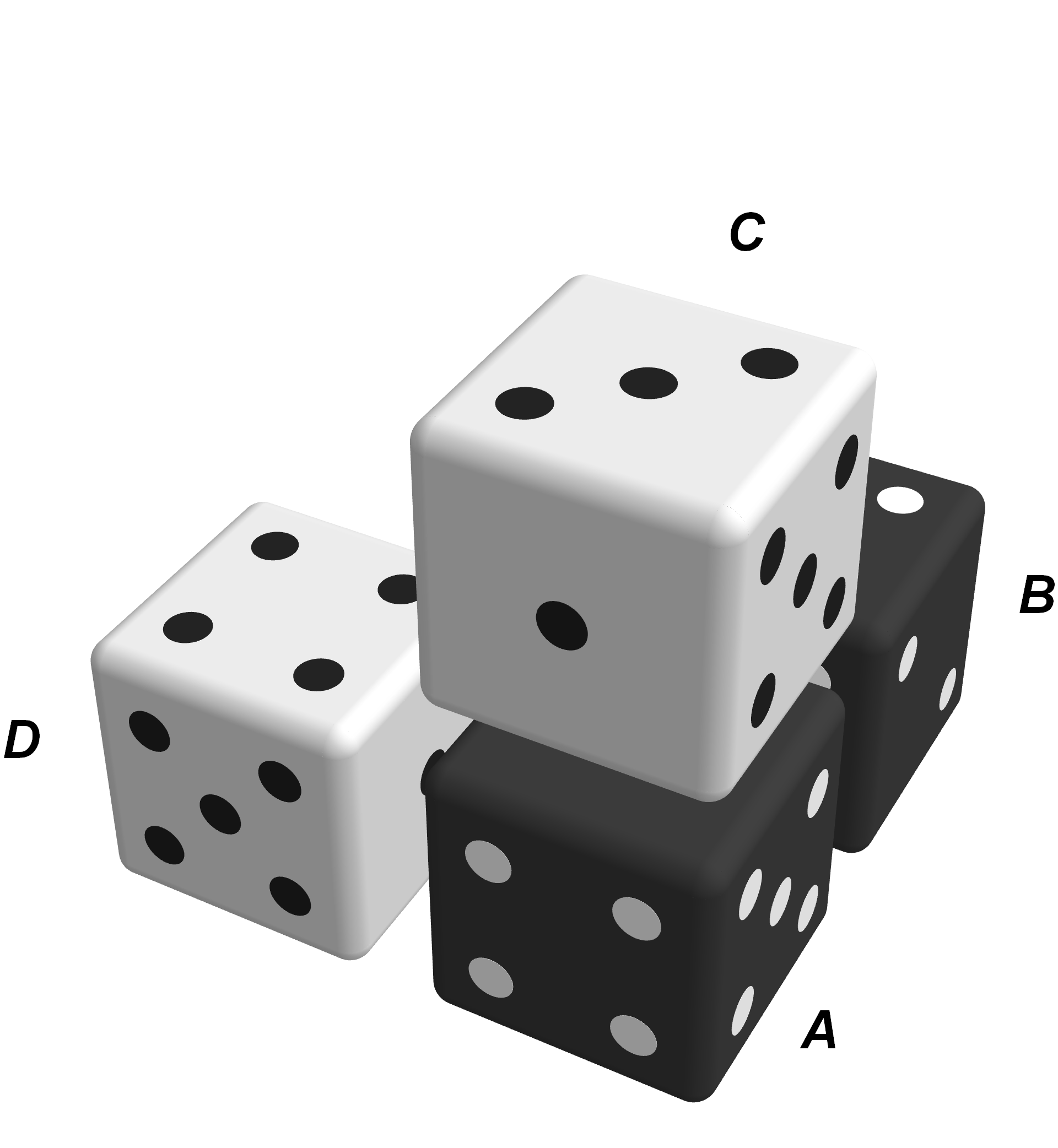

Supplement: Supplementary file 1 [file entropy-22-01111-s001.zip › Supplementary Files/Graphics/ClusterConstruction.png]

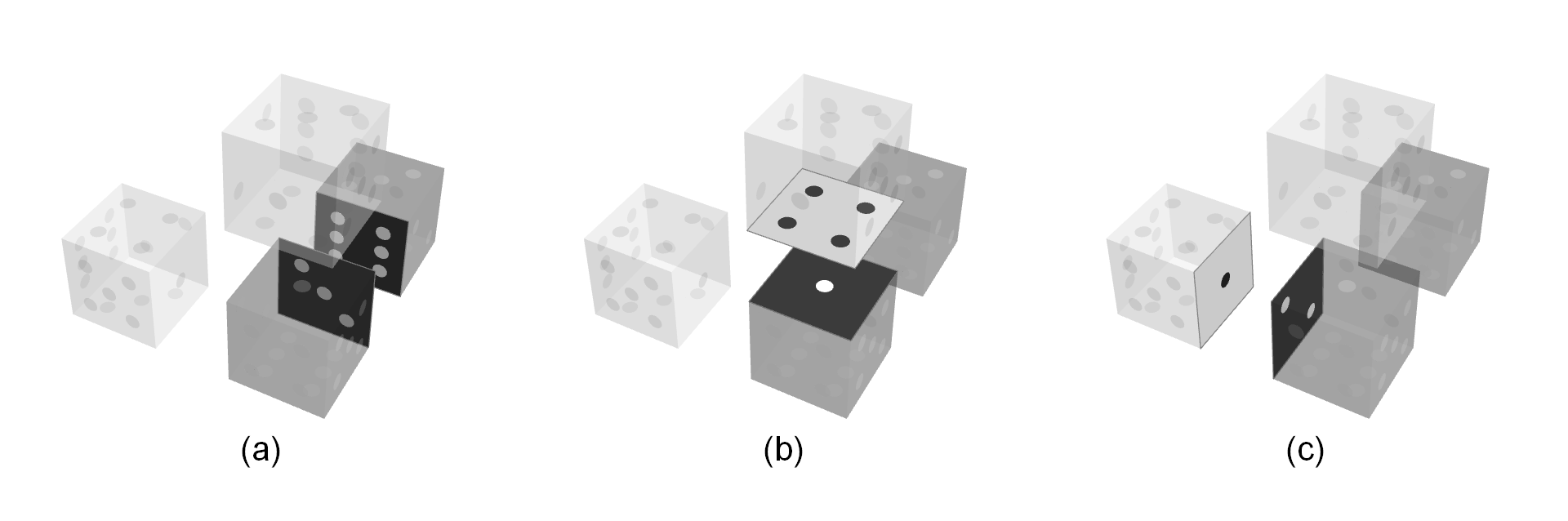

Supplement: Supplementary file 1 [file entropy-22-01111-s001.zip › Supplementary Files/Graphics/ClusterContacts.png]

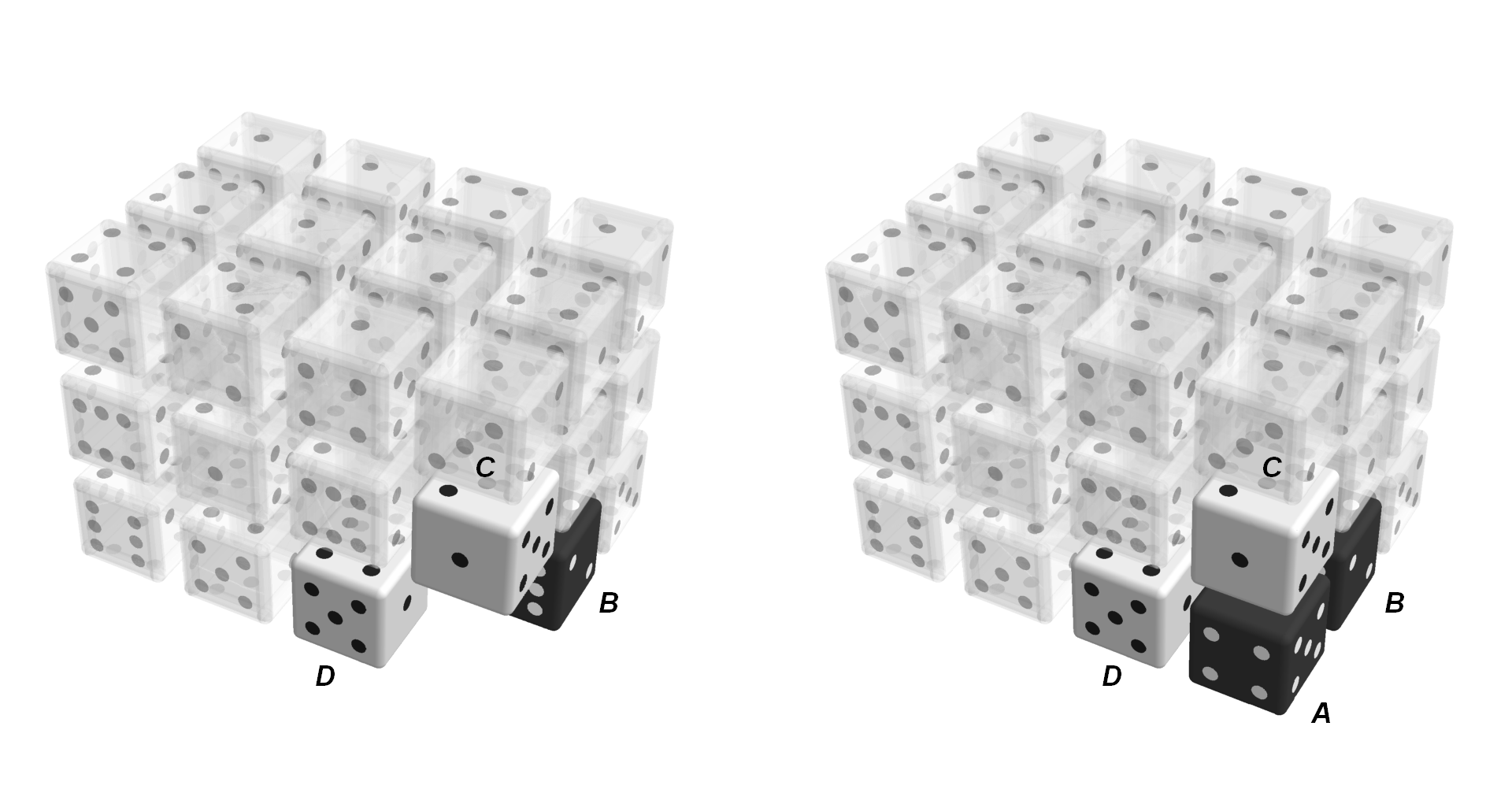

Supplement: Supplementary file 1 [file entropy-22-01111-s001.zip › Supplementary Files/Graphics/ClusterInsertion.png]

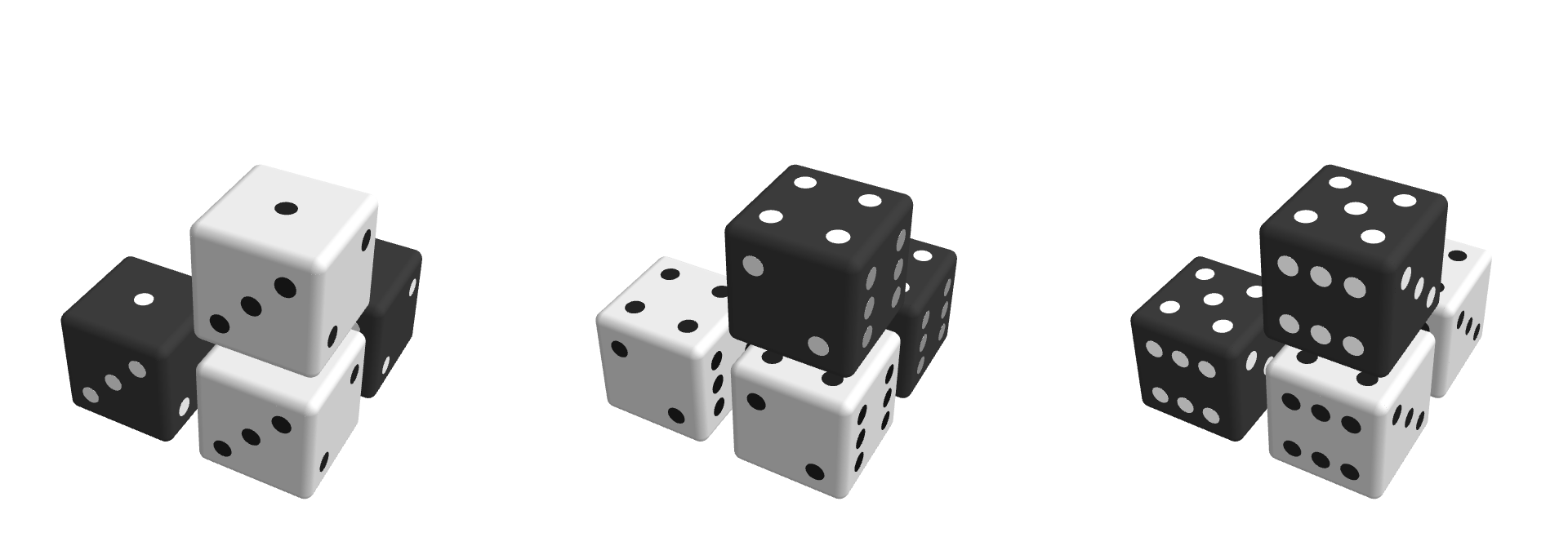

Supplement: Supplementary file 1 [file entropy-22-01111-s001.zip › Supplementary Files/Graphics/ClusterIsotropy.png]

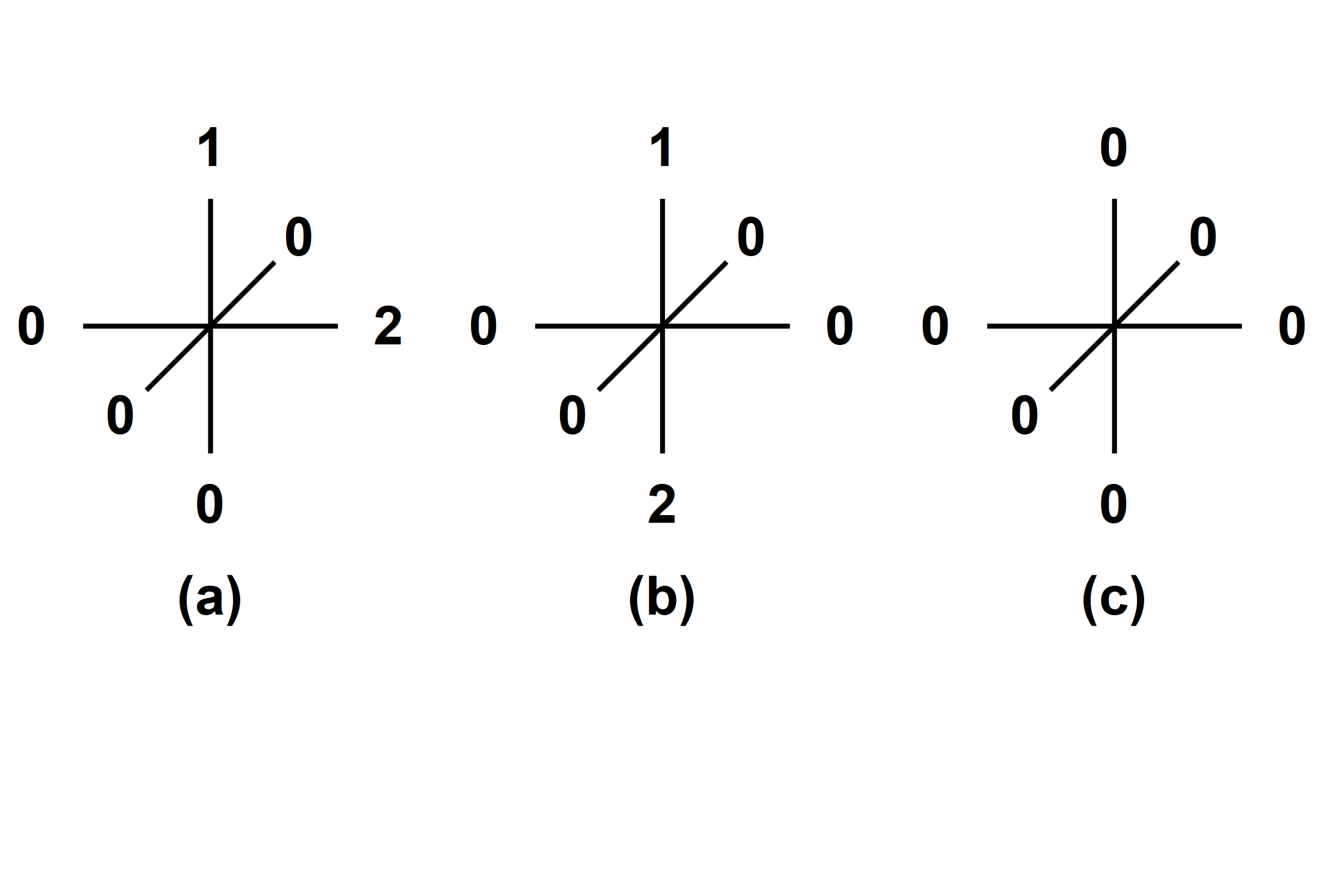

Supplement: Supplementary file 1 [file entropy-22-01111-s001.zip › Supplementary Files/Graphics/ComponentGraphic.png]

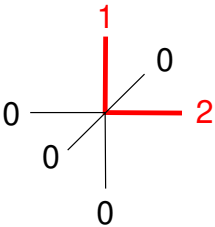

(a)

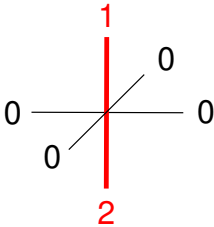

(b)

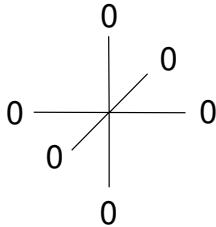

(c)

Supplement: Supplementary file 1 [file entropy-22-01111-s001.zip › Supplementary Files/Graphics/ComponentGraphicNew.pdf]

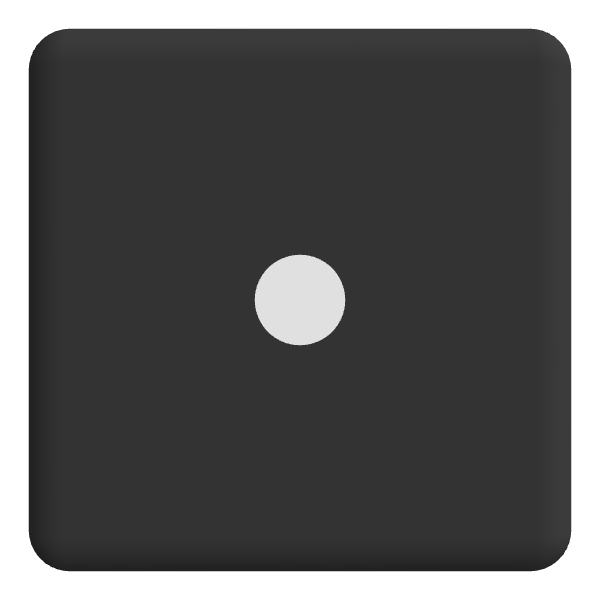

Supplement: Supplementary file 1 [file entropy-22-01111-s001.zip › Supplementary Files/Graphics/DieFaceBlack1.png]

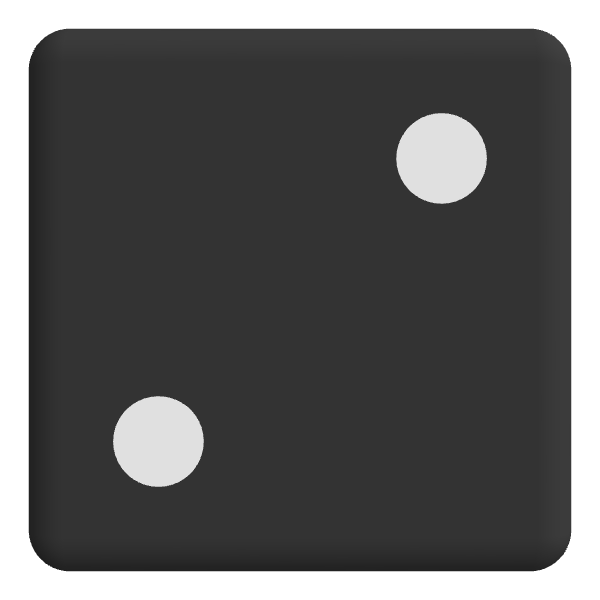

Supplement: Supplementary file 1 [file entropy-22-01111-s001.zip › Supplementary Files/Graphics/DieFaceBlack2.png]

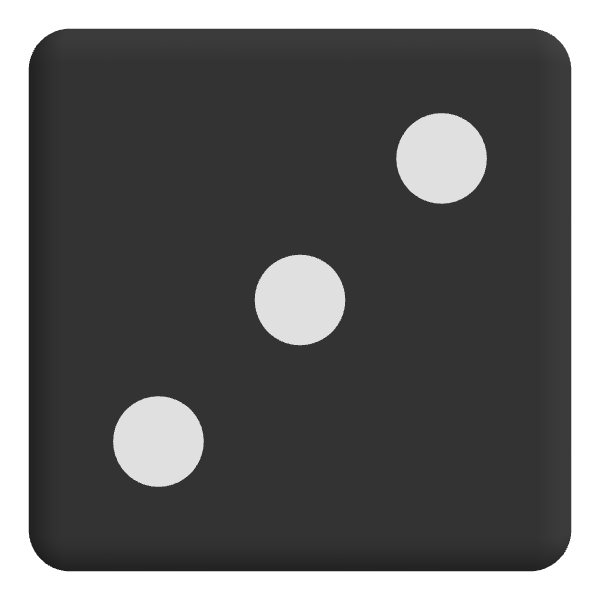

Supplement: Supplementary file 1 [file entropy-22-01111-s001.zip › Supplementary Files/Graphics/DieFaceBlack3.png]

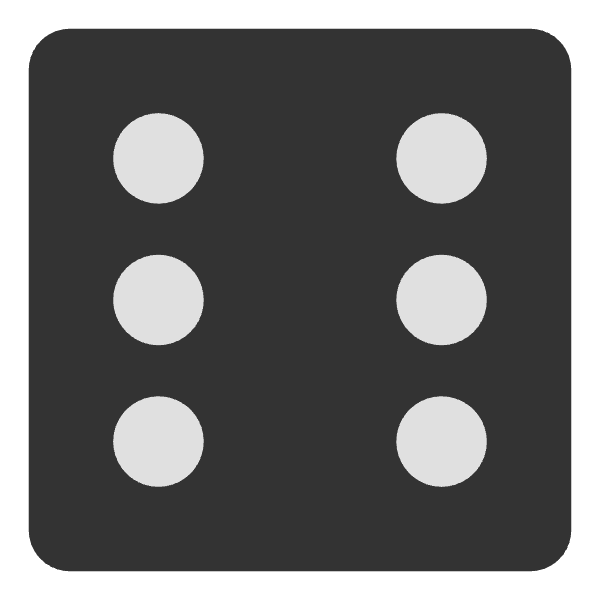

Supplement: Supplementary file 1 [file entropy-22-01111-s001.zip › Supplementary Files/Graphics/DieFaceBlack6.png]

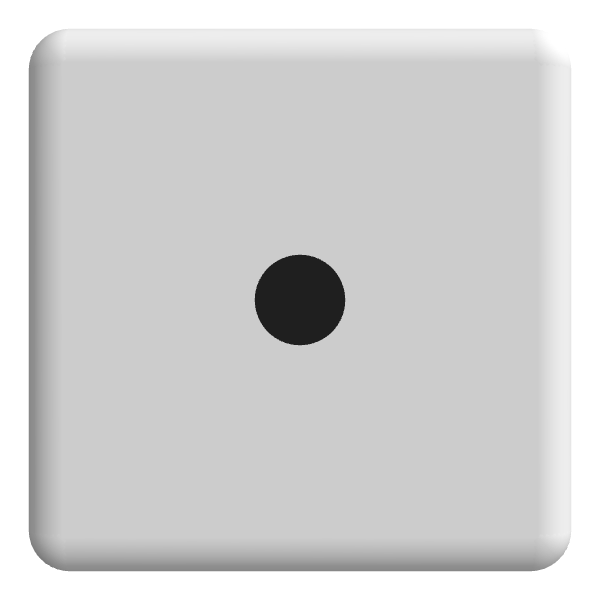

Supplement: Supplementary file 1 [file entropy-22-01111-s001.zip › Supplementary Files/Graphics/DieFaceWhite1.png]

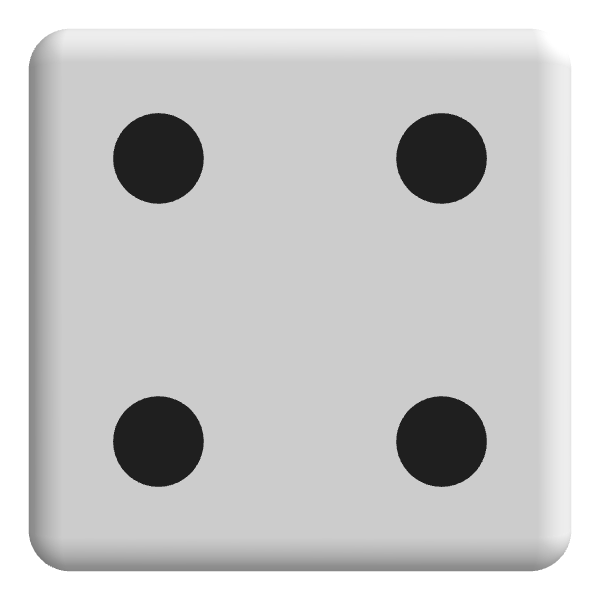

Supplement: Supplementary file 1 [file entropy-22-01111-s001.zip › Supplementary Files/Graphics/DieFaceWhite4.png]

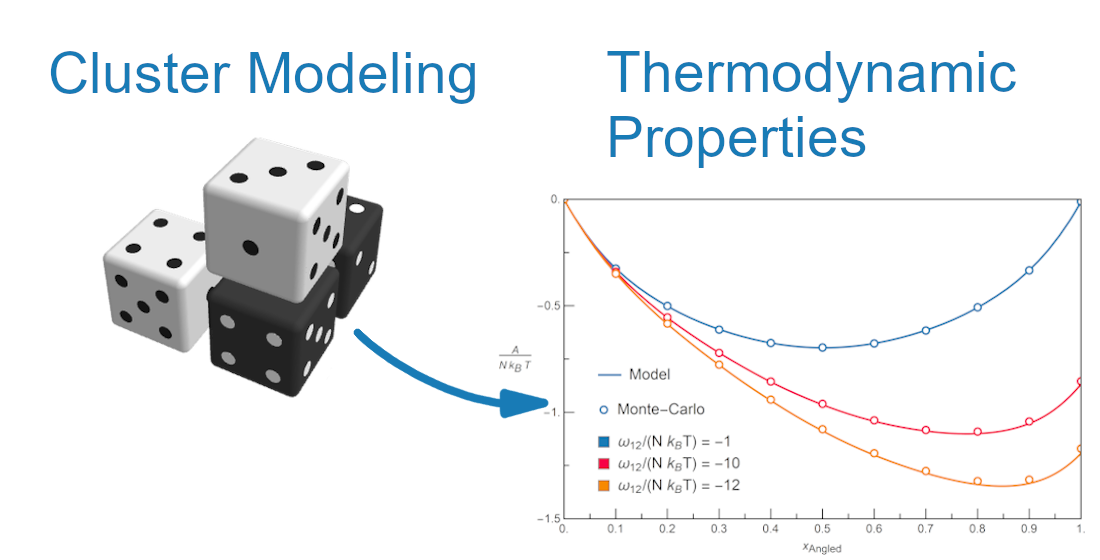

Supplement: Supplementary file 1 [file entropy-22-01111-s001.zip › Supplementary Files/Graphics/Graphical Abstract.png]

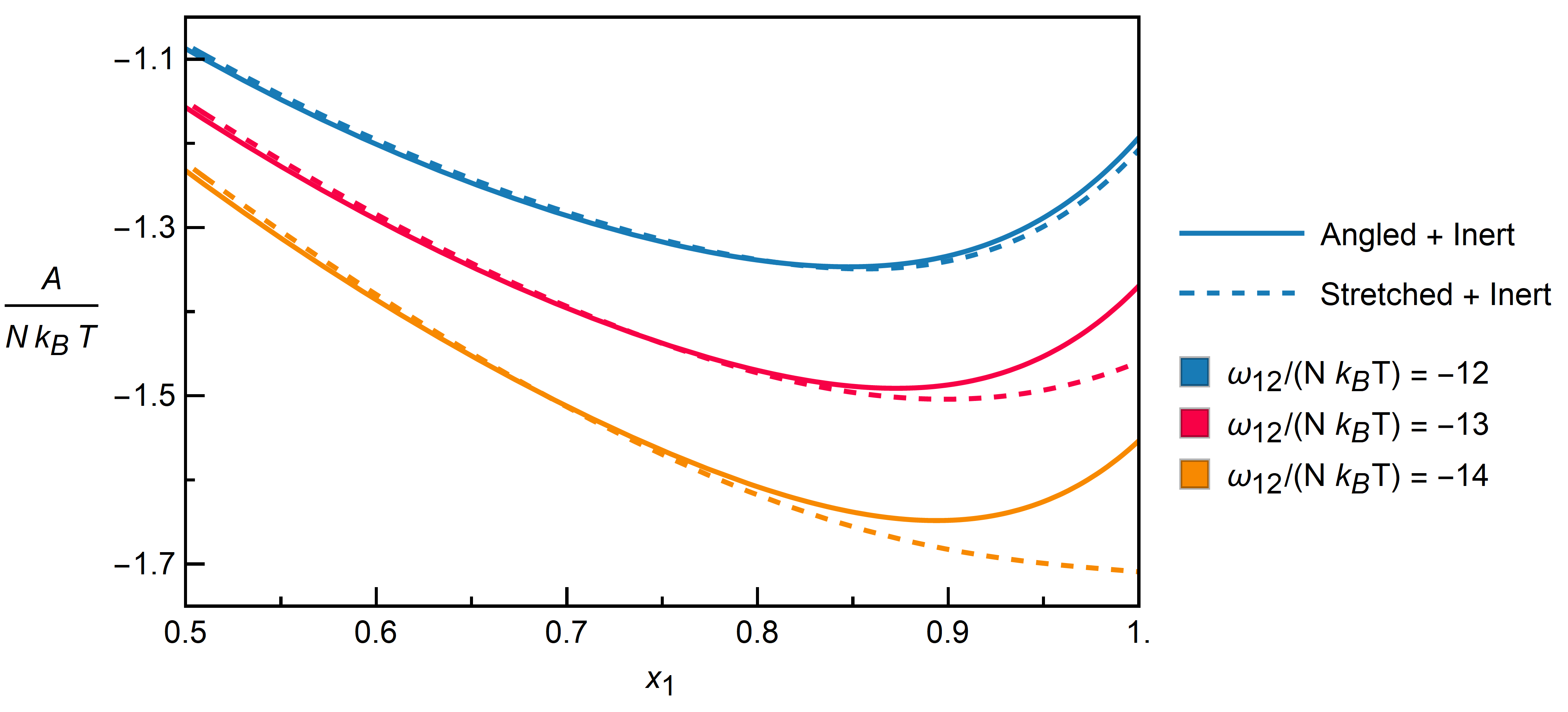

Supplement: Supplementary file 1 [file entropy-22-01111-s001.zip › Supplementary Files/Graphics/IsomerDistinction.png]

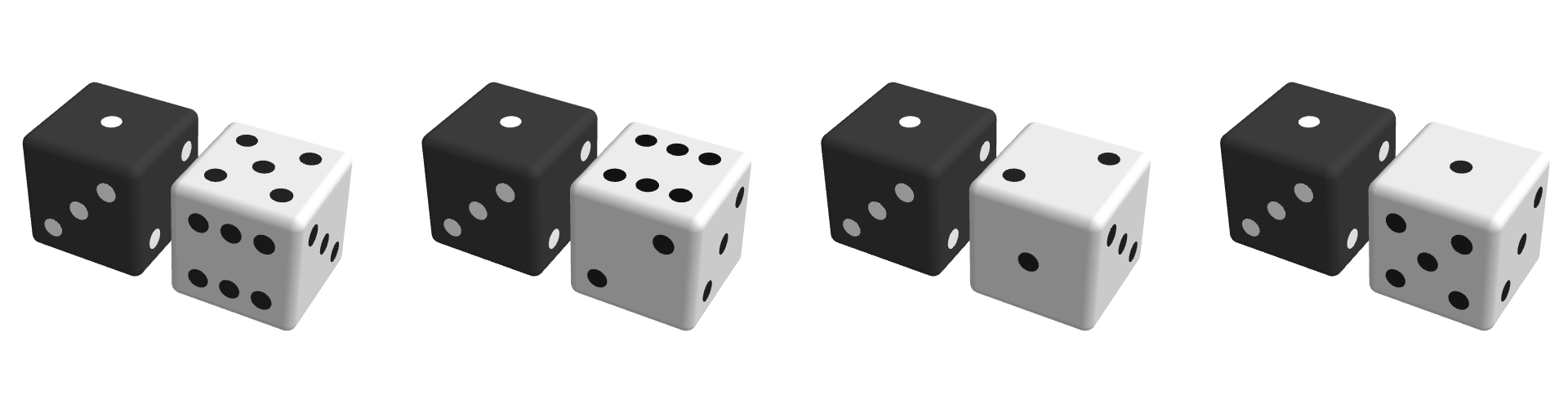

Supplement: Supplementary file 1 [file entropy-22-01111-s001.zip › Supplementary Files/Graphics/PairContactSymmetry.png]

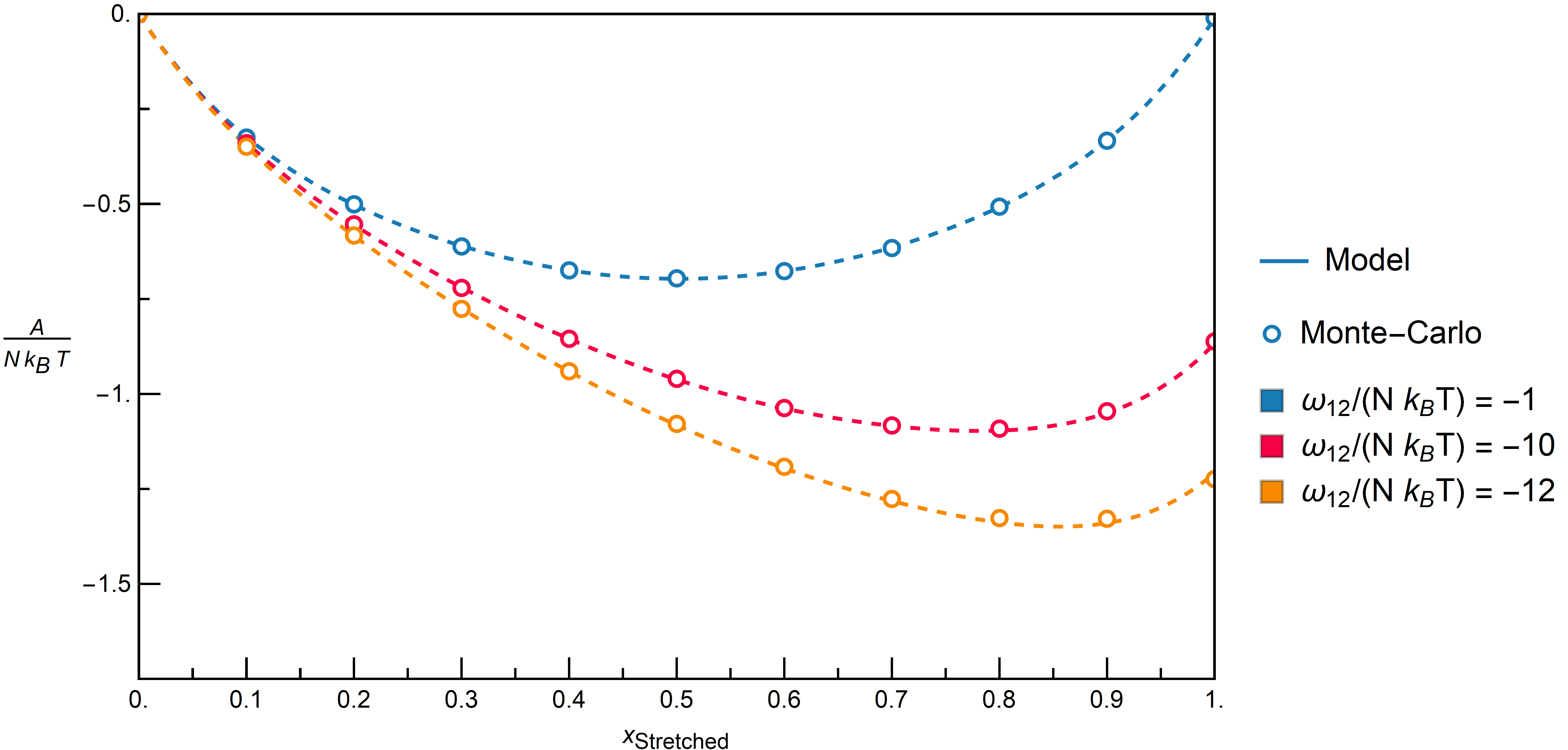

Supplement: Supplementary file 1 [file entropy-22-01111-s001.zip › Supplementary Files/Graphics/StretchedInert.png]
